# Phenolic metabolism in *Sarcandra glabra* is mediated by distinct BAHD hydroxycinnamoyltransferases

**DOI:** 10.1111/tpj.70035

**Published:** 2025-03-03

**Authors:** Paul Bömeke, Maike Petersen

**Affiliations:** ^1^ Institut für Pharmazeutische Biologie und Biotechnologie Philipps‐Universität Marburg Robert‐Koch‐Str. 4 Marburg 35037 Germany

**Keywords:** hydroxycinnamoyltransferases, BAHD, *Sarcandra glabra* (Chloranthaceae), Chloranthales, phenolic metabolism, phenylpropanoid derivatives, chlorogenic acid, cryptochlorogenic acid, caffeoylshikimic acid, rosmarinic acid

## Abstract

*Sarcandra glabra* (Chloranthaceae) has an elaborate phenolic metabolism, encompassing various hydroxycinnamic acid esters. This may imply that multiple hydroxycinnamoyltransferases are involved in establishing this spectrum of natural compounds. Five coding sequences from *S. glabra*, belonging to the superfamily of BAHD acyltransferases, have been amplified from *S. glabra* cDNA, and the proteins were expressed in *Escherichia coli*. By assaying the proteins biochemically, the main substrates of these enzymes were identified as *p*‐coumaroyl‐ and caffeoyl‐CoA as donor substrates together with varying acceptor substrates. SgHST mainly forms *p*‐coumaroyl‐ and caffeoylshikimic acid, but also the corresponding quinic acid esters as well as amides with 3‐ and 5‐hydroxyanthranilic acids. SgHQT1 predominantly catalyzes the formation of *p*‐coumaroyl‐ and caffeoyl‐5‐*O*‐quinic acid, while SgHQT2 correspondingly forms *p*‐coumaroyl‐ and caffeoyl‐4‐*O*‐quinic acid. To our knowledge, this is the first characterized enzyme forming cryptochlorogenic acid and its precursor *p*‐coumaroyl‐4‐*O*‐quinic acid. SgRAS synthesizes rosmarinic acid and its precursors (caffeoyl‐4′‐hydroxyphenyllactic, *p*‐coumaroyl‐4′‐hydroxyphenyllactic, *p*‐coumaroyl‐3′,4′‐dihydroxyphenyllactic acids) as well as amides with aromatic d‐amino acids. No substrates could be identified for the fifth sequence, SgHCT‐F, which phylogenetically groups with benzyl alcohol *O*‐benzoyltransferases. All enzymes, except SgHCT‐F, were fully kinetically characterized, and their expression in different tissues of *S. glabra* was assessed.

## Significance Statement

Four genes and enzymes involved in the formation of hydroxycinnamoyl esters with shikimic, quinic, hydroxyphenyllactic, and benzoic acids as well as amides with hydroxyanthranilic and d‐amino acids were identified in *Sarcandra glabra* (Chloranthaceae), placed in a sister clade of the magnoliids. One enzyme catalyzes the direct formation of cryptochlorogenic acid, which has not been previously described.

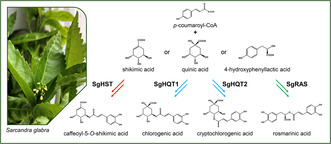

## INTRODUCTION

*Sarcandra glabra* (Thunb.) Nakai is a perennial, evergreen species of the family Chloranthaceae, occurring in Southeast Asia, China, and Japan. The Chloranthaceae is the only family in the order Chloranthales, which belongs to the mesangiosperms and is considered a sister group to the magnoliids (Guo et al., 2021). It encompasses only four genera, *Ascarina*, *Chloranthus*, *Hedyosmum*, and *Sarcandra*, with a total of about 75 species (Stevens, 2001). The genus *Sarcandra* comprises only the species *S. glabra*, with a number of subspecies or varieties (https://www.worldfloraonline.org/taxon/wfo‐0000438024; Accessed 13th January 2025).

The herb (above‐ground plant parts) of *S*. *glabra* (Sarcandrae herba, zhong jie feng) is traditionally used as a medicinal plant but also as an infusion due to its aromatic taste (Zeng et al., 2021). The drug and its uses have been enumerated by Wagner et al. (2015), who listed antioxidative, anti‐tumor, cytotoxic, antimicrobial, anti‐inflammatory, and hepatoprotective effects. The manifold health‐beneficial effects (summarized by Chu et al., 2023; Zeng et al., 2021) can be attributed to the approximately 400 chemical compounds that have been identified in this species to date. The most prominent groups of specialized compounds are terpenoids (sesqui‐, di‐, triterpenes), flavonoids, coumarins, lignans, anthraquinones, and phenolic acids, as well as phenolic acid esters such as chlorogenic acid (CA), caffeic acid 3,4‐dihydroxyphenethyl ester, and rosmarinic acid (RA) (Chu et al., 2023; Li et al., 2011; Zeng et al., 2021; Zhang et al., 2019). RA is well‐known as a compound characteristic for Boraginaceae and the sub‐family Nepetoideae of the Lamiaceae but has meanwhile been found in many genera throughout the plant kingdom (Petersen et al., 2009; Petersen & Simmonds, 2003). In *S*. *glabra*, it has been detected by Huang et al. (2007). Another genus of the Chloranthaceae, *Chloranthus*, has been reported to accumulate RA as well. Interestingly, from two investigated species, *C. officinalis* contained RA as well as CA, while in *C. spicatus* only CA was found (Petersen et al., 2009). Based on fossil finds dating back to the Lower Cretaceous period (Stevens, 2001), an early existence of the Chloranthaceae can accordingly be assumed. The Chloranthales are considered a sister clade to magnoliids (Stevens, 2001) and branch off early in the spermatophyte phylogenetic tree; thus, the occurrence of RA and other hydroxycinnamic acid derivatives is interesting from an evolutionary point of view. Besides in angiosperms, RA has been reported from hornworts and some fern species of the Blechnaceae family (Bohm, 1968; Takeda et al., 1990), while, to our knowledge, it is not known from gymnosperms and members of the “basal orders” of the magnoliopsida (Amborellales, Nymphaeales, Austrobaileyales) as well as the neighboring orders of the magnoliids (Magnoliales, Laurales, Canellales, Piperales), except for one tentative detection in *Nymphaea nouchali* tubers (Uddin et al., 2020). On the other hand, RA has been described from many orders or families of the mono‐ and eudicotyledonous angiosperms (Petersen et al., 2009). The question therefore arises whether convergent evolution towards the formation of RA and other hydroxycinnamic acid derivatives might have taken place several times independently.

Hydroxycinnamic acid esters detected in *S*. *glabra* are RA and its 4‐*O*‐β‐glucoside, as well as methyl‐ and ethylrosmarinic acid, caffeic acid 3,4‐dihydroxyphenethyl ester, CA, neo‐ and cryptochlorogenic acid, and caffeoyl‐3‐, ‐4‐, and ‐5‐*O*‐shikimic acid (Zeng et al., 2021).

CA and RA are commonly synthesized by hydroxycinnamoyltransferases (HCT), which use coenzyme A‐activated hydroxycinnamic acids together with acceptor molecules, such as hydroxyphenyllactic, shikimic, or quinic acids, but also, e.g., spermidine, malic, or anthranilic acid derivatives can be used by HCTs. HCTs belong to the BAHD superfamily of acyltransferases, first described by St. Pierre and de Luca (2000), who coined the name BAHD from the first four enzymes of this family characterized in more detail. Since this time, the enzyme family has proven to be a key player in primary and specialized metabolism (Moghe et al., 2023).

The three‐dimensional structure of HCT proteins is organized in two domains linked by a loop with varying lengths. Crystal structures have shown the donut‐like structure of this enzyme family with the active center in a channel going through the enzyme. The catalytic histidine residue within the conserved HxxxDG motif is placed in this channel. The reaction mechanism has been studied for hydroxycinnamoyl‐CoA:shikimic acid HCT (HST) from *Sorghum bicolor*, which forms *p*‐coumaroyl‐3‐*O*‐shikimic acid (Walker et al., 2013). During catalysis, a proton is abstracted from C3‐OH of shikimic acid by the N_Ɛ2_ atom of the catalytic histidine. Afterwards, a nucleophilic attack of the formed oxyanion on the γ‐C of *p*‐coumaroyl‐CoA takes place, and coenzyme A is released after protonation. Additional calorimetric data suggest that *p*‐coumaroyl‐CoA binds prior to the acceptor substrate. Another conserved motif, DFGWG, is located more outside near to the *C*‐terminus and is not directly involved in catalysis. A structure‐stabilizing property has been assigned to this motif (Walker et al., 2013).

The formation of *p*‐coumaroylshikimic acid, followed by *meta*‐hydroxylation to caffeoylshikimic acid, is an important step towards the formation of monolignols with a more elaborated substitution pattern at the aromatic ring (caffeyl, coniferyl, and sinapyl alcohols), the precursors for lignin and lignans. Caffeoylshikimic acid is then cleaved to shikimic acid and caffeoyl‐CoA or caffeic acid, respectively, catalyzed by HCTs or caffeoylshikimic acid esterases (CSE) (Hoffmann et al., 2004; Kruse et al., 2024; Shadle et al., 2007; Vanholme et al., 2013). Thus, hydroxycinnamoylshikimic acid esters are rarely accumulated to a high extent in plants, but three isoforms (caffeoyl‐3‐, ‐4‐, and ‐5‐*O*‐shikimic acid) have been detected in *S*. *glabra* (Zeng et al., 2021). On the other hand, chlorogenic acid derivatives, the esters of caffeic and quinic acid, have been found in high concentrations in many plant species (Clifford et al., 2017). Three isoforms of these esters have been isolated from *S. glabra* (Zeng et al., 2021), the most common of which is chlorogenic acid (CA, caffeoyl‐5‐*O*‐quinic acid). The other regioisomers are cryptochlorogenic acid (cCA, caffeoyl‐4‐*O*‐quinic acid) and neochlorogenic acid (nCA, caffeoyl‐3‐*O*‐quinic acid). It has been supposed that after the formation of chlorogenic acid, the other isomers might form spontaneously by acyl migration (Xie et al., 2011). Besides, various dicaffeoylquinic acid derivatives occur in plants (Clifford et al., 2017) but have not been discovered in *S. glabra* extracts. It should be stated here that the nomenclature of caffeoylquinic acids remains inconsistent, since chlorogenic acid has first been described as caffeoyl‐3‐*O*‐quinic acid (Fischer & Dangschat, 1932), but the name proposed by IUPAC is caffeoyl‐5‐*O*‐quinic acid (Alcázar Magaña et al., 2021; Clifford et al., 2017). For the nomenclature used in this report, please refer to Figure S1.

The biosynthetic pathway towards RA in the Lamiaceae species *Coleus blumei* has been unraveled by Petersen et al. (1993) and has been confirmed in other Lamiaceae species. For *S. glabra*, an RA biosynthetic pathway was recently proposed based on isolated transcript sequences and subsequent functional analysis (Li et al., 2024). The biochemical background for the formation of *p*‐coumaroyl‐ or caffeoylshikimic acid and *p*‐coumaroyl‐ or caffeoylquinic acid derivatives, as well as detailed functional characterization of HCTs, has not yet been assessed in this species. Investigations on the specific enzymatic formation of the 3‐*O*‐ and 4‐*O*‐regioisomers of caffeoylshikimic, *p*‐coumaroylshikimic, caffeoylquinic, and *p*‐coumaroylquinic acids are mostly missing to date. Recently, however, a BAHD hydroxycinnamoyltransferase from *Bambusa multiplex* was identified after inducing its expression with a histone deacetylase inhibitor, forming *p*‐coumaroyl‐3‐*O*‐shikimic acid (Nomura et al., 2022).

We here describe the PCR amplification of five BAHD acyltransferase sequences identified in the *S*. *glabra* transcriptome followed by heterologous expression in *Escherichia coli* and the biochemical characterization of four of the encoded enzymes, showing various biosynthetic activities on the way to hydroxycinnamoylshikimic, hydroxycinnamoylquinic, and rosmarinic acids.

## RESULTS

### Phytochemical analysis of *p*‐coumaroyl and caffeoyl derivatives in *S. glabra*

**Figure 1:**
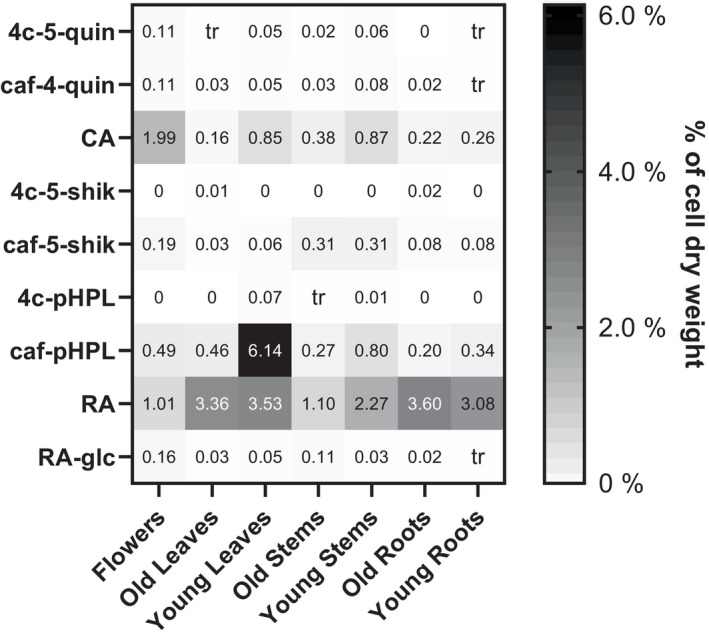
Amount of *p*‐coumaroyl and caffeoyl derivatives in extracts from various tissues of *Sarcandra glabra* in % of the dry weight (*n* = 3) depicted as a color gradient from white (0%) to black (6.0%). Nine out of a total of 17 substances are displayed. Compounds occurring in traces with a content below 0.01% are marked with “tr,” whereas 0 stands for “not detected.” The detailed table of all identified compounds from *Sarcandra glabra* can be accessed in Table S1. 4c‐5‐quin, *p*‐coumaroyl‐5‐*O*‐quinic acid; 4c‐5‐shik, *p*‐coumaroyl‐5‐*O*‐shikimic acid; 4c‐pHPL, *p*‐coumaroyl‐4′‐hydroxyphenyllactic acid; CA, chlorogenic acid = caffeoyl‐5‐*O*‐quinic acid; caf‐4‐quin, caffeoyl‐4‐*O*‐quinic acid; caf‐5‐shik, caffeoyl‐5‐*O*‐shikimic acid; caf‐pHPL, caffeoyl‐4′‐hydroxyphenyllactic acid; RA, rosmarinic acid = caffeoyl‐3′,4′‐dihydroxyphenyllactic acid; RA‐glc, rosmarinic acid glycoside.

Plant material (flowers, young and old leaves, young and old stems, young and old roots) of our *S*. *glabra* plants was harvested in winter 2023 in order to get an overview of the hydroxycinnamoyl derivatives and their quantities in different plant organs. The plant material was lyophilized, and the pulverized material was extracted with 70% (v/v) aqueous ethanol. The extracts were subsequently analyzed by HPLC (see data in Tables S1 and S2) and LC–MS (see data in Table S3). Our analyses showed that RA and caffeoyl‐4′‐hydroxyphenyllactic acid (caf‐pHPL = isorinic acid) were the most abundant compounds with 6.14% of the dry weight for caf‐pHPL in young leaves and 1.01% (flowers) to 3.60% (old roots) for RA in all plant parts (Figure 1; Table S1). Other RA precursors such as caffeoyl‐4′‐hydroxyphenyllactic, *p*‐coumaroyl‐4′‐hydroxyphenyllactic, and *p*‐coumaroyl‐3′,4′‐dihydroxyphenyllactic acids were also detected but at rather low levels. All three positional isomers of *p*‐coumaroyl‐ and caffeoylquinic acid were detectable, with chlorogenic acid (CA, caffeoyl‐5‐*O*‐quinic acid) being the most abundant, with levels up to 1.99% of the dry weight in flowers. Caffeoyl‐5‐*O*‐shikimic acid was highest with 0.31% in young and old stems. A glucoside of RA in *S*. *glabra* was previously described as rosmarinic acid 4′‐*O*‐β‐d‐glucoside (Duan et al., 2010). A glycoside of RA has been detected here as well with up to 0.16% of the dry weight in flowers, but the position of sugar attachment was not determined.

### Identification of BAHD acyltransferase sequences in *S. glabra*

In order to identify sequences encoding BAHD acyltransferases, the 1kP transcriptome database (https://db.cngb.org/onekp/) was queried with the amino acid sequence of rosmarinic acid synthase (RAS) from *C. blumei* (Lamiaceae; UniProt A0PDV5). BLASTP searches revealed 45 putative scaffolds, from which only the first six sequences were taken into further consideration (Table S4a), as these sequences showed the lowest *e*‐values and high percentual identities to the query sequence (cutoff value: *e*‐value 1.0 × 10^−50^) (Table S4). The following scaffolds were taken into consideration: OSHQ‐2009492 = SgHCT‐A, OSHQ‐2009493 = SgHCT‐B, OSHQ‐2009494 = SgHCT‐C, OSHQ‐2009853 = SgHCT‐E (partial), OSHQ‐2048693 = SgHCT‐D, OSHQ‐2048698 = SgHCT‐F. From this point on, the sequences or enzymes, which are the subject of this work, will be referenced according to the names as they were entered in GenBank (see Table S4). SgHCT‐A is SgHST, SgHCT‐C is SgHQT1, SgHCT‐E is SgHQT2, and SgHCT‐D is SgRAS. As no substrates could be identified for SgHCT‐F, it is still referred to as SgHCT‐F. The sequence of SgHCT‐B was composed of the front part of scaffold OSHQ‐2008494 (SgHQT1) and the rear part of scaffold OSHQ‐2009492 (SgHST). Since it could not be amplified by PCR, it was considered to be incorrectly assembled. The full‐length sequence of SgHQT2 had to be attained by 5′‐RACE‐PCR. All HCTs, except SgHCT‐B, were readily amplified from cDNA of *S*. *glabra* leaves, showing the expression of the respective genes. When comparing the amino acid sequences between each other by EMBOSS Needle, the identities ranged from 28 to 70% (Table S5). The highest level of identity of 69.6% with a similarity of 83.1% was found for SgHST and SgHQT1, while SgHCT‐F was the most different sequence compared to the other four sequences. In alignments (Figure S2), the conserved sequence motifs HxxxDG and DFGWG are present in all sequences, except SgHCT‐F, where the first motif is HxxxDA, and SgHQT2, where DFGYG is found instead of DFGWG.

### Phylogenetic analysis

Phylogenetic analysis was performed with a series of BAHD hydroxycinnamoyltransferases (see Table S6) to establish a Maximum Likelihood tree with 1000 bootstraps. Information regarding the suggested or proven substrates of the encoded enzymes was included, if possible. Based on the phylogenetic analysis of BAHD hydroxycinnamoyltransferases (Moghe et al., 2023), four clades could be clearly distinguished (Figure S3). Clade 5a is divided into several subclades, one of them, shaded red, consisting of HCTs mainly accepting shikimic, quinic, and (hydroxy)anthranilic acids and therefore could be named HSQTs (hydroxycinnamoyl‐CoA:shikimic acid/quinic acid hydroxycinnamoyltransferase). Enzymes, which mainly take shikimic acid as acceptor substrates, are largely organized according to angiosperm phylogeny. In this subclade, two homogeneous groups can be distinguished, consisting of HSTs from bryophytes and lycophytes and grasses, respectively. SgHST is closest to an HCT from *Pinus radiata* (Pinaceae) and embedded in the group of HSQTs. HQTs, highlighted in blue, are organized in a separate subclade, but SgHQT1 and SgHQT2 are not part of it. SgHQT1 is closer to the shikimic acid‐accepting subclade but forms a separate branch. The same is true for SgHQT2, which is proximal to the HST and HQT subclades but placed on a separate branch. A third subclade of clade 5a is formed by Lamiaceae RAS, HCTs accepting phenyllactic acids, and is marked with a green background. The majority of HCTs in clade 5c take spermine or spermidine as an acceptor substrate, which is highlighted in purple. This clade also includes HCTs accepting malic acid, l‐amino acids, or piscidic acid. A separate branch of clade 5c is built by SgRAS, which accepts phenyllactic acids. At the lower end of the phylogenetic tree, clade 5b, consisting of only three representatives from Poaceae, and HCTs from clade 3, 6a and 6c are placed. SgHCT‐F is a member of clade 6c between two HCTs accepting benzyl alcohol as an acceptor, which is shown in yellow. However, our investigations so far could not show activity of SgHCT‐F with benzyl alcohol.

### Expression of SgHCT sequences in *Escherichia coli*

All SgHCT sequences were ligated into the expression vector pET‐15b and transferred into the expression strain *E*. *coli* SoluBL21. The bacterially synthesized proteins were purified by metal chelate chromatography with the help of the *N*‐terminally attached 6xHis‐tag. SDS‐PAGE and Western blot analyses proved the presence of the heterologously expressed proteins (Figure S4).

**Figure 2:**
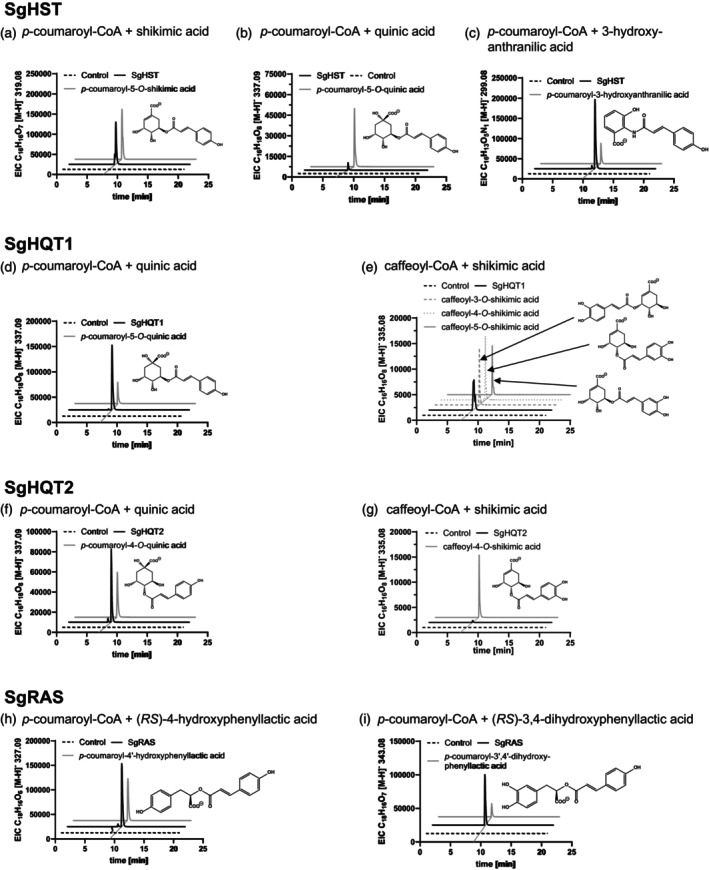
Selected extracted ion chromatograms (EIC) of enzyme assays with SgHST, SgHQT1, SgHQT2, and SgRAS. EICs from all products are shown in Tables S7–S11. The molecular formula of the expected product and the expected ion with the respective mass‐to‐charge ratio (*m*/*z*) is given on the *Y*‐axis. The black line represents the EIC of an enzyme assay with the respective SgHCT, donor, and acceptor substrate. The dashed black line (Control) refers to the EIC of an empty vector control assay, which was conducted under the same conditions in parallel. Authentic standards (0.25 nmol) are depicted with a bold gray line, if they were available. A thin gray line shows the shift of the chromatograms, based on the retention times of the peaks. The structural formula refers to the product, which is indicated by the thin gray line. (a) EIC of an assay with SgHST resulting in *p*‐coumaroyl‐5‐*O*‐shikimic acid ([M‐H]^−^ *m*/*z* 319.08). (b) EIC of an assay with SgHST resulting in *p*‐coumaroyl‐5‐*O*‐quinic acid ([M‐H]^−^ *m*/*z* 337.09). (c) EIC of an assay with SgHST resulting in *p*‐coumaroyl‐2‐*N*‐3‐hydroxyanthranilic acid ([M‐H]^−^ *m*/*z* 299.08). (d) EIC of an assay with SgHQT1 resulting in *p*‐coumaroyl‐5‐*O*‐quinic acid ([M‐H]^−^ *m*/*z* 321.10). (e) EIC of an assay with SgHQT1 resulting in caffeoyl‐3‐, ‐4‐ and ‐5‐*O*‐shikimic acid ([M‐H]^−^ *m*/*z* 335.08); the retention times are almost indistinguishable, but the product peak shows three tips. An HPLC chromatogram, which shows separation of the three peaks is displayed in Figure S6. (f) EIC of an assay with SgHQT2 resulting in *p*‐coumaroyl‐4‐*O*‐quinic acid ([M‐H]^−^ *m*/*z* 337.09). (g) EIC of an assay with SgHQT2 resulting in caffeoyl‐4‐*O*‐shikimic acid ([M‐H]^−^ *m*/*z* 335.08). (h) EIC of an assay with SgRAS resulting in *p*‐coumaroyl‐4′‐hydroxyphenyllactic acid ([M‐H]^−^ *m*/*z* 327.09). (i) EIC of an assay with SgRAS resulting in *p*‐coumaroyl‐3′,4′‐dihydroxyphenyllactic acid ([M‐H]^−^ *m*/*z* 343.08). Authentic standards are described in Tables S17 and S18.

The open reading frame of SgHST (GenBank PP449349) encompasses 1311 nucleotides coding for a protein of 436 amino acid residues with a molecular mass of 48 424 Da (without the attached 6xHis‐tag) and an isoelectric point of pH 6.89. The coding sequence is not interrupted by introns; however, the first 46 bp could not be analyzed due to the position of the forward primer used for amplification from genomic DNA. The enzyme catalyzes the transfer of a hydroxycinnamoyl moiety from hydroxycinnamoyl‐CoA (preferably *p*‐coumaroyl‐ and caffeoyl‐CoA, but also cinnamoyl‐, feruloyl‐, and sinapoyl‐CoA) to shikimic acid, forming *p*‐coumaroyl‐/caffeoyl‐5‐*O*‐shikimic acid (see Table S7; Figure 2a–c; Figure S1) or the other respective hydroxycinnamic acid esters. Acceptor substrates accepted with lower efficiency are quinic acid, 3‐ and 5‐hydroxyanthranilic acid (with amides as products), as well as 3‐hydroxybenzoic, 2,3‐dihydroxybenzoic, 2,5‐dihydroxybenzoic (gentisic), 3,4‐dihydroxybenzoic (protocatechuic), and 3‐aminobenzoic acid (Figure S5). Other putative substrates tested for acceptance are shown in Table S8. The enzyme thus qualifies to be a hydroxycinnamoyl‐CoA:shikimic acid hydroxycinnamoyltransferase (SgHST; E.C. number 2.3.1.133). In the phylogenetic tree (Figure S3), SgHST groups in a large subgroup of clade 5a (as defined by Moghe et al., 2023) together with other hydroxycinnamoyltransferases using shikimic acid and sometimes also quinic acid and anthranilic acid derivatives as substrates.

SgHQT1 (GenBank PP449350) has an open reading frame of 1284 nucleotides and does not contain introns in the genomic sequence. The encoded protein has a length of 427 amino acid residues, a molecular mass of 47 772 Da, and an isoelectric point at pH 6.56. The enzyme preferably uses quinic acid as an acceptor for hydroxycinnamoyl units and mainly forms *p*‐coumaroyl‐/caffeoyl‐5‐*O*‐quinic acid (= chlorogenic acid [CA]) (see Table S9; Figure 2d,e; Figure S1), but also the cinnamic, ferulic, and sinapic acid derivatives. Enzyme assays with shikimic acid resulted in three peaks, which were identified as *p*‐coumaroyl‐/caffeoyl‐3‐, ‐4‐, and ‐5‐*O*‐shikimic acid (see Figures S1 and S6). The formation of a dicaffeoylshikimic acid derivative could be observed after prolonged incubation with caffeoyl‐CoA and high concentrations of shikimic acid (Figure S7; Table S9). Substrates with lower turnover are 3‐ and 5‐hydroxyanthranilic, 4‐hydroxybenzoic, 2,3‐dihydroxybenzoic, 2,4‐dihydroxybenzoic, 2,5‐dihydroxybenzoic (gentisic), 3,4‐dihydroxybenzoic (protocatechuic), 3‐aminobenzoic acids, methanol, and glycerol (Tables S8 and S9; Figure S7). SgHQT1 thus is a hydroxycinnamoyl‐CoA:quinic acid hydroxycinnamoyltransferase (E.C. number 2.3.1.99). The amino acid sequence is also grouped in subclade 5a, although on an isolated branch (Figure S3).

SgHQT2 (GenBank PP449352) contains an open reading frame of 1326 nucleotides, while the genomic sequence could not be identified. The encoded protein has a length of 441 amino acid residues, a molecular mass of 48 847 Da, and an isoelectric point at pH 7.13. Interestingly, this enzyme transfers the hydroxycinnamoyl moiety from *p*‐coumaroyl‐/caffeoyl‐CoA, or cinnamoyl‐, feruloyl‐, sinapoyl‐CoA as donor substrates with lower efficiency, to the 4‐hydroxy group of quinic acid, thus forming cryptochlorogenic acid (cCA; see Table S10; Figure 2f,g; Figure S1) with caffeoyl‐CoA and quinic acid. Shikimic, 4‐hydroxybenzoic and 2,4‐dihydroxybenzoic acids were taken as acceptors as well (Table S8). In the phylogenetic tree (Figure S3), this sequence clusters with enzymes accepting mainly quinic acid and also shikimic acid, but it is at the margin on a separate branch.

The open reading frame, also not interrupted by introns, of SgRAS (GenBank PP449351) has a length of 1350 nucleotides and encodes a polypeptide of 449 amino acid residues. The molecular mass is at 49348 Da, and the isoelectric point of the protein is at pH 5.82. Besides accepting the same donor substrates, mainly *p*‐coumaroyl‐ and caffeoyl‐CoA, and cinnamoyl‐, feruloyl‐, and sinapoyl‐CoA to a lower extent, this enzyme uses 4‐hydroxyphenyllactic acid and 3,4‐dihydroxyphenyllactic acid as primary acceptor substrates. Thus, SgRAS can form rosmarinic acid and its precursors *p*‐coumaroyl‐4′‐hydroxyphenyllactic, caffeoyl‐4′‐hydroxyphenyllactic, *p*‐coumaroyl‐3′,4′‐dihydroxyphenyllactic acids (see Table S11; Figure 2h,i; Figure S1). Further acceptors are (*R*)‐3‐phenyllactic acid, (*R*)‐4‐hydroxy‐3‐methoxyphenyllactic acid, as well as d‐phenylalanine, d‐tyrosine and, to a very low extent, d‐dihydroxyphenylalanine, whereas (*S*)‐4‐hydroxyphenyllactic acid and l‐amino acids are not accepted (see Figure S9; Table S8). This enzyme is considered to be a hydroxycinnamoyl‐CoA:hydroxyphenyllactic acid hydroxycinnamoyltransferase (E.C. number 2.3.1.140). SgRAS interestingly does not cluster with other known RAS sequences from Lamiaceae (Figure S3), but is closer to clade 5c, which contains a group of spermidine HCTs and RAS from *Phacelia campanularia* (Boraginaceae) (Levsh et al., 2019).

SgHCT‐F (GenBank PQ336776) has a length of 1377 nucleotides. The genomic sequence contains an intron of 91 bp after 445 bp. This widely conserved “Q‐intron” (as defined by St. Pierre & de Luca, 2000) is inserted after the codon standing for glutamine (Q) and is positioned 17 amino acid residues upstream of the conserved HxxxDG motif, which is altered to HTISDA in SgHCT‐F. The encoded protein has a length of 458 amino acid residues, a molecular mass of 50 538 Da and an isoelectric point at pH 7.13. To date, substrate searches did not lead to the identification of donor and acceptor substrates (for tested substrates, see Table S8). BLASTP was used for similarity searches, and these searches find the highest similarities (>75%) with predicted benzoyl‐CoA:benzyl alcohol *O*‐benzoyltransferases (BBT) from various plant species. Accordingly, the phylogenetic placement of this sequence (Figure S3) is within a group of BBTs belonging to clade 6c according to Moghe et al. (2023). To date, however, a formation of benzyl benzoic acid catalyzed by SgHCT‐F could not be observed.

### Kinetic characterization of SgHST

**Figure 3:**
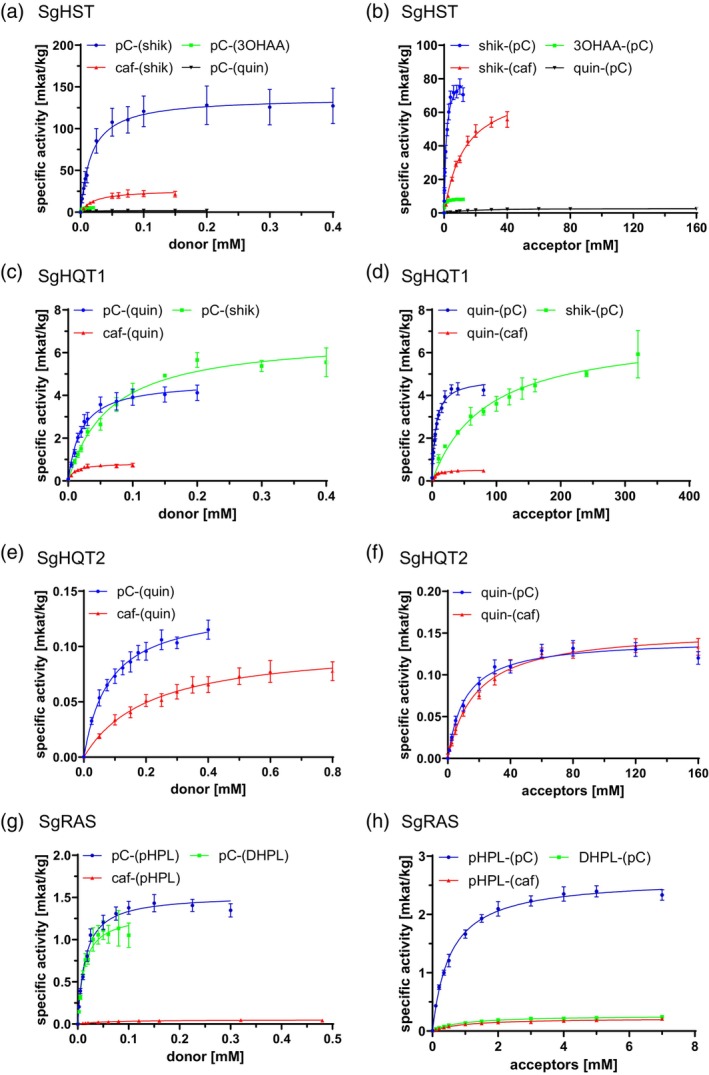
Michaelis–Menten graphs for SgHST, SgHQT1, SgHQT2, and SgRAS, for hydroxycinnamoyl donors and acceptors separately. Substrates in brackets represent the constant substrate. Refer to Figures S10–S13 for a separate display of all graphs. (a) Substrate saturation curves for SgHST with hydroxycinnamoyl donors; blue: *p*‐coumaroyl‐CoA with shikimic acid, red: caffeoyl‐CoA with shikimic acid, green: *p*‐coumaroyl‐CoA with 3‐hydroxanthranilic acid, black: *p*‐coumaroyl‐CoA with quinic acid. (b) Substrate saturation curves for SgHST with hydroxycinnamoyl acceptors: blue: shikimic acid with *p*‐coumaroyl‐CoA, red: shikimic acid with caffeoyl‐CoA, green: 3‐hydroxyanthranilic acid with *p*‐coumaroyl‐CoA, black: quinic acid with *p*‐coumaroyl‐CoA. (c) Substrate saturation curves for SgHQT1 with hydroxycinnamoyl donors: blue: *p*‐coumaroyl‐CoA with quinic acid, red: caffeoyl‐CoA with quinic acid, green: *p*‐coumaroyl‐CoA with shikimic acid. (d) Substrate saturation curves for SgHQT1 with hydroxycinnamoyl acceptors: blue: quinic acid with *p*‐coumaroyl‐CoA, red: quinic acid with caffeoyl‐CoA, green: shikimic acid with *p*‐coumaroyl‐CoA. (e) Substrate saturation curves for SgHQT2 with hydroxycinnamoyl donors: blue: *p*‐coumaroyl‐CoA with quinic acid, red: caffeoyl‐CoA with quinic acid. (f) Substrate saturation curves for SgHQT2 with hydroxycinnamoyl acceptors, blue: quinic acid with *p*‐coumaroyl‐CoA, red: quinic acid with caffeoyl‐CoA. (g) Substrate saturation curves for SgRAS with hydroxycinnamoyl donors: blue: *p*‐coumaroyl‐CoA with 4‐hydroxyphenyllactic acid, red: caffeoyl‐CoA with 4‐hydroxyphenyllactic acid, green: *p*‐coumaroyl‐CoA with 3,4‐dihydroxyphenyllactic acid. (h) Substrate saturation curves for SgRAS with hydroxycinnamoyl acceptors: blue: 4‐hydroxyphenyllactic acid with *p*‐coumaroyl‐CoA, red: 4‐hydroxyphenyllactic acid with caffeoyl‐CoA, green: 3,4‐dihydroxyphenyllactic acid with *p*‐coumaroyl‐CoA.

SgHST is capable of producing esters and amides with different hydroxycinnamoyl‐CoA thioesters, such as cinnamoyl‐, *p*‐coumaroyl‐, caffeoyl‐, feruloyl‐ and sinapoyl‐CoA, but *p*‐coumaroyl‐ and caffeoyl‐CoA are preferred. These hydroxycinnamoyl units are predominantly transferred to the free 5‐*O*‐hydroxyl groups of shikimic acid and quinic acid. The hydroxybenzoic acid derivatives 3‐hydroxybenzoic, 2,3‐dihydroxybenzoic, 2,5‐dihydroxybenzoic (gentisic), 3,4‐dihydroxybenzoic (protocatechuic), as well as 3‐aminobenzoic, 2‐amino‐3‐hydroxybenzoic (3‐hydroxyanthranilic, 3OHAA), and 2‐amino‐5‐hydroxybenzoic (5‐hydroxyanthranilic) acids were taken as well; the latter two presumably led to amide formation (Ernst et al., 2022). In search of the best reaction conditions, SgHST showed the highest activity at pH 7.35 and at 23.2°C (Table 1; Figure S10a,b). Michaelis–Menten kinetics were performed with the donor substrates *p*‐coumaroyl‐CoA and caffeoyl‐CoA, as well as with the acceptor substrates shikimic acid, 3‐hydroxyanthranilic acid, and quinic acid (Table 1; Table S12; Figure 3a,b; Figure S10). The highest catalytic efficiency was observed with *p*‐coumaroyl‐CoA as the donor substrate (398 096 L sec^−1^ mol^−1^ with shikimic acid; 140 344 L sec^−1^ mol^−1^ with 3‐hydroxyanthranilic acid; Table 1; Figure S5b,f), whereas caffeoyl‐CoA was converted only about a sixth as efficiently (63 158 L sec^−1^ mol^−1^; Table 1; Figure S5c). The affinity for *p*‐coumaroyl‐CoA and caffeoyl‐CoA was comparable with shikimic acid as a constant substrate (*K*_m_ 17.4 ± 4.9 and 21.4 ± 6.8 μm, respectively), but the affinity for *p*‐coumaroyl‐CoA increased significantly when switching to quinic acid or 3‐hydroxyanthranilic acid as an acceptor (*K*_m_ 6.6 ± 0.6 and 2.2 ± 0.3 μm, respectively; Table 1). SgHST showed the highest affinity for an acceptor substrate in combination with the best donor substrate, *p*‐coumaroyl‐CoA, with 3‐hydroxyanthranilic acid (*K*_m_ 237.9 ± 19.6 μm) followed by shikimic acid (*K*_m_ 1221.0 ± 152.9 μm) and quinic acid (*K*_m_ 16740.0 ± 1833.0 μm; Table 1). The affinity for shikimic acid declined about tenfold when switching to caffeoyl‐CoA (*K*_m_ 13230.0 ± 1933.0 μm; Table 1). Biochemical characterization led to the conclusion that SgHST primarily produces *p*‐coumaroyl‐5‐*O*‐shikimic acid. Additionally, caffeoyl‐5‐*O*‐shikimic acid may be produced directly. SgHST shows high affinity for 3‐hydroxyanthranilic acid, but 3‐hydroxyanthranilic acid derivatives could not be detected in *S*. *glabra* so far. The production of *p*‐coumaroyl‐5‐*O*‐quinic acid seems to play a minor role.

**Table 1:** Biochemical characterization of hydroxycinnamoyl‐CoA:shikimic acid hydroxycinnamoyltransferase (SgHST) from *Sarcandra glabra* (for reaction conditions see Table S12)

Kinetic data for…	With…	*K*_m_ (μm)	*V*_max_ (mkat kg^−1^)	*k*_cat_ (sec^−1^)	*k*_cat_/*K*_m_ (L sec^−1^ mol^−1^)
*p*‐Coumaroyl‐CoA	Shikimic acid	17.4 ± 4.9	137.30 ± 9.04	6.927	398 096
Caffeoyl‐CoA	Shikimic acid	21.4 ± 6.8	26.79 ± 2.65	1.352	63 158
*p*‐Coumaroyl‐CoA	Quinic acid	6.6 ± 0.6	1.96 ± 0.04	0.099	14 982
*p*‐Coumaroyl‐CoA	3‐Hydroxyanthranilic acid	2.2 ± 0.3	6.12 ± 0.22	0.309	140 344
Shikimic acid	*p*‐Coumaroyl‐CoA	1221.0 ± 152.9	83.70 ± 2.71	4.223	3458
Shikimic acid	Caffeoyl‐CoA	13 230.0 ± 1933.0	77.35 ± 4.77	3.902	295
Quinic acid	*p*‐Coumaroyl‐CoA	16 740.0 ± 1833.0	2.92 ± 0.11	0.147	9
3‐Hydroxyanthranilic acid	*p*‐Coumaroyl‐CoA	237.9 ± 19.6	8.10 ± 0.11	0.409	1718
pH‐optimum	7.35		Temperature optimum	23.2°C	

Values for *K*_m_ and *V*_max_ are based on *n* = 9 ± SEM, *k*_cat_ is calculated with the molecular mass of the protein (including a 6xHis‐tag) of 50.5 kDa.

### Kinetic characterization of SgHQT1 and SgHQT2

SgHQT1 and SgHQT2 were able to use cinnamoyl‐, *p*‐coumaroyl‐, caffeoyl‐, feruloyl‐, and sinapoyl‐CoA to acylate quinic acid. Shikimic acid and various hydroxybenzoic acids were taken as well, but elongated incubation and high concentrations of acceptor substrates were necessary.

SgHQT1 showed the highest turnover at pH 6.81 and at a temperature of 49.4°C (Table 2; Figure S11a,b). Biochemical analyses were performed with the donors *p*‐coumaroyl‐CoA and caffeoyl‐CoA together with the acceptors quinic acid and shikimic acid (Table 2; Table S13; Figure 3c,d; Figure S11). In terms of affinity, caffeoyl‐CoA (*K*_m_ 9.3 ± 2.5 μm) was slightly preferred over *p*‐coumaroyl‐CoA (*K*_m_ 19.9 ± 3.4 μm) with quinic acid as a constant substrate, while a higher catalytic efficiency was found with *p*‐coumaroyl‐CoA (11711 L sec^−1^ mol^−1^ compared to caffeoyl‐CoA) (4444 L sec^−1^ mol^−1^; Table 2; Figure 3c,d; Figure S11). The affinity for *p*‐coumaroyl‐CoA and the respective catalytic efficiency dropped to about one third when changing to shikimic acid as an acceptor (*K*_m_ 64.4 ± 8.5 μm and 5235 L sec^−1^ mol^−1^; Table 2). As indicated by the apparent *K*_m_‐values in tests with *p*‐coumaroyl‐CoA, SgHQT1 preferred quinic acid (*K*_m_ 5520.0 ± 713.0 μm), while shikimic acid was accepted poorly (*K*_m_ 85440.0 ± 18700.0 μm; Table 2). In tests with shikimic acid, the formation of approximately equal amounts of three hydroxycinnamoylshikimic acid derivatives, namely *p*‐coumaroyl‐ or caffeoyl‐3‐, ‐4‐, and ‐5‐*O*‐shikimic acid, could be observed (Figure S6). After prolonged incubation, a peak appeared, which was identified as a dicaffeoylshikimic acid derivative by LC–MS (Figure S7m).

**Table 2:** Biochemical characterization of hydroxycinnamoyl‐CoA:quinic acid hydroxycinnamoyltransferase 1 (SgHQT1) from *Sarcandra glabra* (for reaction conditions see Table S13)

Kinetic data for…	With…	*K*_m_ (μm)	*V*_max_ (mkat kg^−1^)	*k*_cat_ (sec^−1^)	*k*_cat_/*K*_m_ (L sec^−1^ mol^−1^)
*p*‐Coumaroyl‐CoA	Quinic acid	19.9 ± 3.4	4.68 ± 0.24	0.233	11 711
Caffeoyl‐CoA	Quinic acid	9.3 ± 2.5	0.83 ± 0.06	0.041	4444
*p*‐Coumaroyl‐CoA^a^	Shikimic acid	64.4 ± 8.5	6.77 ± 0.31	0.337	5235
Quinic acid	*p*‐Coumaroyl‐CoA	5520.0 ± 713.0	4.81 ± 0.18	0.239	43
Quinic acid	Caffeoyl‐CoA	5769.0 ± 1187.0	0.53 ± 0.03	0.026	5
Shikimic acid^a^	*p*‐Coumaroyl‐CoA	85 440.0 ± 18 700.0	7.01 ± 0.61	0.349	4
pH‐optimum	6.81		Temperature optimum	49.4°C	

Values for *K*_m_ and *V*_max_ are based on *n* = 9 ± SEM or *n* = 3 ± SD (^a^), *k*_cat_ is calculated with the molecular mass of the protein (including a 6xHis‐tag) of 49.8 kDa.

In comparison, SgHQT2 was most active in a broad range between pH 6.87 and 7.42 and at a temperature of 29.5°C (Table 3; Figure S12a,b). As quinic acid was taken as the best acyl acceptor, Michaelis–Menten kinetics were performed with *p*‐coumaroyl‐CoA, caffeoyl‐CoA, and quinic acid (Table 3; Table S14; Figure 3e,f; Figure S12). Regarding the affinity and the catalytic efficiency with acyl donor substrates, *p*‐coumaroyl‐CoA was accepted about three times better (*K*_m_ 82.3 ± 14.8 μm, *k*_cat_/*K*_m_ 87 L sec^−1^ mol^−1^) than caffeoyl‐CoA (*K*_m_ 213.3 ± 53.5 μm, *k*_cat_/*K*_m_ 24 L sec^−1^ mol^−1^) at the same constant concentration of quinic acid (Table 3). Varying the quinic acid concentration at stable concentrations of *p*‐coumaroyl‐ or caffeoyl‐CoA, quinic acid was more affine and was converted faster with *p*‐coumaroyl‐CoA (*K*_m_ 11110.0 ± 1559.0 μm, *k*_cat_/*K*_m_ 0.6 L sec^−1^ mol^−1^) than with caffeoyl‐CoA (*K*_m_ 17730.0 ± 2352.0 μm, *k*_cat_/*K*_m_ 0.4 L sec^−1^ mol^−1^) (Table 3). In contrast to SgHQT1, SgHQT2 primarily addressed the 4‐OH‐group of quinic acid (Figures 2 and 3; Figures S7 and S8).

**Table 3:** Biochemical characterization of hydroxycinnamoyl‐CoA:quinic acid hydroxycinnamoyltransferase 2 (SgHQT2) from *Sarcandra glabra* (for reaction conditions see Table S14)

Kinetic data for…	With…	*K*_m_ (μm)	*V*_max_ (mkat kg^−1^)	k_cat_ (sec^−1^)	*k*_cat_/*K*_m_ (L sec^−1^ mol^−1^)
*p*‐Coumaroyl‐CoA	Quinic acid	82.3 ± 14.8	0.14 ± 0.01	0.007	87
Caffeoyl‐CoA	Quinic acid	213.3 ± 53.5	0.10 ± 0.01	0.005	24
Quinic acid	*p*‐Coumaroyl‐CoA	11110.0 ± 1559.0	0.14 ± 0.004	0.007	0.6
Quinic acid	Caffeoyl‐CoA	17730.0 ± 2352.0	0.15 ± 0.01	0.008	0.4
pH‐optimum	6.87–7.42		Temperature optimum	29.5°C	

Values for *K*_m_ and *V*_max_ are based on *n* = 9 ± SEM, *k*_cat_ is calculated with the molecular mass of the protein (including a 6xHis‐tag) of 51.4 kDa.

In summary, SgHQT1 forms *p*‐coumaroyl‐5‐*O*‐quinic, chlorogenic (CA, caffeoyl‐5‐*O*‐quinic), and *p*‐coumaroyl‐3‐, ‐4‐, and ‐5‐*O*‐shikimic acids, as well as caffeoyl‐3‐, ‐4‐, and ‐5‐*O*‐shikimic acids (Table S9; Figures S6 and S7). Variability in the production of caffeoylshikimic acids resulted in a dicaffeoylshikimic acid derivative. SgHQT2 instead assembles *p*‐coumaroyl‐4‐*O*‐quinic acid, the precursor of cryptochlorogenic acid (cCA), but also cCA acid itself (Figure S8; Table S10). The formation of *p*‐coumaroyl‐4‐*O*‐shikimic acid and caffeoyl‐4‐*O*‐shikimic acid was observed as well (Figure S8; Table S10). To our knowledge, this is the first thoroughly characterized hydroxycinnamoyltransferase forming cCA.

### Kinetic characterization of SgRAS

As stated above, various (hydroxy)cinnamoyl‐CoA thioesters (cinnamoyl‐, *p*‐coumaroyl‐, caffeoyl‐, feruloyl‐, sinapoyl‐CoA) can be used for ester formation by SgRAS together with (hydroxy)phenyllactic acids. The formation of amides with the aromatic d‐amino acids d‐phenylalanine, d‐tyrosine, and d‐DOPA is observed as well, but to a rather low extent. The enzyme exhibits its highest activities at pH 7.4 and at a temperature of 24.6°C (Table 4; Figure S13a,b). Substrate saturation curves were recorded for *p*‐coumaroyl‐CoA and caffeoyl‐CoA as donor substrates and 4‐hydroxyphenyllactic acid (pHPL) and 3,4‐dihydroxyphenyllactic acid (DHPL) as acceptor substrates (Table 4; Table S15; Figure 3g,h; Figure S13). The affinities were highest for *p*‐coumaroyl‐CoA together with either pHPL or DHPL (*K*_m_ *p*‐coumaroyl‐CoA 14.6 ± 1.7 and 12.6 ± 2.5 μm, respectively; Table 4). Here, also the catalytic efficiencies (5398 L sec^−1^ mol^−1^ with pHPL, 5396 L sec^−1^ mol^−1^ with DHPL) were significantly higher than with other substrate combinations (Table 4). The *K*_m_‐value for caffeoyl‐CoA was about three times higher (*K*_m_ 57.2 ± 15.7 μm) and the catalytic efficiency was about a hundred times lower (43 L sec^−1^ mol^−1^; Table 4). The affinity for pHPL (*K*_m_ 543.6 ± 47.4 μm) together with the best donor substrate was higher than for DHPL (*K*_m_ 866.8 ± 104.8 μm; Table 4). Concluding from these *in vitro* data, SgRAS is supposed to form primarily *p*‐coumaroyl‐4′‐hydroxyphenyllactic acid but can also form *p*‐coumaroyl‐3′,4′‐dihydroxyphenyllactic acid. The formation of the caffeoyl substitution pattern must then be finalized by *meta*‐hydroxylation of the *p*‐coumaroyl esters.

**Table 4:** Biochemical characterization of hydroxycinnamoyl‐CoA:hydroxyphenyllactic acid hydroxycinnamoyltransferase (SgRAS) from *Sarcandra glabra* (for reaction conditions see Table S15)

Kinetic data for…	With…	*K*_m_ (μm)	*V*_max_ (mkat kg^−1^)	*k*_cat_ (sec^−1^)	*k*_cat_/*K*_m_ (L sec^−1^ mol^−1^)
*p*‐Coumaroyl‐CoA	pHPL	14.6 ± 1.7	1.53 ± 0.04	0.079	5398
Caffeoyl‐CoA	pHPL	57.2 ± 15.7	0.048 ± 0.004	0.002	43
*p*‐Coumaroyl‐CoA	DHPL	12.6 ± 2.5	1.315 ± 0.09	0.068	5396
pHPL	*p*‐Coumaroyl‐CoA	543.6 ± 47.4	2.62 ± 0.06	0.135	248
pHPL	Caffeoyl‐CoA	970.7 ± 219.4	0.22 ± 0.02	0.011	12
DHPL	*p*‐Coumaroyl‐CoA	866.8 ± 104.8	0.269 ± 0.01	0.014	16
pH‐optimum	7.40		Temperature optimum	24.6°C	

Values for *K*_m_ and *V*_max_ are based on *n* = 9 ± SEM, *k*_cat_ is calculated with the molecular mass of the protein (including a 6xHis‐tag) of 51.5 kDa.

### Analysis of the abundance of SgHCT transcripts in plant tissues

Three batches of total RNA were isolated from flowers, old and young leaves, old and young stems, as well as from old and young roots of *S*. *glabra* grown in‐house under ambient light conditions. The RNA preparations were subsequently purified, and 250 ng of each was converted into cDNA. Dilutions of these cDNAs were used as templates for amplification of partial sequences of SgHCTs as well as SgActin‐1 (SgAct‐1), a putative actin from *S. glabra*, as a “housekeeping gene”. Analysis of a first PCR assay on an agarose gel showed transcription of all five SgHCTs, as well as SgAct‐1, and sequencing confirmed the correct sequence of the 180–220 bp amplicons. The number of cycles was varied in a second round of PCR to ensure linear amplification and discrimination of the intensity of the bands for all sequences. The number of cycles was set to 35 because 30 cycles were insufficient to differentiate between no and very weak expression, but 40 cycles resulted in asymptotic amplification. Expression analysis was performed as a third round of PCR. Suitable conditions were at tenfold dilutions of cDNA templates and 35 PCR cycles. As expression of SgHQT2 is overall very low under these conditions, PCRs for this sequence were based on undiluted templates and 40 cycles. All seven different tissues with three replicates each were analyzed for five SgHCTs and SgAct‐1.

**Figure 4:**
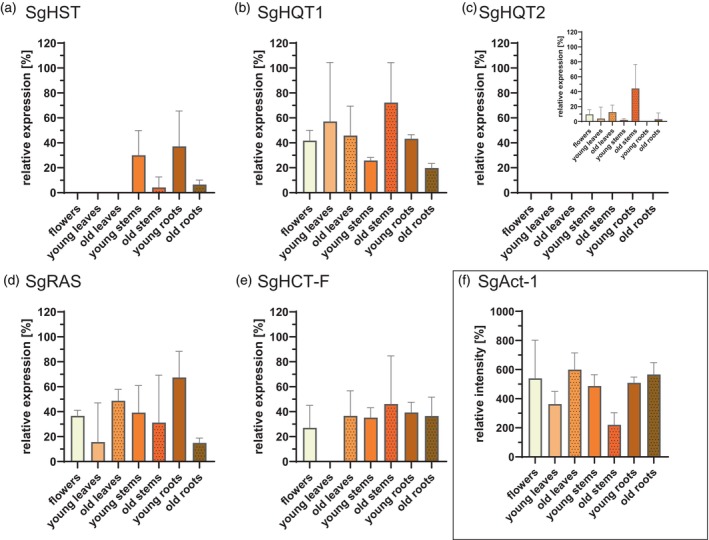
Relative expression of SgHCTs in different tissues of *Sarcandra glabra* in % (a–e), relative to the expression of SgActin‐1 in the respective tissue (f). Bars represent the mean of *n* = 3 ± SD. (a) SgHST; (b) SgHQT1; (c) SgHQT2, inset: adjusted conditions; (d) SgRAS; (e) SgHCT‐F; (f) SgAct‐1 relative intensity of the band, compared to the marker. Conditions for SgHQT2 were adjusted by using the tenfold amount of cDNA template and five additional amplification cycles, as it had an overall expression level of 0%. Values below 0.0% were set to 0.0%.

Using ImageJ (Fiji/ImageJ2), the brightness of the bands was measured (gel snippets are shown in Figure S14). The relative intensity (*I*_r_ [%]) of the bands was calculated by subtraction of the background and relativizing it to the 200 bp band from the standard DNA ladder. Thus, the intensity of the bands on different gels could be compared. SgAct‐1 was then used as a reference, assuming it was consistently expressed at equal levels in each tissue. The resulting parameter indicates the relative expression in %, related to the SgAct‐1 expression in the respective tissue, and is shown in Figure 4(f). The overall *I*_r_ of SgAct‐1 was between 220 ± 84% in old stems and 599 ± 115% in old leaves, meaning 2.2–6.0 times brighter than the 200 bp band (Figure 4f). SgHST was not expressed in flowers, old and young leaves, and corresponded to 4.3 and 6.6% of the expression of SgAct1 in old stems and old roots, respectively. The highest relative SgHST expression was found in young stems and young roots (30.0 and 37.1%, respectively) (Figure 4a). The lowest relative expression of SgHQT1 was found in old roots and young stems with 19.8 and 25.8%, respectively, and the highest relative expression was observed in old stems (72.2%) (Figure 4b). The relative expression of SgHQT1 in the other tissues ranged between 43.3% in young roots and 57.1% in young leaves (Figure 4b). SgHQT2 was overall barely to not expressed (Figure 4c). PCR conditions were therefore adjusted by using the tenfold amount of cDNA as a template and an additional five cycles of amplification. The lowest relative expression was observed in young roots, old roots, young stems and young leaves, with 0.0, 3.0, 2.2, and 3.9%, respectively (Figure 4c inset). The relative expression of SgHQT2 was increased in old stems (44.3%). The relative expression of SgRAS ranged from 14.9% in old roots to 36.6% in flowers up 67.4% in young roots (Figure 4d). Young leaves showed no relative expression of SgHCT‐F, but the relative expression ranged from 27.0% in flowers to 46.1% in old stems (Figure 4e).

## DISCUSSION

As reviewed by Zeng et al. (2021) and Chu et al. (2023), *S*. *glabra* has a broad spectrum of specialized compounds, among which 14 hydroxycinnamic acid esters (e.g., derivatives of RA, CA, caffeoylshikimic acid, caffeoyl‐3,4‐dihydroxyphenethylester) and one hydroxycinnamic acid amide (*N*‐*t*‐feruloyltyramine) were listed. In this report, we aimed at identifying genes encoding the enzymes responsible for the formation of some of these compounds. In the forerun, we therefore analyzed our own plants for hydroxycinnamic acid derivatives and could identify *p*‐coumaroyl‐ and caffeoyl‐3‐, ‐4‐, and ‐5‐*O*‐quinic acids, *p*‐coumaroyl‐ and caffeoyl‐3‐, ‐4‐, and ‐5‐*O*‐shikimic acids as well as RA and its precursors *p*‐coumaroyl‐pHPL and ‐DHPL and caf‐pHPL and an RA glycoside (Figure 1; Table S1). The main compounds among these esters were RA (up to 3.60% of the dry weight), CA (caffeoyl‐5‐*O*‐quinic acid; up to 1.99%), and caffeoyl‐5‐*O*‐shikimic acid (up to 0.31%). Only in young leaves high amounts of isorinic acid (caf‐pHPL, 6.14% of the dry mass) were detected. Quantitative analyses of RA in *S*. *glabra* reported so far range between 0.08 and 1.07% (Yin et al., 2011) and 0.25 and 1.5% (Zhu et al., 2013). In comparison, our plants contained at least twice the level of RA. Cultivation of *S*. *glabra* is important for use as a medicinal plant, e.g., in China, but since growing conditions, such as light exposure, influence the profile of phenolic substances (Xie et al., 2020, 2021), differences in total phenolic content will occur.

These results prompted us to search for hydroxycinnamoyltransferases (HCTs) with quinic, shikimic, and hydroxyphenyllactic acids as acceptors. The transcriptome database deposited in 1kP (https://db.cngb.org/onekp/) was searched with a proven sequence for RAS from *C. blumei* (syn. *Coleus scutellarioides*, *Plectranthus scutellarioides*; UniProt A0PDV5; Berger et al., 2016), and six promising scaffolds were identified, one of which was judged a wrongly built sequence (SgHCT‐B) since it contained two parts of two other scaffolds and could not be amplified by PCR. The other five scaffolds could be amplified from cDNA by PCR and/or 5′‐RACE‐PCR, and the encoded proteins were synthesized in *E. coli* as the expression host. All enzymatic activities, except for SgHCT‐F, could be identified with their main substrates as SgHST forming *p*‐coumaroyl‐/caffeoyl‐5‐*O*‐shikimic acids, SgHQT1 mainly forming *p*‐coumaroyl‐/caffeoyl‐5‐*O*‐quinic acids, SgHQT2 mainly forming *p*‐coumaroyl‐/caffeoyl‐4‐*O*‐quinic acids, and SgRAS forming *p*‐coumaroyl‐/caffeoylhydroxyphenyllactic acids. However, all enzymes showed high substrate promiscuity, accepting various other donor and acceptor substrates. Recently, Li et al. (2024) proposed a biosynthetic pathway of RA in *S*. *glabra* and identified a RAS (Genbank PP188155) with the same length as RAS identified here. The sequences differ in two base pairs (T479C, T858A), which results in amino acid sequences with two different amino acids (V160A, F286L). The SgRAS presented in this paper (Genbank PP449351) differs in four amino acid residues compared to the 1kP scaffold OSHQ‐2048693 (K45T, V160A, A213D, F286L), whereas the sequence reported by Li et al. (2024) deviates in two positions (K45T, A213D). The alterations to the scaffold deposited in the 1kP database did not impact the conserved motifs HxxxDG and DFGWG.

The catalytic activity of RAS reported by Li et al. (2024) was tested with different substrate combinations (*p*‐coumaroyl‐ and caffeoyl‐CoA, 4‐hydroxyphenyllactic and 3,4‐dihydroxyphenyllactic acids) and was found to form products from all substrate combinations, as also SgRAS reported by us. It was stated there that SgRAS mainly produces *p*‐coumaroyl‐3′,4′‐dihydroxyphenyllactic acids instead of RA, while our data suggest the preferred formation of *p*‐coumaroyl‐4′‐hydroxyphenyllactic acid. To date, the biosynthesis of 3,4‐dihydroxyphenyllactic acid is unclear. Zhou, Zou, et al. (2024) identified a CYP98A75 in *Salvia miltiorrhiza* hairy root cultures that was able to hydroxylate *p*‐coumaroyl‐4′‐hydroxyphenyllactic acid in the *meta*‐position to *p*‐coumaroyl‐3′,4′‐dihydroxyphenyllactic acid. 3,4‐Dihydroxyphenyllactic acid could be detected after incubation with *p*‐coumaroyl‐CoA, 4‐hydroxyphenyllactic acid, SmRAS, and SmCYP98A75. The hydroxylation of the 4‐hydroxyphenyllactic acid part of *p*‐coumaroyl‐/caffeoyl‐4′‐hydroxyphenyllactic acid by a CYP98 primarily acting on the *p*‐coumaroyl moiety had previously been reported by Eberle et al. (2009) for the enzyme from *C. blumei*.

We fully kinetically characterized all enzymes except SgHCT‐F and determined *K*_m_, *V*_max_ and *k*_cat_ as well as the catalytic efficiencies besides the determination of pH and temperature optima. In addition to their main substrates all enzymes showed turnover with other donor and acceptor substrates. On the side of the donor substrates cinnamoyl‐, feruloyl‐ and sinapoyl‐CoA were utilized besides *p*‐coumaroyl‐ and caffeoyl‐CoA as main substrates. Similar promiscuity is common among HCTs and has also been reported for, e.g., HST from *Nicotiana tabacum* (Hoffmann et al., 2003), HCTs from *Cichorium intybus* (Legrand et al., 2016), RAS from *Lavandula angustifolia* (Landmann et al., 2011), and RAS and HST from *C. blumei* (Sander & Petersen, 2011). Catalysis resulted in ester as well as amide formation.

The by far most effective BAHD from *S*. *glabra* is SgHST (Table 1; Figure 3a,b) with a catalytic efficiency around 400 000 L sec^−1^ mol^−1^ using *p*‐coumaroyl‐CoA and shikimic acid as substrates. The affinities towards *p*‐coumaroyl‐ and caffeoyl‐CoA are comparable, but *V*_max_ is about five times higher for *p*‐coumaroyl‐CoA. The affinity for shikimic acid is also dramatically higher with *p*‐coumaroyl‐CoA compared to caffeoyl‐CoA. Other substrates used as acceptor substrates are quinic acid (resulting in a decidedly lower catalytic efficiency; Table 1) and 3‐ and 5‐hydroxyanthranilic acid [presumably leading to the formation of amides (Ernst et al., 2022)] as well as 3‐hydroxybenzoic, 2,3‐dihydroxybenzoic, 2,5‐dihydroxybenzoic (gentisic), 3,4‐dihydroxybenzoic (protocatechuic), and 3‐aminobenzoic acids (Figure S5). These acceptors are similar in having a hydroxyl group in the *meta*‐position, except for 3‐ and 5‐hydroxyanthranilic acids, which are acylated in the *ortho*‐positional amino group. This reflects the formation of *p*‐coumaroyl‐5‐*O*‐shikimic acid with the acyl attachment in *meta*‐position of the carboxyl group. Caffeoyl‐ and *p*‐coumaroylshikimic acid can be detected in extracts from *S*. *glabra* plants at rather low levels (Figure 1; Table S1). Since these esters are considered transit products on the way towards products containing caffeoyl, feruloyl, and sinapoyl moieties, a high throughput can be coupled to low detectable product levels. The same is true for the moderate transcript levels found for SgHST (Figure 4a), which could be overcome by the high catalytic efficiencies of the enzyme. Hydroxycinnamoyl amides with 3‐ and 5‐hydroxyanthranilic acids have not been detected in *S*. *glabra* in our own investigations as well as those from Zhang et al. (2019) and Zeng et al. (2021). It is, however, known for HSTs from other plant species that hydroxyanthranilic acids are readily accepted by HSTs, although the corresponding hydroxyanthranilic amides are not accumulated in these plants (Ernst et al., 2022; Eudes et al., 2016; Landmann et al., 2011; Sander & Petersen, 2011). Thus, the acceptance of hydroxyanthranilic acids in addition to shikimic acid seems to be an intrinsic property of HST.

Regarding the catalytic efficiency, SgHQT1 is the second most efficient BAHD HCT in *S*. *glabra* (Table 2; Figure 3c,d). Interestingly, this enzyme has about twofold higher affinity towards caffeoyl‐CoA compared to *p*‐coumaroyl‐CoA (Table 2). The affinity for quinic acid is in the same range irrespective of the used donor substrate (*p*‐coumaroyl‐ or caffeoyl‐CoA; Table 2). SgHQT1 also shows low conversion with shikimic acid (Figure 2e). Despite the low affinity towards shikimic acid, three positional isomers of the resulting caffeoyl‐ and *p*‐coumaroylshikimic acids were detected (Figure S6). Prolonged incubation may result in the acylation of these isomers, leading to the formation of a dicaffeoylshikimic acid derivative, which was not fully chemically characterized and was not described in *S*. *glabra* extracts (Figure S7m). Similar observations were not made for incubations with quinic acid, nor in the assays with SgHST or SgHQT2. Dicaffeoylquinic acids have been found in *Coffea* spec., *Solanum lycopersicum*, and *Cynara cardunculus* and are under intensive investigation (Clifford et al., 2017; Moglia et al., 2014, 2016). The further acceptance of 2,3‐dihydroxybenzoic, 2,4‐dihydroxybenzoic, 2,5‐dihydroxybenzoic, and 3,4‐dihydroxybenzoic acids shows a relaxed specificity of SgHQT1 (Figure S7).

A second HQT was discovered as SgHQT2. The stereospecific formation of the precursor of cryptochlorogenic acid (cCA, caffeoyl‐4‐*O*‐quinic acid) was observed with *p*‐coumaroyl‐CoA, the best accepted donor substrate (Table 3; Figure 3e,f), but also with caffeoyl‐CoA. To our knowledge, this is the first report of an enzyme that forms cCA specifically. Most HQTs catalyze the formation of *p*‐coumaroyl‐5‐*O*‐quinic acid, yet, after treatment of a bamboo suspension culture with a histone deacetylase inhibitor, the formation of *p*‐coumaroyl‐3‐*O*‐quinic and feruloyl‐3‐*O*‐quinic acids was observed, which are derivatives of neochlorogenic acid (nCA, caffeoyl‐3‐*O*‐quinic acid) (Nomura et al., 2022). There are reports about a spontaneous migration of hydroxycinnamoyl groups between the hydroxyl groups in position 3, 4, and 5 of quinic acid (Xie et al., 2011), especially under alkaline conditions and upon longer incubation times. We assume, however, that in our case, *p*‐coumaroyl‐ and caffeoyl‐4‐*O*‐quinic acid, respectively, are the primary enzymatically formed products due to the pH of 7.5 in the reaction mixture and the comparably short incubation time (15 min). Further experiments to ensure that the observed products had not been formed by migration of hydroxycinnamoyl units were undertaken. For this, chlorogenic acid and caffeoyl‐5‐*O*‐shikimic acid were incubated in buffers of pH 6.0, 7.0, and 8.0 for 0, 0.5, 2 and 24 h. HPLC chromatograms (Figure S15a–c) show the isomeric transformation of caffeoyl‐5‐*O*‐shikimic acid over time, which is enhanced by a more alkaline pH. At pH 6.0, the emergence of two additional peaks (caffeoyl‐3‐*O*‐shikimic acid and caffeoyl‐4‐*O*‐shikimic acid) can be observed after 24 h. The formation of the same substances is enhanced at pH 7.0, and small peaks become visible after 0.5 h. More alkaline conditions (pH 8.0) accelerate the isomeric transformation so that peaks become clearly visible after 0.5 h. At pH 8.0, a strong decrease of caffeoyl‐5‐*O*‐shikimic acid was observed. Both additional peaks (caffeoyl‐3‐*O*‐shikimic acid and caffeoyl‐4‐*O*‐shikimic acid) seem to grow equally. Acyl migration for chlorogenic acid (CA) is shown by the formation of two additional peaks (nCA and cCA) over time (Figure S15d–f). An increase in pH leads to faster emergence of the two other isomers. At pH 6.0, only after 24 h, a small peak, representing cCA was observed. Whereas at pH 7.0, the emergence of cCA was seen after 0.5 to 2 h, and a peak of nCA was visible after 24 h. The process is accelerated at pH 8.0, where peaks representing both substances become visible after 0.5 to 2 h. After incubation for 24 h at pH 8.0, the initial concentration of CA is thus reduced. The acyl migration overall was, however, in a considerably lower range than in the above‐mentioned enzyme assays. We could thus conclude that a formation of different regioisomers is a genuine property of SgHQT2. SgHQT2 also accepted some benzoic acid derivatives with hydroxyl groups in *para* position, namely 2,4‐dihydroxybenzoic and 4‐hydroxybenzoic acids. Shikimic acid was taken as well, and the product with caffeoyl‐CoA as donor substrate was determined as caffeoyl‐4‐*O*‐shikimic acid using an authentic commercially available standard (see Figure S8; Table S10). Thus, this enzyme seems to have a strict stereoselectivity. The highest levels of CA (1.99% of the dry weight) could be detected in flowers, while cCA was present in a low concentration of up to 0.11% in the same tissue (Figure 1; Table S1).

Transcript abundance of SgHQT1 is rather high (Figure 4b), but the catalytic efficiency of the enzyme is moderate, which could explain the strong expression despite the comparably low levels of the resulting natural products in most tissues. SgHQT2 is generally expressed only to a very low level in all tissues (Figure 4c), and the activity is rather low, which could explain the very low amounts of cCA.

RA and its less hydroxylated precursors are formed by SgRAS (Table 4; Figure 2g,h). *p*‐Coumaroyl‐CoA is accepted with a higher affinity compared to caffeoyl‐CoA and results in the highest catalytic efficiencies with pHPL as well as DHPL as acceptor substrates (Table 4). As already reported for RAS from *C. blumei* (Sander & Petersen, 2011) or *L. angustifolia* (Landmann et al., 2011), d‐phenylalanine, d‐tyrosine, and d‐DOPA are also accepted for product formation. SgRAS did not accept l‐amino acids, but a hydroxycinnamoyl‐coenzyme A:l‐3,4‐dihydroxyphenylalanine hydroxycinnamoyltransferase (TpHDT1) from *Trifolium pratense* was able to acylate several l‐amino acids (Sullivan & Knollenberg, 2021). TpHDT1 belongs to clade 5c, which also contains SgRAS and RAS from *Phacelia campanularia*, suggesting distant relationships. RA and its precursors have been isolated from *S*. *glabra* tissues at rather high levels with 6.14% of the dry weight of caffeoyl‐4′‐hydroxyphenyllactic acid in young leaves and 1.01–3.61% of the dry weight of RA in all tissues, which can be explained by the overall high expression of SgRAS in all tissues (Figure 4d). Compared to other RAS enzymes, e.g., from Lamiaceae, SgRAS has a rather low catalytic efficiency. This has also been reported for RAS from *Phacelia campanularia* (PcRAS), which is placed in clade 5c in phylogenetic analyses, close to spermidine hydroxycinnamoyltransferases (Levsh et al., 2019) and in the same sub‐branch as SgRAS. Levsh et al. (2019) suggested that PcRAS has evolved from a different ancestral enzyme, probably a spermidine hydroxycinnamoyltransferase, but has a similar overall structure despite the rather low similarity with respect to the amino acid sequence. Presumably, this different evolutionary origin resulted in lower activities as also observed for SgRAS. In contrast, Zhou, Feng, et al. (2024) expressed and tested four HCTs from *Mentha longifolia* with RAS activity, three of them being placed in the Lamiaceae RAS subclade in the phylogenetic tree (Figure S3). MlAT1, instead, is placed in proximity to HST from *Plecthrantus scutellarioides* but accepts 4‐hydroxyphenyllactic acid. In conclusion, the biosynthesis of RA has presumably arisen several times independently during evolution and might have given plants an advantage with regard to pathogen and herbivore deterrence and UV protection.

SgHCT‐F was found to be expressed in all tissues with the highest levels in young roots and old stems (Figure 4e). Heterologous expression in *E. coli* and protein purification have been successful (Figure S4). Although a multitude of substrates have been tested in enzyme assays (Table S8), up to now no suitable substrates could be identified. The phylogenetic tree (Figure S3) places SgHCT‐F in a sub‐branch containing benzoyl‐CoA:benzyl alcohol benzoyltransferases (BBT) forming benzyl benzoate, a slightly fragrant compound found in several plant species. The presence of glucosylated benzylbenzoic acid in *S*. *glabra* has previously been described by Wu et al. (2012), and the presence of a BBT‐like enzyme in *S*. *glabra* could therefore be expected. High identities and similarities with SgHCT‐F on an amino acid basis were returned for numerous hypothetical BBTs; however, one already enzymatically characterized enzyme among this output, AAT3 from *Cucumis melo* (Cucurbitaceae), showed the highest catalytic activities with acetyl‐CoA and benzyl alcohol (El‐Sharkawy et al., 2005).

In conclusion, we could isolate four coding sequences for enzymes involved in the formation of hydroxycinnamic acid esters detected in our plant material, namely *p*‐coumaroyl‐/caffeoylshikimic, *p*‐coumaroyl‐/caffeoylquinic and *p*‐coumaroyl‐/caffeoyl‐4′‐hydroxyphenyllactic and *p*‐coumaroyl‐/caffeoyl‐3′,4′‐dihydroxyphenyllactic acids. Another enzyme (SgHCT‐F), not yet characterized, could be involved in the formation of benzoyl esters. SgHST, SgHQT1, SgHQT2, and SgRAS were fully kinetically characterized, and the genes were shown to be differentially expressed in various tissues of *S*. *glabra*.

## EXPERIMENTAL PROCEDURES

### Plant material

*Sarcandra glabra* cuttings (IPEN‐No. JP‐0‐DUSS‐4034) were received from the Botanical Garden of the Heinrich‐Heine‐Universität Düsseldorf and cultivated in‐house at 20 ± 3°C in commercial potting soil under ambient light conditions. The seed material of *S. glabra* was originally attained in 1977 from Prof. Hurusava, Okinawa, Japan. Plant material for extraction of hydroxycinnamic acid derivatives was collected in winter 2023/2024, whereas tissues for expression analysis were harvested in spring 2024. Different plant parts were considered for analysis: flowers, old and young leaves, old and young stems, as well as old and young roots. Flowers were collected during blossoming, taking the complete inflorescence, including stamen, pistil, and pedicel. Old leaves were older than 3 months, with a length of 5–10 cm and dark green, whereas young leaves were approximately 1–2 months old, small, and light green. Old stem tissue was yellow‐green and collected from the adult plants, whereas young stems were harvested when the internodes were reddish and flexible. Roots were also taken from the adult plants. The first 2 cm of the root tips were used as young roots, while thick and hard parts, at least 5 cm from the root tip, were considered as old roots. Tissue samples were harvested, frozen in liquid nitrogen, stored at −80°C and lyophilized for 48 h.

### Extraction of hydroxycinnamic acid derivatives

For phytochemical investigation of *S*. *glabra*, freeze‐dried plant parts were ground, and three samples of approximately 20 mg were taken. After the addition of 1 ml 70% aqueous ethanol, the reaction tubes were mixed vigorously for 10 sec. For cell disruption, the reaction tubes were sonicated twice for 10 min at 70°C with intense mixing for 10 sec in between. The ethanolic solution was collected after centrifugation at 900 ***g*** for 10 min. The cell pellet was again extracted with 1 ml 70% ethanol, mixed vigorously, and spun down. The ethanol phases were combined and stored at −80°C. Samples for HPLC and LC–MS were diluted 1:10 with 30% methanol acidified with 0.01% *ortho*‐phosphoric acid and centrifuged for 10 min at 17 000 ***g*.** Diluted extracts were analyzed by HPLC, and the content of 17 compounds was calculated with the help of authentic standards (25–100 μm; Tables S2, S16 and S17). Furthermore, the presence of these substances was verified via LC–MS (Table S3). Exemplary HPLC chromatograms are shown in Figure S16. The mean values, shown in Figure 1 and Table S1, are based on three replicates and are displayed in % of the cell dry weight.

### Synthesis of coenzyme A thioesters

Coenzyme A thioesters of various (hydroxy)cinnamic acids were synthesized and quantified essentially according to Stöckigt and Zenk (1975). In brief, the respective (hydroxy)cinnamic acid was converted to an *N*‐hydroxysuccinimide ester, and the *N*‐hydroxysuccinimide exchanged with coenzyme A. Purification was performed on Chromabond C18 ec columns as described by Beuerle and Pichersky (2002). Acetone in the reaction assays was removed in a vacuum centrifuge, and the residual aqueous volume was adjusted to 4% ammonium acetate, and applied to Chromabond C18 ec columns (6 ml; Macherey‐Nagel, Düren, Germany). The columns were washed with 12× 2 ml 4% ammonium acetate, and the coenzyme A esters, eluted with 12 × 2 mL distilled water. UV–Vis spectra were taken from the elution fractions, and fractions showing peaks at the appropriate wavelengths (see Stöckigt & Zenk, 1975) were collected and concentrated. The concentrations were set by using the ε from the stated literature, and aliquots were stored at −20°C until further use.

### DNA and RNA isolation and synthesis of cDNA

Genomic DNA was isolated from leaves as described by Rogers and Bendich (1985). The protocols of Pearson et al. (2006) or Chomczynski and Sacchi (1987) were followed for the isolation of total RNA from young leaves. cDNA was synthesized with the RevertAid First Strand cDNA Synthesis kit (Thermo Scientific, Waltham, MA, USA) according to the manufacturer's protocol.

### Identification of target genes and amplification

The transcriptome of *Sarcandra glabra* (taxid: 92927, sample code: OSHQ) in the 1kP database (https://db.cngb.org/onekp/; Carpenter et al., 2019; Leebens‐Mack et al., 2019) was searched with BLASTP (using default parameters) for scaffolds with similarity to the amino acid sequence of rosmarinic acid synthase (RAS) from *C. blumei* (syn. *Coleus scutellarioides*, *Plectranthus scutellarioides*; UniProt A0PDV5; Berger et al., 2016). Hits with an *e*‐value lower than 1 × 10^−50^ were selected for further characterization.

For expression analysis, a BLASTP search with *Persea americana* actin (GU272027) in 1kP led to several scaffolds, with one of them, SgActin‐1 (SgAct‐1; OSHQ‐2000170), having an identity on the amino acid level of 99.2%. This gene was used as a “housekeeping gene” but not further analyzed.

PCR amplification of appropriate full‐length or partial sequences using cDNA was performed to verify the expression of the sequences and to obtain full‐length sequences (for primer sequences and amplification conditions, see Table S18). Genomic sequences were checked for the presence of introns using gDNA as a template instead (for PCR conditions, see Table S19). Primers were purchased from Eurofins Genomics (Ebersberg, Germany). For full‐length sequences, the restriction sites for ligation into the expression vector pET‐15b were included. To identify the missing 5′‐end of SgHQT2, 5′‐RACE‐PCR was performed using the SMARTer^®^ RACE 5′/3′ kit (Takara, Saint Germain de Laye, France). After agarose gel electrophoresis, bands at the expected size were cut out, eluted using the Nucleospin Gel Extraction and PCR Purification Kit (Macherey‐Nagel, Düren, Germany), and ligated into pDRIVE (Qiagen, Hilden, Germany) for sequence verification. The plasmids were transferred into *E. coli* EZ for amplification and subsequent plasmid isolation. Sequencing was performed by Microsynth Seqlab, Göttingen, Germany). Full‐length sequences were ligated into suitable restriction sites (*Nde*I, *Bam*HI, *Xho*I) of the expression plasmid pET‐15b. *E. coli* EZ cells were transformed with the plasmids harboring the full‐length sequences, which were sequenced again. The plasmids for expression were then transferred into the expression host cell strain *E. coli* SoluBL21 (Amsbio, Alkmaar, The Netherlands).

### Protein expression, SDS‐PAGE and Western blot analysis

*E. coli* SoluBL21 carrying the pET‐15b plasmids for expression, containing full‐length HCT sequences, were cultivated for 16 h in LB medium with 100 μg ml^−1^ ampicillin at 37°C under shaking at 220 rpm. These cultures were inoculated into TB medium containing 100 μg ml^−1^ ampicillin (for media composition see Lessard, 2013), and incubation took place at 37°C for 2 to 3 h at 220 rpm up to an optical density at 600 nm of 0.4 to 0.6 in Erlenmeyer flasks. Protein expression was induced by the addition of 1 mm isopropyl‐β‐d‐galactopyranoside, followed by incubation for 20 h at 25°C and 160 rpm. Bacteria were harvested by centrifugation for 10 min at 4°C and 10 000 ***g*.** Sedimented bacteria were frozen in liquid nitrogen and stored at −80°C for at least 2 h until they were resuspended in 4 ml 50 mm K_2_HPO_4_/KH_2_PO_4_ pH 8.0 per 1 g cell weight. The cells were ultrasonicated on ice (3 × 30 sec, 100%, 0.3 cycles) after 30 min of incubation with approximately 50 mg lysozyme. The supernatant of a 10 min centrifugation at 4°C and 10 000 ***g*** was further purified on Ni‐NTA resin (Carl Roth, Karlsruhe, Germany) via the *N*‐terminally attached 6xHis‐tag to obtain highly enriched HCT proteins. After adjusting the crude protein extract to 10 mm imidazole and 300 mm NaCl, it was added to 1 ml pre‐equilibrated Ni‐NTA resin in a disposable column and incubated on ice for 1 h. After discarding the flow‐through, the resin was washed with 10 ml wash buffer (50 mm K_2_HPO_4_/KH_2_PO_4_ pH 8.0, 10 mm imidazole, 300 mm NaCl) and eluted with 3 ml elution buffer (50 mm K_2_HPO_4_/KH_2_PO_4_ pH 8.0, 250 mm imidazole, 300 mm NaCl). Elution fractions were desalted by gel filtration through PD‐10 columns (GE Healthcare) into 0.1 m K_2_HPO_4_/KH_2_PO_4_ pH 7.0. All protein concentrations were determined according to Bradford (1976) using bovine serum albumin (1 mg ml^−1^) as a standard. Purified protein preparations were stored at −80°C. Cells harboring an empty pET‐15b were treated equally and used as an empty vector control.

SDS‐PAGE was essentially performed as described by Laemmli (1970). After SDS‐PAGE, the gel was subjected to semi‐dry Western blotting basically as specified by Mahmood and Yang (2012), but using the discontinuous buffer system described in the user protocol of the Immobilon‐P membrane (https://www.sigmaaldrich.com/deepweb/assets/sigmaaldrich/product/documents/159/578/ipvh85rug‐mk). The expressed proteins were detected with mouse anti‐6xHis‐tag monoclonal antibodies (Invitrogen, Life Technologies, Darmstadt, Germany; MA1‐21315), followed by goat anti‐mouse antibodies conjugated to alkaline phosphatase (Life Technologies; A16087) as a secondary antibody. The localization of alkaline phosphatase was visualized with nitro blue tetrazolium chloride/5‐bromo‐4‐chloro‐3‐indolyl‐phosphate following standard protocols (https://www.sysy.com/protocols/blot.php). SDS‐PAGE gels were stained with Coomassie Brilliant Blue R250. Both SDS‐PAGE gels and Western blots are displayed in Figure S4.

### Enzyme assays

The catalytic activity of heterologously synthesized proteins was tested in standard assays containing *p*‐coumaroyl‐ or caffeoyl‐CoA and suitable acceptor substrates in a total volume of 125 μl 0.1 m K_2_HPO_4_/KH_2_PO_4_ pH 7.0. The detailed composition of the enzyme assays and the specific conditions are presented in Tables S12–S15. Control reactions for testing putative acceptor substrates were performed with purified extracts from *E. coli* SoluBL21 carrying the empty vector pET‐15b. The reaction was stopped by the addition of 20 μl 6 N HCl, intense mixing, followed by storing the reaction tubes on ice. The reaction products were extracted by vigorously shaking them twice with 500 μl ethyl acetate each, short centrifugation, and collection of the upper phases. The ethyl acetate phases were combined and evaporated to dryness in a vacuum concentrator. The residue was redissolved in 100 μl of 30% or 45% (v/v) methanol (see below) acidified with 0.01% (v/v) *ortho*‐phosphoric acid, and the samples were subjected to HPLC and/or LC–MS analysis. Determination of biochemical parameters such as *K*_m_ and *V*_max_ was performed after assuring linear turnover with the chosen amount of protein at the lowest and highest concentration of the variable substrate. The concentrations of the constant substrate was about 10 times its *K*_m_, if possible. Substrate saturation curves were mostly calculated from nine replicates from one to three individual expressions (*n* = 9) and were analyzed via GraphPad Prism 10.

### HPLC and LC–MS analysis

Quantitative analysis of enzyme assays and ethanolic plant extracts was performed by HPLC, using a Hitachi Chromaster system (VWR International, Darmstadt, Germany) with isocratic elution with 30% (plant extracts; enzyme products with shikimic or quinic acid as acceptor) or 45% (all other enzyme assay products) (v/v) methanol acidified with 0.01% *ortho*‐phosphoric acid at a flow rate of 1 ml min^−1^ and 35°C on a Hypersil ODS column (250 × 4 mm; pre‐column: 20 × 4 mm; particle size 5 μm; Dr. Maisch GmbH, Ammerbuch, Germany). Peaks were detected and integrated at 312 nm for *p*‐coumaroyl and 333 nm for caffeoyl derivatives (cinnamoyl: 280 nm; feruloyl and sinapoyl: 333 nm).

For LC–MS analysis, an Agilent Technologies (Waldbronn, Germany) HPLC 1260 with a Multospher 120 RP18 column (250 × 2 mm, particle size 5 μm; CS‐Chromatographie Service, Langerwehe, Germany) was used with a solvent system of solvent A (0.1% [v/v] aqueous formic acid) and solvent B (0.1% [v/v] formic acid in acetonitrile) at a flow rate of 0.25 ml min^−1^ at 20°C. For qualitative analysis of ethanolic *S*. *glabra* extracts, the following gradient was set: 0–40 min 5% B → 100% B, 40–45 min 100% B, 45–45.10 min 100% B → 5% B, 45.10–55 min 5% B. For the verification of product formation in enzyme assays with SgHCTs, a short method was used: 0–10 min 5% B → 100% B, 10–15 min 100% B, 15.10–20 min 5% B, and a flow rate of 0.50 ml min^−1^ at 25°C. Detection was performed with a diode array detector at 190–400 nm and a micrOTOF‐Q III (Bruker Daltonics, Billerica, MA, USA) with an ESI source calibrated with 5 mm CHNaO_2_ in the negative ion mode. For identification of substances, samples of 10 μl of the ethanolic extracts, enzyme assays, or authentic standards (25–100 μm) were injected. Authentic standards, which were used for identification and quantification, were obtained from different sources (see Tables S16 and S17 for detailed information).

### Migration of hydroxycinnamoyl units in chlorogenic and caffeoylshikimic acids

Ten microliters of 625 μm CA or caffeoyl‐5‐*O*‐shikimic acid were added to 115 μl of 0.1 m KH_2_PO_4_/K_2_HPO_4_ buffer pH 6.0, 7.0, and 8.0 and incubated at 30°C. Twenty microliters 6 N HCl was added prior (0 h), after 0.5, 2 or 24 h. The assays were extracted twice with 500 μl ethyl acetate, and the ethyl acetate evaporated. The residues were redissolved in 30% methanol, acidified with 0.01% H_3_PO_4_, centrifuged, and subjected to HPLC (see above; Figure S16).

### Analysis of the abundance of SgHCT transcripts in plant tissues

For expression analysis, RNA from different tissues of *S*. *glabra* plants was isolated according to Pearson et al. (2006); the integrity of the resulting RNA was checked on 1% (m/v) agarose gels, and the concentration was determined photometrically. Only undegraded RNA with distinct 28S, 18S, and 5S rRNA bands was processed further. Digestion of gDNA contaminants with DNase I (Thermo Scientific) was followed by a second round of RNA purification using only a tenth of the volumes mentioned in the protocol of Pearson et al. (2006). Resulting RNA samples with an *A*_260_/_280_ ratio between 1.80 and 2.00 were reverse‐transcribed into cDNA using 250 ng RNA and the RevertAid^®^ cDNA synthesis kit (Thermo Scientific) in accordance with the manufacturer's instructions. All five SgHCT sequences, as well as SgAct‐1 as a “housekeeping gene”, were amplified by PCR from cDNA under identical conditions (see Table S20). Primers for SgHCTs and SgAct‐1 were designed using the PCR Primer Design Tool (https://eurofinsgenomics.eu/en/ecom/tools/pcr‐primer‐design/), excluding regions with high similarity between SgHCTs. Primers had a length of 20 to 25 bp, a melting temperature of 59–65°C, a GC content between 40 and 60%, and amplified sequences had a length of 180–220 bp. Linear amplification was checked beforehand. The PCR assays were analyzed on 1% (m/v) agarose gels containing 50 μg ethidium bromide per 100 ml. Bands were sequenced once after gel extraction, ligation into pDRIVE^®^ (Qiagen), transfer into *E. coli* EZ (Qiagen), and plasmid isolation. Pictures of the agarose gels were taken with identical camera settings, and the intensity of the bands was analyzed using ImageJ (Fiji/ImageJ2 v1.54k), as the intensity of the bands depends on the transcript abundance. After selection of the amplicon bands, the average intensity of the background of the gel was subtracted from the average intensity of the selected band. Negative values resulted when the background was slightly brighter than the selected area where a band would have been expected (e.g., due to dust or bubbles in the gel). The adjusted intensity was then multiplied with the area of the band, which resulted in the absolute intensity (*I*_a_). The *I*_a_ for the marker's 200 bp band was used as a reference, as 4.00 μl of the marker (GeneRuler DNA ladder; Thermo Scientific) were applied to every gel. The relative intensity (*I*_r_ [%]) was calculated as the ratio of the *I*_a_ of the amplicon band and the *I*_a_ of the marker's 200 bp band (in %). In the next step, the *I*_r_ of each band was related to the *I*_r_ of the SgAct‐1 band of the respective tissue und replicate, leading to *X*% *I*_r_/*I*_r_^SgAct‐1^ (*n* = 3 ± SD) (see Figure 4a–e). The *I*_r_ of SgAct‐1 in each tissue is displayed in Figure 4(f).

### Bioinformatic analyses

Sequence similarity calculations for SgHCTs (Table S5) were performed with EMBOSS Needle (www.bioinformatics.nl/cgi‐bin/emboss/needle). CLC Sequence Viewer 8 (www.qiagenbioinformatics.com) was used for alignments using default settings. Phylogenetic analyses were performed with MEGA11 (Tamura et al., 2021) using the Maximum Likelihood algorithm (default settings, 1000 bootstraps) for hydroxycinnamoyltransferase sequences collected from databanks and named in Kruse et al. (2022) and Moghe et al. (2023) (see Table S6).

### ACCESSION NUMBERS

The cDNA coding sequences of the enzymes characterized in this work were deposited in Genbank as PP449349 *Sarcandra glabra* hydroxycinnamoyl‐CoA:shikimate hydroxycinnamoyltransferase (SgHST), PP449350 *Sarcandra glabra* hydroxycinnamoyl‐CoA:quinate hydroxycinnamoyltransferase (SgHQT1), PP449351 *Sarcandra glabra* hydroxycinnamoyl‐CoA:hydroxyphenyllactate hydroxycinnamoyltransferase (SgRAS), PP449352 *Sarcandra glabra* hydroxycinnamoyl‐CoA:quinate hydroxycinnamoyltransferase (SgHQT2), and PQ336776 (SgHCT‐F).

## CONFLICT OF INTEREST

The authors declare no conflicts of interest.

## Supporting information


**Figure S1.** Structures and nomenclature of *p*‐coumaroyl/caffeoylquinic, ‐shikimic and *p*‐coumaroyl/caffeoylhydroxyphenyllactic acids.
**Figure S2.** Alignment of SgHCT amino acid sequences.
**Figure S3.** Phylogenetic tree for hydroxycinnamoyltransferase sequences.
**Figure S4.** SDS‐PAGE gels and Western blots of heterologously synthesized SgHCTs.
**Figure S5.** Extracted ion chromatograms (EIC) of enzyme assays with SgHST.
**Figure S6.** HPLC chromatograms at 333 nm of a test with SgHQT1, caffeoyl‐CoA and shikimic acid.
**Figure S7.** Extracted ion chromatograms (EIC) of enzyme assays with SgHQT1.
**Figure S8.** Extracted ion chromatograms (EIC) of enzyme assays with SgHQT2.
**Figure S9.** Extracted ion chromatograms (EIC) of enzyme assays with SgRAS.
**Figure S10.** pH‐Optimum, temperature optimum and Michaelis–Menten kinetics (*K*_m_ and *V*_max_) for SgHST.
**Figure S11.** pH‐Optimum, temperature optimum and Michaelis–Menten kinetics (*K*_m_ and *V*_max_) for SgHQT1.
**Figure S12.** pH‐Optimum, temperature optimum and Michaelis–Menten kinetics (*K*_m_ and *V*_max_) for SgHQT2.
**Figure S13.** pH‐Optimum, temperature optimum and Michaelis–Menten kinetics (*K*_m_ and *V*_max_) for SgRAS.
**Figure S14.** Agarose gels used for analyzing relative expression of SgHCTs in different tissues of *Sarcandra glabra*.
**Figure S15.** HPLC chromatograms recorded at 333 nm showing the acyl migration for chlorogenic acid and caffeoyl‐5‐*O*‐shikimic acid at three pH values over time.
**Figure S16.** Phytochemical analysis of *Sarcandra glabra*: Exemplary LC–MS chromatograms at 333 nm of replicate 1.
**Table S1.** Hydroxycinnamic acid derivatives in plant parts of *Sarcandra glabra*.
**Table S2.** Compounds in *Sarcandra glabra* identified by HPLC.
**Table S3.** Compounds in *Sarcandra glabra* identified by LC–MS analysis.
**Table S4.** BLASTP results in 1kP for *Sarcandra glabra*.
**Table S5.** Pairwise comparison (EMBOSS Needle) of amino acid sequences of SgHCTs.
**Table S6.** Abbreviations for species names used in the phylogenetic tree.
**Table S7.** LC–MS analysis of enzyme assays with SgHST.
**Table S8.** Tested putative acceptor substrates for all SgHCTs.
**Table S9.** LC–MS analysis of enzyme assays with SgHQT1.
**Table S10.** LC–MS analysis of enzyme assays with SgHQT2.
**Table S11.** LC–MS analysis of enzyme assays with SgRAS.
**Table S12.** Assay compositions in tests with SgHST.
**Table S13.** Assay compositions in tests with SgHQT1.
**Table S14.** Assay compositions in tests with SgHQT2.
**Table S15.** Assay compositions in tests with SgRAS.
**Table S16.** List of authentic standards.
**Table S17.** LC–MS analysis of authentic standards.
**Table S18.** Primer sequences and PCR conditions for the amplification of hydroxycinnamoyltransferase sequences in *Sarcandra glabra*.
**Table S19.** Primer sequences for gDNA analysis of SgHCTs and PCR conditions.
**Table S20.** Primer sequences and PCR conditions for expression analysis.

## Data Availability

The data that support the findings of this study are available on request from the corresponding author. The data are not publicly available due to privacy or ethical restrictions.
