# Supplementary material for: Phenolic metabolism in Sarcandra glabra is mediated by distinct BAHD hydroxycinnamoyltransferases
Source: Plant J. 2025 Mar 3;121(5):e70035. doi: 10.1111/tpj.70035 (PMC11875395; doi:10.1111/tpj.70035)
Supplement: Supplementary file 1 — Figure S1. Structures and nomenclature of p‐coumaroyl/caffeoylquinic, ‐shikimic and p‐coumaroyl/caffeoylhydroxyphenyllactic acids. Figure S2. Alignment of SgHCT amino acid sequences. Figure S3. Phylogenetic tree for hydroxycinnamoyltransferase sequences. Figure S4. SDS‐PAGE gels and Western blots of heterologously synthesized SgHCTs. Figure S5. Extracted ion chromatograms (EIC) of enzyme assays with SgHST. Figure S6. HPLC chromatograms at 333 nm of a test with SgHQT1, caffeoyl‐CoA and shikimic acid. Figure S7. Extracted ion chromatograms (EIC) of enzyme assays with SgHQT1. Figure S8. Extracted ion chromatograms (EIC) of enzyme assays with SgHQT2. Figure S9. Extracted ion chromatograms (EIC) of enzyme assays with SgRAS. Figure S10. pH‐Optimum, temperature optimum and Michaelis–Menten kinetics (K m and V max) for SgHST. Figure S11. pH‐Optimum, temperature optimum and Michaelis–Menten kinetics (K m and V max) for SgHQT1. Figure S12. pH‐Optimum, temperature optimum and Michaelis–Menten kinetics (K m and V max) for SgHQT2. Figure S13. pH‐Optimum, temperature optimum and Michaelis–Menten kinetics (K m and V max) for SgRAS. Figure S14. Agarose gels used for analyzing relative expression of SgHCTs in different tissues of Sarcandra glabra. Figure S15. HPLC chromatograms recorded at 333 nm showing the acyl migration for chlorogenic acid and caffeoyl‐5‐O‐shikimic acid at three pH values over time. Figure S16. Phytochemical analysis of Sarcandra glabra: Exemplary LC–MS chromatograms at 333 nm of replicate 1. Table S1. Hydroxycinnamic acid derivatives in plant parts of Sarcandra glabra. Table S2. Compounds in Sarcandra glabra identified by HPLC. Table S3. Compounds in Sarcandra glabra identified by LC–MS analysis. Table S4. BLASTP results in 1kP for Sarcandra glabra. Table S5. Pairwise comparison (EMBOSS Needle) of amino acid sequences of SgHCTs. Table S6. Abbreviations for species names used in the phylogenetic tree. Table S7. LC–MS analysis of enzyme assays with SgHST. Table S [file TPJ-121-0-s001.pdf]

Supporting Information for

Phenolic metabolism in *Sarcandra glabra* is mediated by distinct BAHD hydroxycinnamoyltransferases

Paul Bömeke and Maike Petersen

Institut für Pharmazeutische Biologie und Biotechnologie, Philipps-Universität Marburg, Robert-Koch-Str. 4, 35037 Marburg, Germany

Supplementary Figures

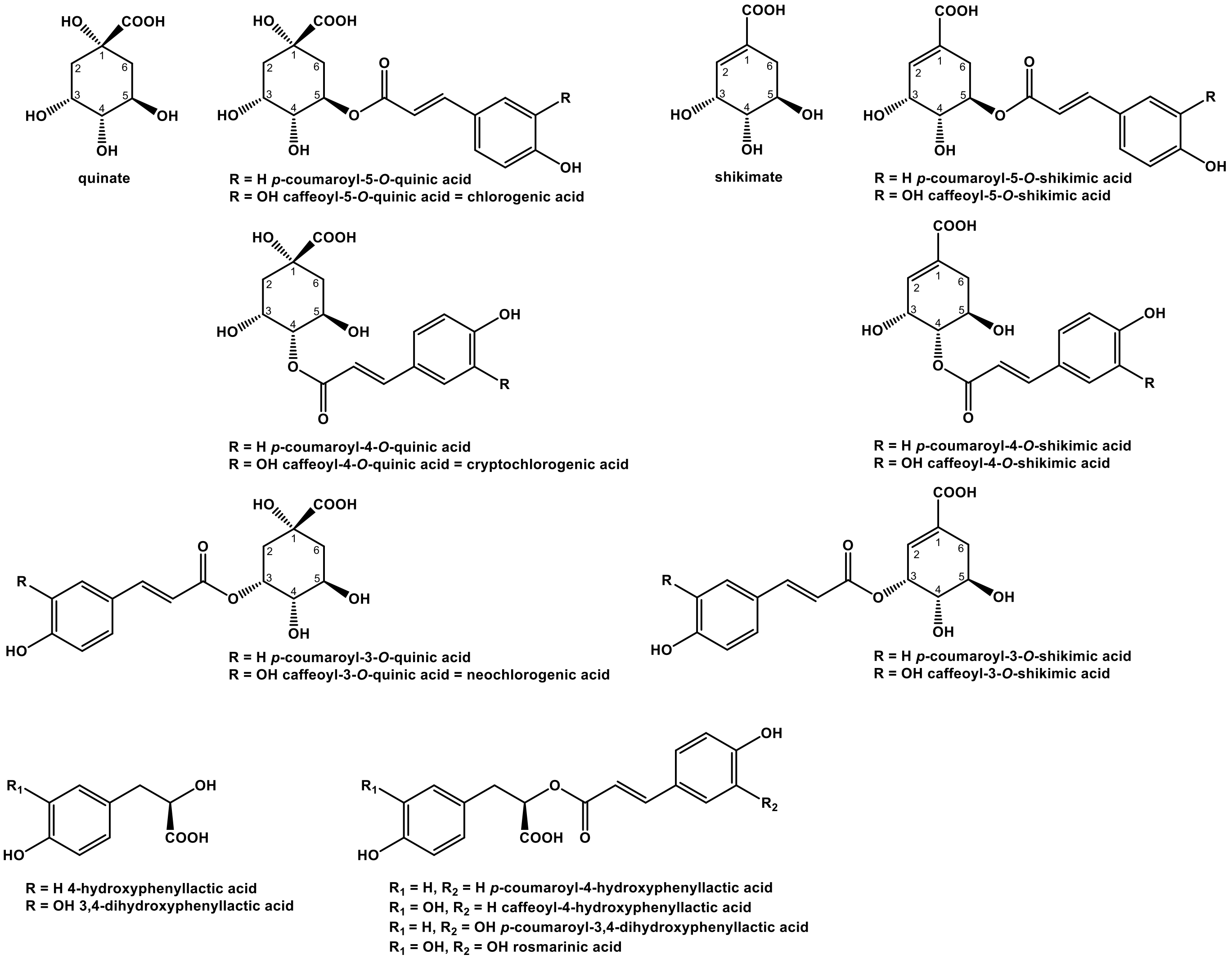

Figure S1. Structures and nomenclature of *p*-coumaroyl/caffeoylquinic, -shikimic and *p*-coumaroyl/caffeoylhydroxyphenyllactic acids used in this report.

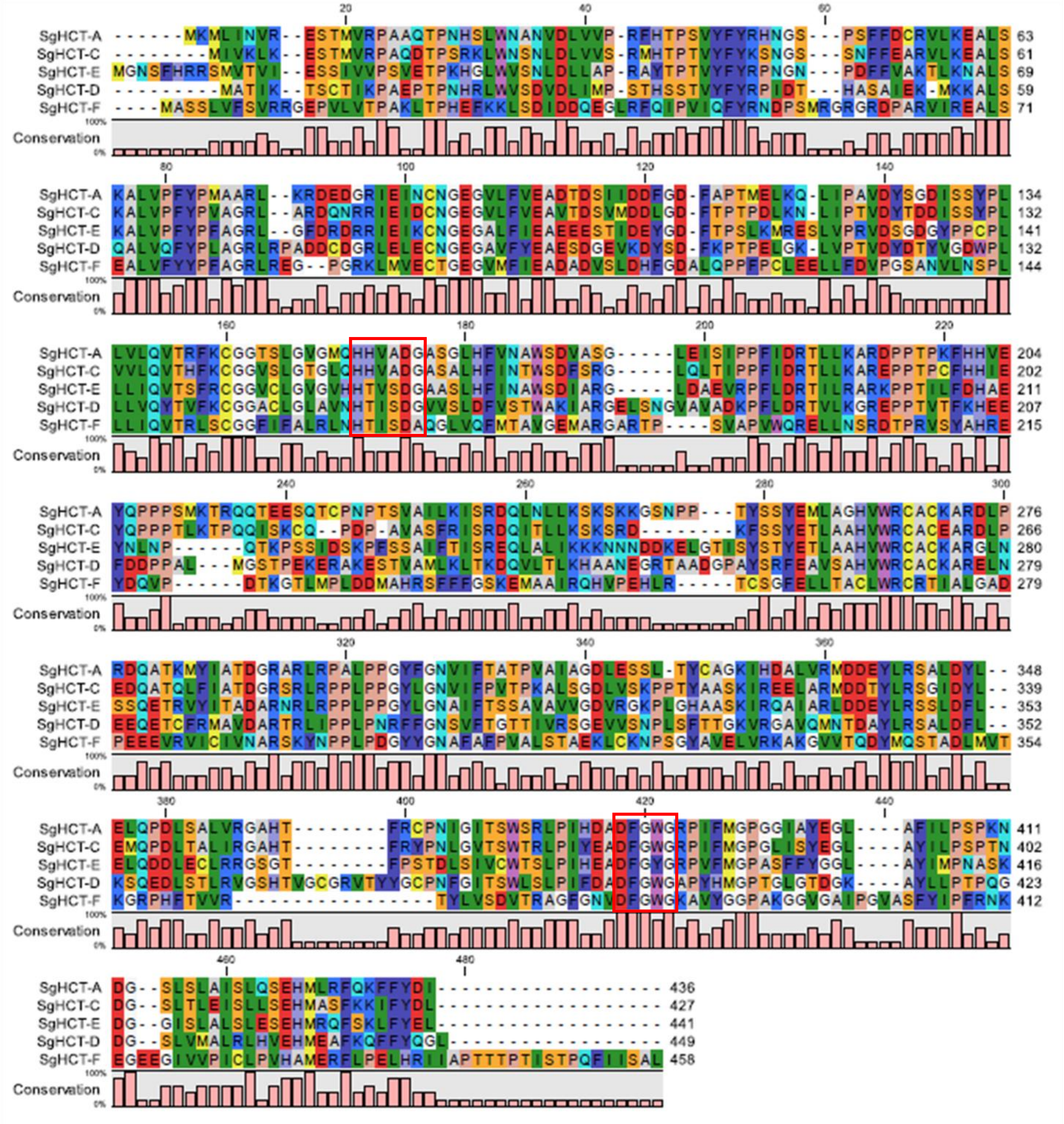

Figure S2. Alignment of SgHCT amino acid sequences of enzymes characterized in this report (CLC sequence viewer 8, default settings). The generally accepted conserved sequence motifs HxxxDG and DFGWG are marked by red boxes.

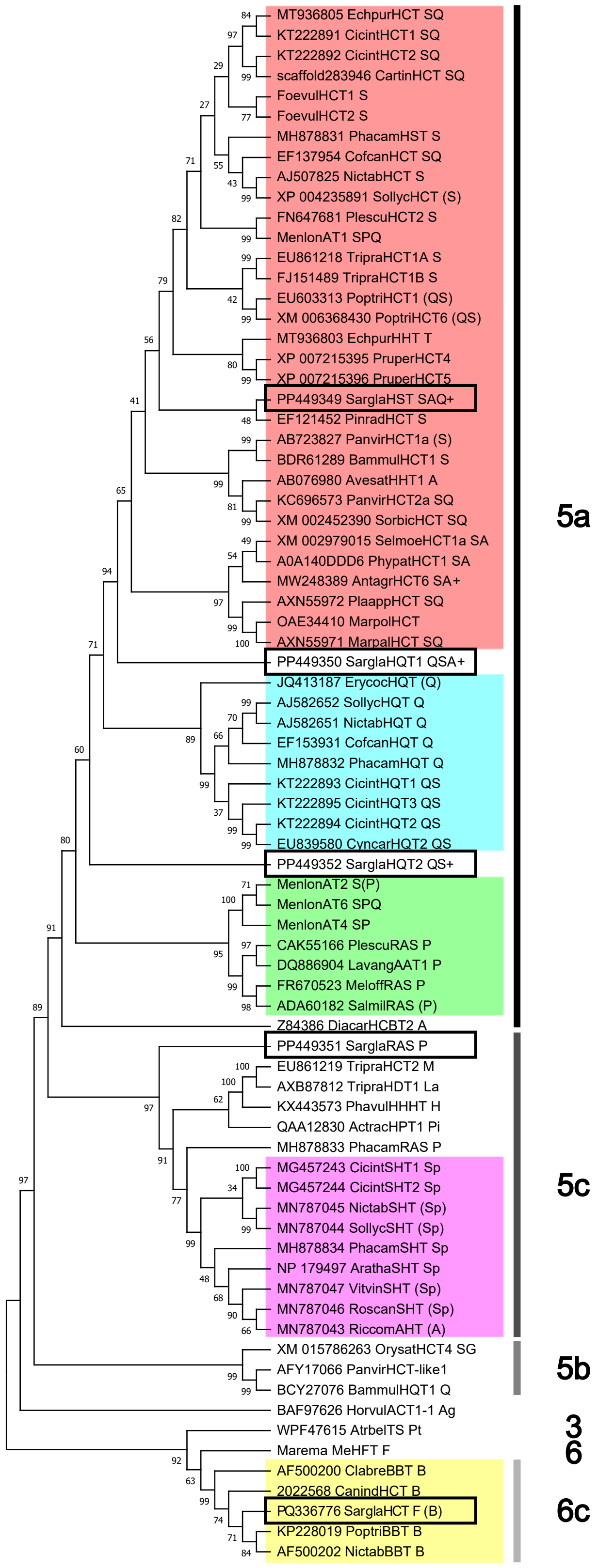

Figure S3. Phylogenetic tree for hydroxycinnamoyltransferase sequences calculated by MEGA11 (Tamura *et al.*, 2021) using the Maximum Likelihood algorithm with default settings and 1000 bootstrap replicates. Bars and numbers represent clades defined by Kruse *et al.* (2022) and Moghe *et al.* (2023). HCTs from *Sarcandra glabra* are marked with black boxes.

Colors represent the main substrates: red – shikimic acid, blue – quinic acid, green – phenyllactic acid derivatives, purple – spermine/spermidine, yellow – benzyl alcohol. Accession numbers and abbreviations for species can be taken from Table S6. (Putatively) accepted substrates are abbreviated as follows: A (anthranilic acid, hydroxyanthranilic acid), Ag (agmatine), B (benzyl alcohol), F (fatty acid derivatives), G (glycerol), La (L-amino acids) M (malic acid), P (4-hydroxyphenyllactic acid, 3,4-dihydroxyphenyllactic acid), Pi (piscidic acid), Pt (pseudotropine), Q (quinic acid), S (shikimic acid), Sp (spermidine, spermine), + (and additional substrates).

	SDS-PAGE stained with Coomassie Brilliant Blue R250	Western blot with NBT/BCIP-detection of anti-6xHis-tag
SgHST	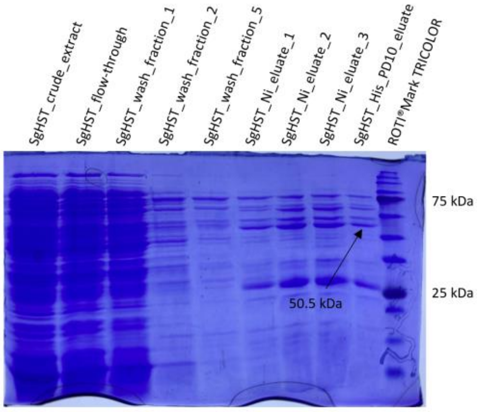	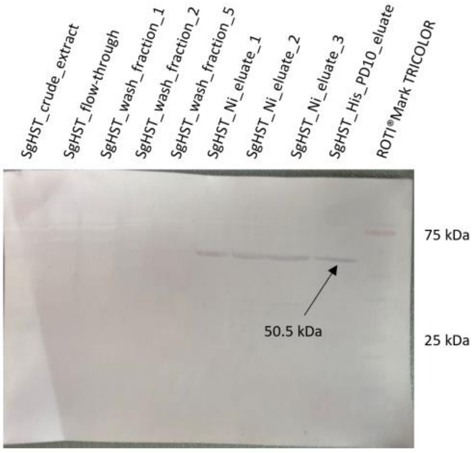
SgHQT1	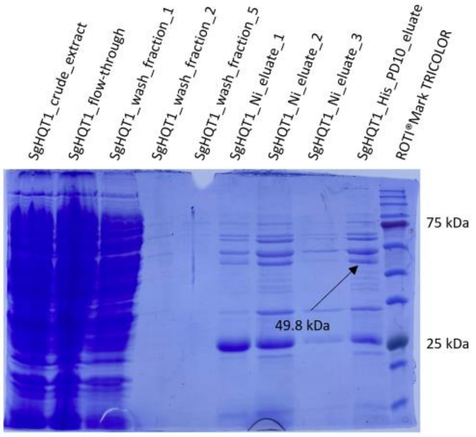	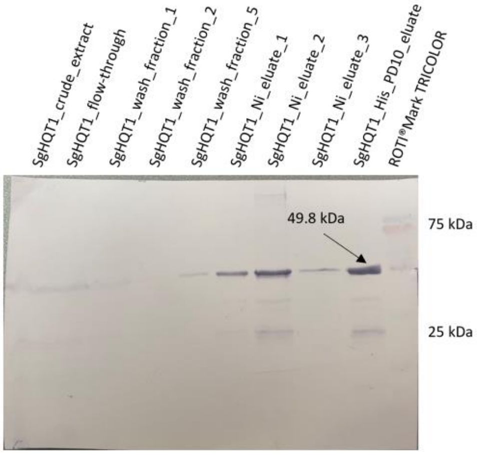
SgHQT2	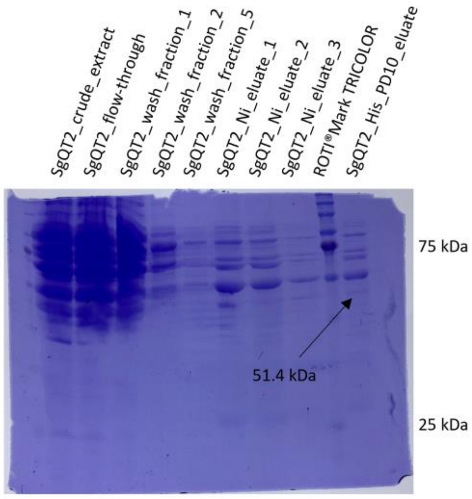	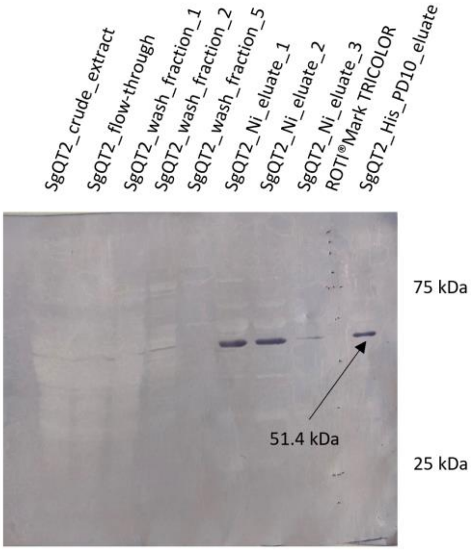

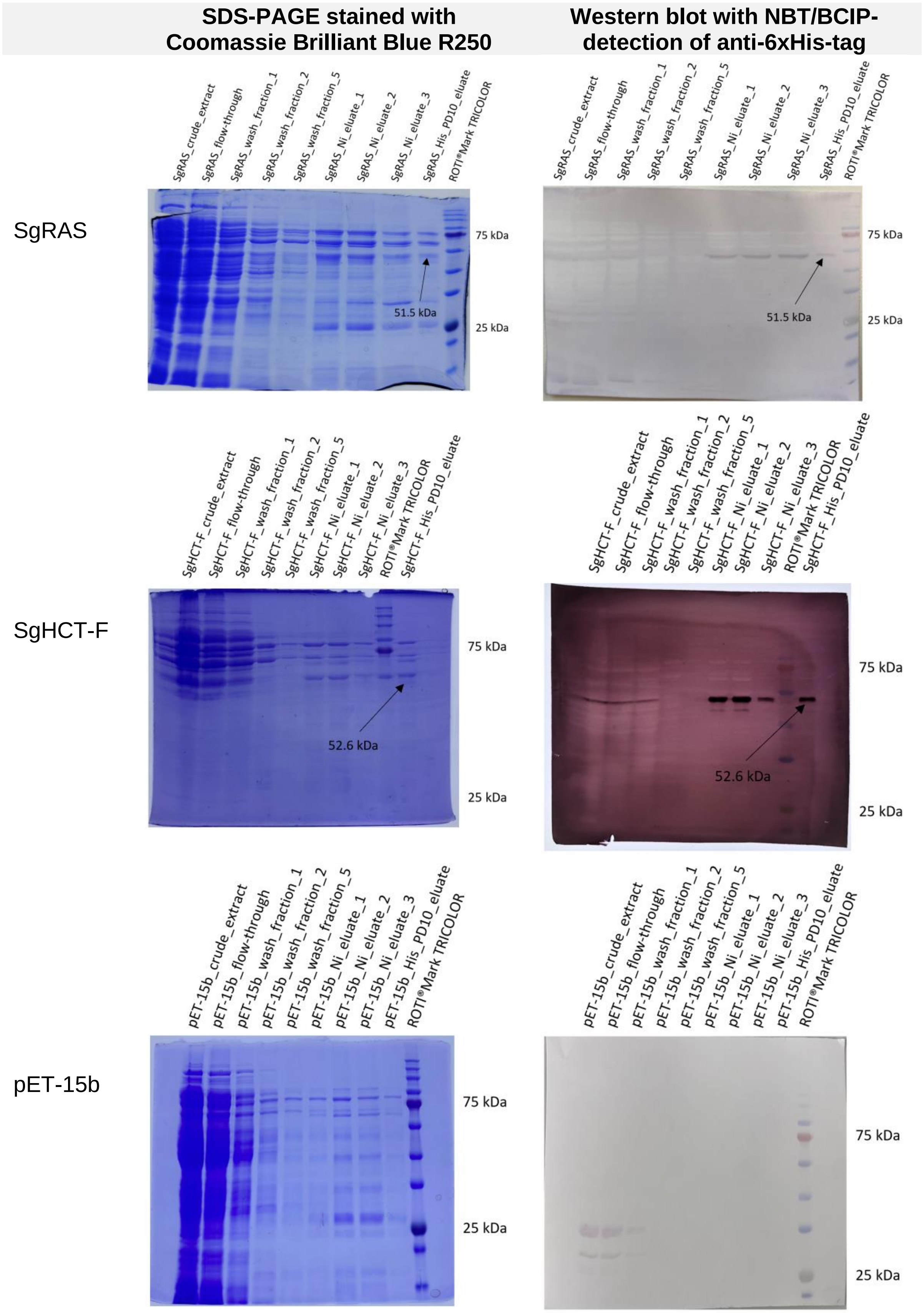

Figure S4. Heterologously synthesized SgHCTs were purified by nickel chelate chromatography, desalted on PD-10 columns and analyzed by SDS-PAGE and Western blots.

SDS-gels were stained with Coomassie Brilliant Blue, Western blots were treated with mouse anti-6xHis antibodies and goat anti-mouse antibodies coupled with alkaline phosphatase. Staining was mediated with help of nitro blue tetrazolium chloride (NBT) and 5-bromo-4-chloro-3-indolyl-phosphate (BCIP). A gel and a blot of purification steps from *Escherichia coli*, harboring empty pET-15b are shown as control.

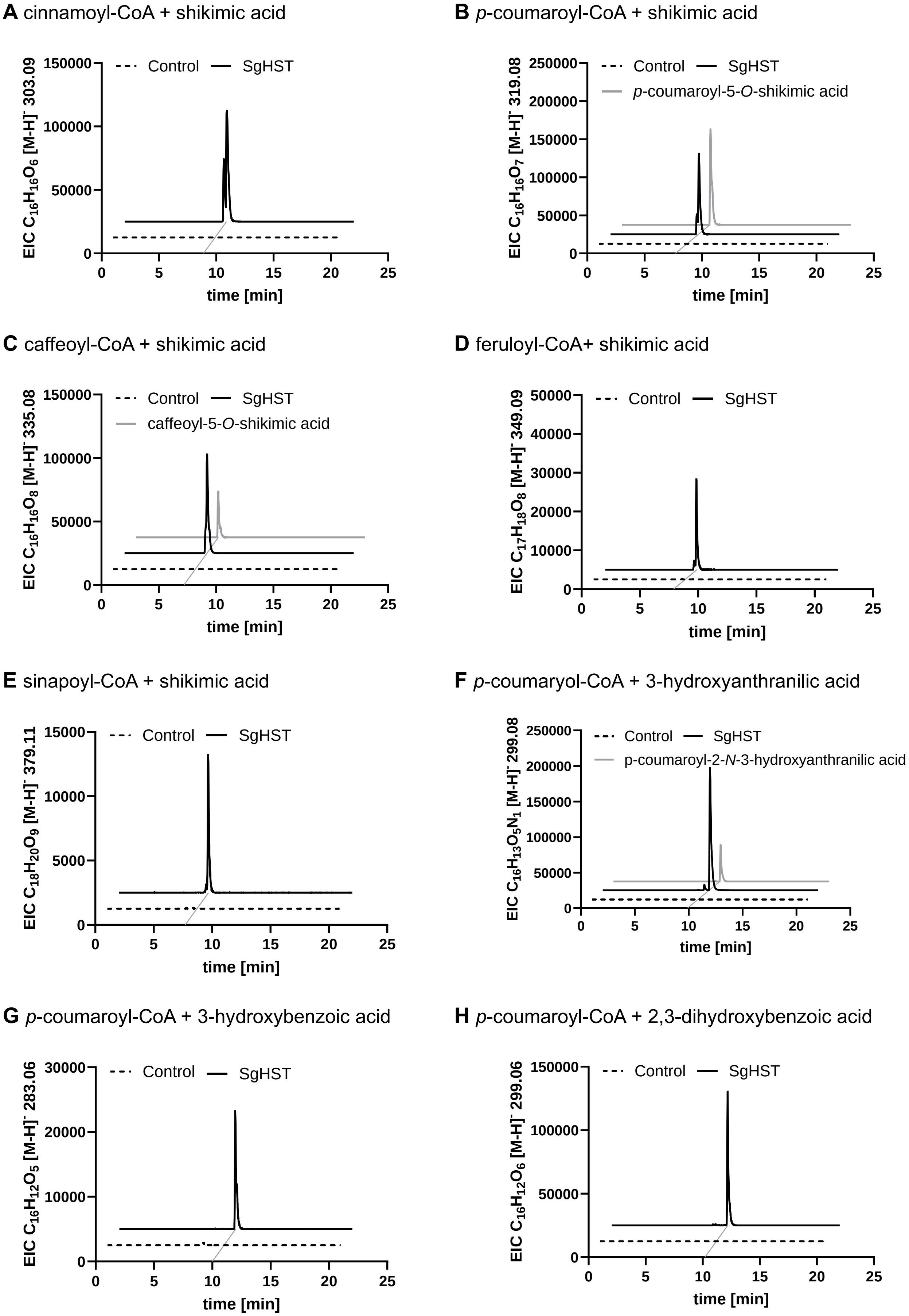
A cinnamoyl-CoA + shikimic acid
EIC C16H16O6 [M-H]- 303.09
Control
SgHST
time [min]
B p-coumaroyl-CoA + shikimic acid
EIC C16H16O7 [M-H]- 319.08
Control
SgHST
p-coumaroyl-5-O-shikimic acid
time [min]
C caffeoyl-CoA + shikimic acid
EIC C16H16O8 [M-H]- 335.08
Control
SgHST
caffeoyl-5-O-shikimic acid
time [min]
D feruloyl-CoA+ shikimic acid
EIC C17H18O8 [M-H]- 349.09
Control
SgHST
time [min]
E sinapoyl-CoA + shikimic acid
EIC C18H20O9 [M-H]- 379.11
Control
SgHST
time [min]
F p-coumaryol-CoA + 3-hydroxyanthranilic acid
EIC C16H13O5N1 [M-H]- 299.08
Control
SgHST
p-coumaroyl-2-N-3-hydroxyanthranilic acid
time [min]
G p-coumaroyl-CoA + 3-hydroxybenzoic acid
EIC C16H12O5 [M-H]- 283.06
Control
SgHST
time [min]
H p-coumaroyl-CoA + 2,3-dihydroxybenzoic acid
EIC C16H12O6 [M-H]- 299.06
Control
SgHST
time [min]

I *p*-coumaroyl-CoA + 2,5-dihydroxybenzoic acid

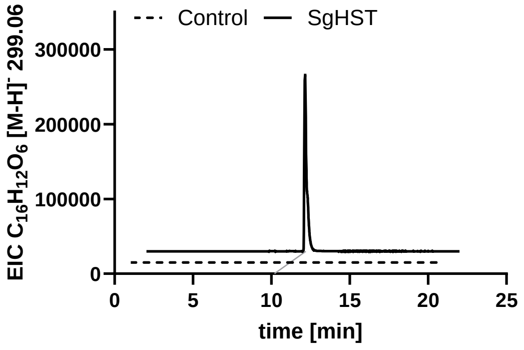

J *p*-coumaroyl-CoA + 3,4-dihydroxybenzoic acid

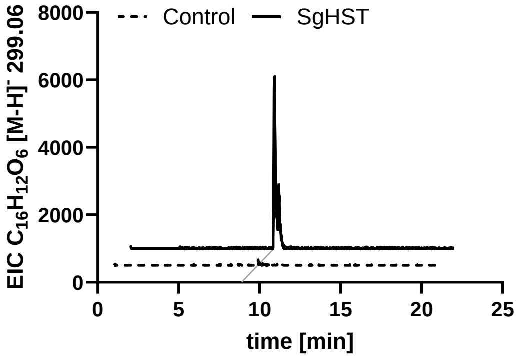

K *p*-coumaroyl-CoA + 3-aminobenzoic acid

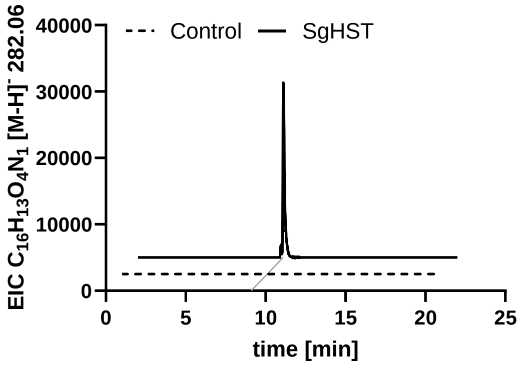

L *p*-coumaroyl-CoA + quinic acid

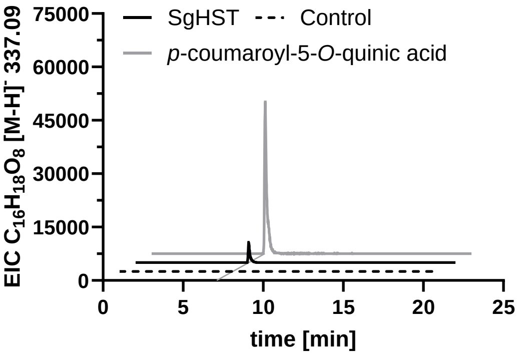

M *p*-coumaroyl-CoA + 5-hydroxyanthranilic acid

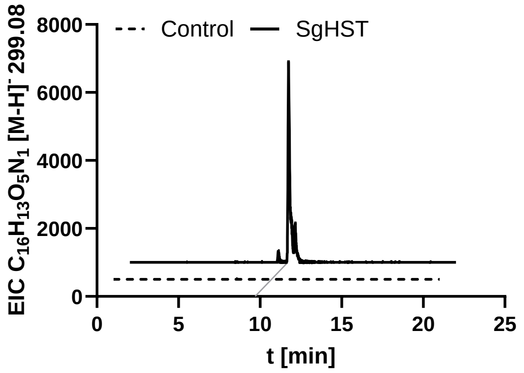

Figure S5. Extracted ion chromatograms (EIC) of enzyme assays with SgHST. The molecular formula of the expected product and the expected ion with the respective mass-to-charge ratio (*m*/*z*) are given on the Y-axis. The black line (SgHST) represents the EIC of an enzyme assay with SgHST, donor and acceptor substrate, the dashed black line (Control) refers to the EIC of an empty vector control assay, which was conducted under the same conditions in parallel. Authentic standards are depicted with a bold grey line, if they were available. The thin grey line depicts the offset included for better visualization. **A** Formation of cinnamoylshikimic acid ([M-H]$^-$ *m*/*z* 303.09); **B** formation of *p*-coumaroyl-5-*O*-shikimic acid ([M-H]$^-$ *m*/*z* 319.08); **C**

formation of caffeoyl-5-*O*-shikimic acid ([M-H]$^-$ *m*/*z* 335.08); **D** formation of feruloylshikimic acid ([M-H]$^-$ *m*/*z* 349.09); **E** formation of sinapoylshikimic acid ([M-H]$^-$ *m*/*z* 379.11); **F** formation of *p*-coumaroyl-2-*N*-3-hydroxyanthranilic acid ([M-H]$^-$ *m*/*z* 299.08); **G** formation of *p*-coumaroyl-3-hydroxybenzoic acid ([M-H]$^-$ *m*/*z* 283.06); **H** formation of *p*-coumaroyl-2,3-dihydroxybenzoic acid ([M-H]$^-$ *m*/*z* 299.06); **I** formation of *p*-coumaroyl-2,5-dihydroxybenzoic acid ([M-H]$^-$ *m*/*z* 299.06); **J** formation of *p*-coumaroyl-3,4-dihydroxybenzoic acid ([M-H]$^-$ *m*/*z* 299.06); **K** formation of *p*-coumaroyl-3-aminobenzoic acid ([M-H]$^-$ *m*/*z* 282.06); **L** formation of *p*-coumaroyl-5-*O*-quinic acid ([M-H]$^-$ *m*/*z* 337.09); **M** formation of *p*-coumaroyl-5-hydroxyanthranilic acid ([M-H]$^-$ *m*/*z* 299.08).

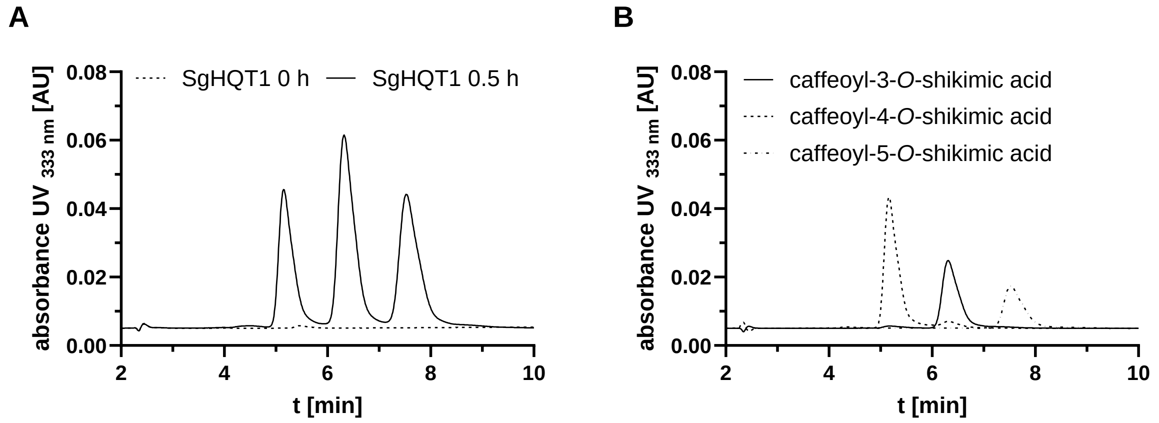

Figure S6. **A** HPLC chromatograms at 333 nm of a standard assay with SgHQT1, caffeoyl-CoA and shikimic acid and **B** 0.5 nmol of authentic standards of caffeoyl-3-*O*-shikimic acid (6.3 min), caffeoyl-4-*O*-shikimic acid (5.2 min), caffeoyl-5-*O*-shikimic acid (7.6 min).

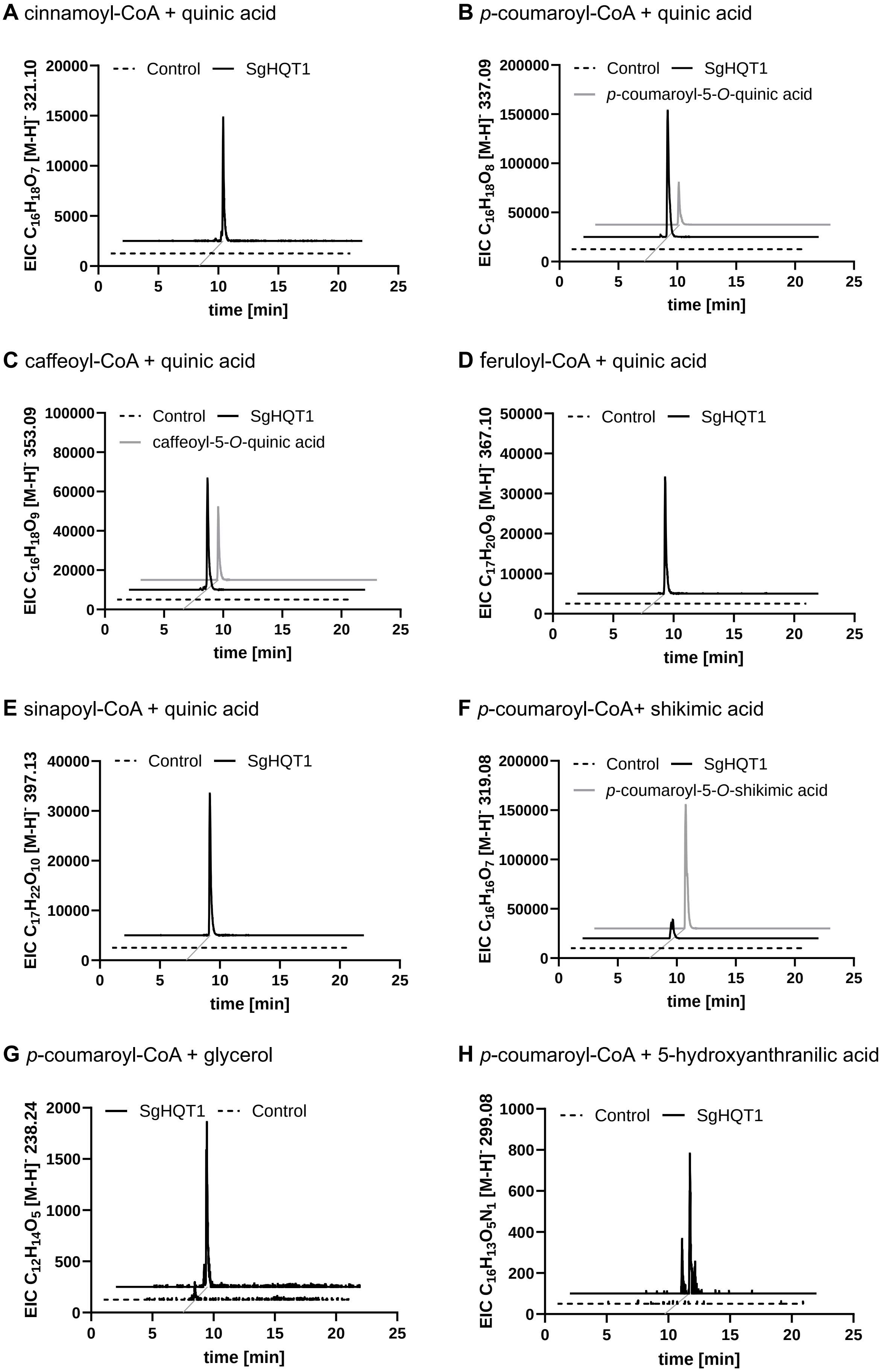
A cinnamoyl-CoA + quinic acid
Control
SgHQT1
EIC C16H18O7 [M-H]- 321.10
time [min]
B p-coumaroyl-CoA + quinic acid
Control
SgHQT1
p-coumaroyl-5-O-quinic acid
EIC C16H18O8 [M-H]- 337.09
time [min]
C caffeoyl-CoA + quinic acid
Control
SgHQT1
caffeoyl-5-O-quinic acid
EIC C16H18O9 [M-H]- 353.09
time [min]
D feruloyl-CoA + quinic acid
Control
SgHQT1
EIC C17H20O9 [M-H]- 367.10
time [min]
E sinapoyl-CoA + quinic acid
Control
SgHQT1
EIC C17H22O10 [M-H]- 397.13
time [min]
F p-coumaroyl-CoA+ shikimic acid
Control
SgHQT1
p-coumaroyl-5-O-shikimic acid
EIC C16H16O7 [M-H]- 319.08
time [min]
G p-coumaroyl-CoA + glycerol
SgHQT1
Control
EIC C12H14O5 [M-H]- 238.24
time [min]
H p-coumaroyl-CoA + 5-hydroxyanthranilic acid
Control
SgHQT1
EIC C16H13O5N1 [M-H]- 299.08
time [min]

I *p*-coumaroyl-CoA + 3-aminobenzoic acid

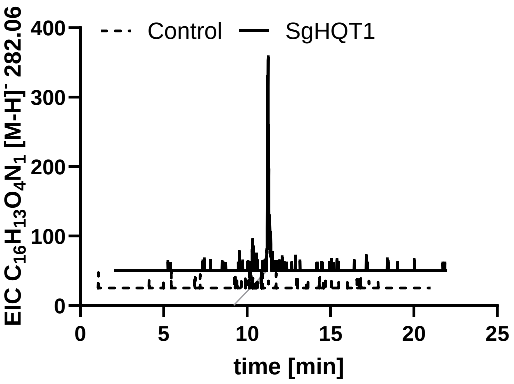

J *p*-coumaroyl-CoA + 2,3-dihydroxybenzoic acid

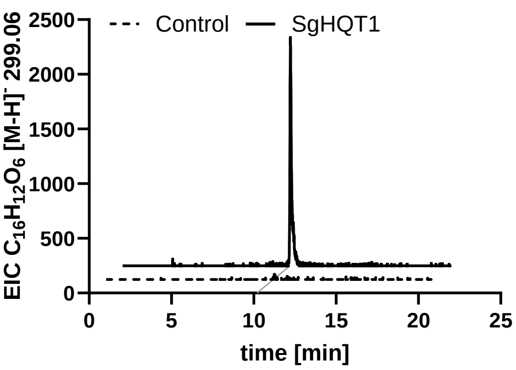

K *p*-coumaroyl-CoA + 2,5-dihydroxybenzoic acid

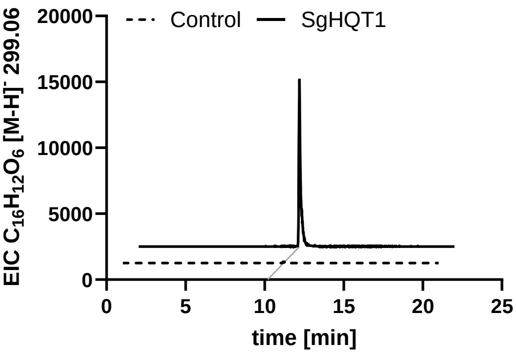

L *p*-coumaroyl-CoA + 3,4-dihydroxybenzoic acid

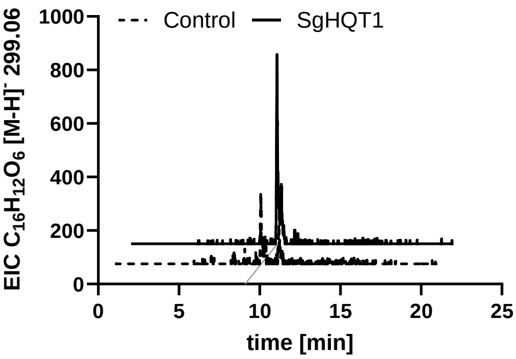

M caffeoyl-CoA + shikimic acid

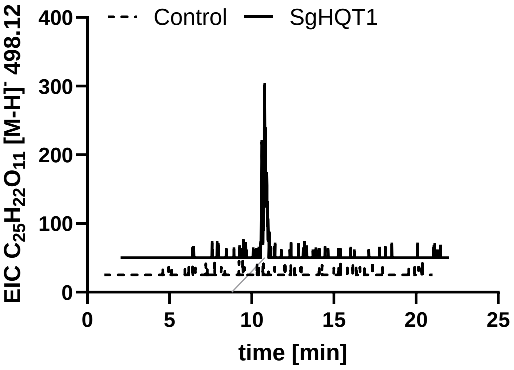

N *p*-coumaroyl-CoA + methanol

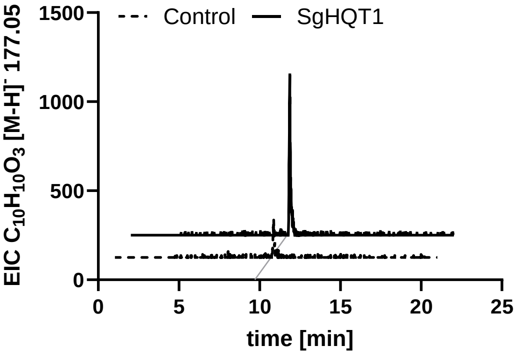

O *p*-coumaroyl-CoA + 4-hydroxybenzoic acid

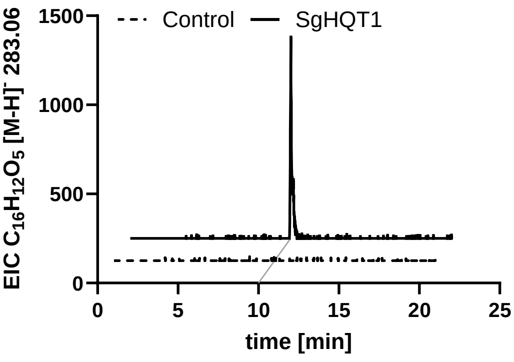

P *p*-coumaroyl-CoA + 2,4-dihydroxybenzoic acid

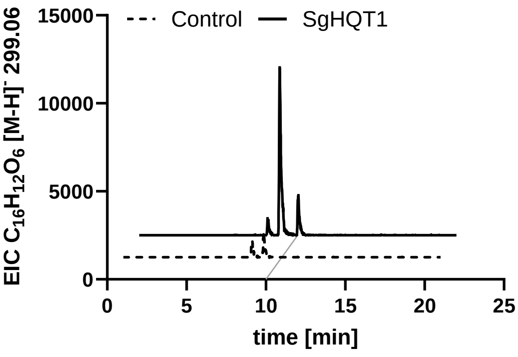

Q *p*-coumaroyl-CoA + 3-hydroxyanthranilic acid **R** caffeoyl-CoA + shikimic acid

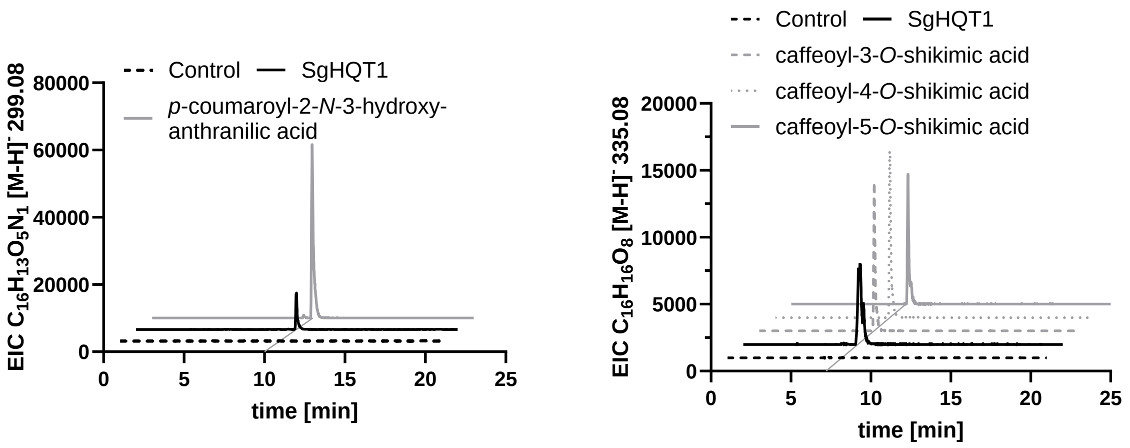

Figure S7. Extracted ion chromatograms (EIC) of enzyme assays with SgHQT1. The molecular formula of the expected product and the expected ion with the respective mass-to-charge ratio (*m*/*z*) is given on the Y-axis. The black line (SgHQT1) represents the EIC of an enzyme assay with SgHQT1, donor and acceptor substrate, the dashed black line (Control) refers to the EIC of an empty vector control assay, which was conducted under the same conditions in parallel. Authentic standards are depicted with a bold grey line, if they were available. The thin grey line depicts the offset included for better visualization. **A** Formation of cinnamoylquinic acid ($[M\text{-}H]^-$ *m*/*z* 321.10); **B** formation of *p*-coumaroyl-5-*O*-quinic acid ($[M\text{-}H]^-$ *m*/*z* 337.09); **C** formation of caffeoyl-5-*O*-quinic acid ($[M\text{-}H]^-$ *m*/*z* 353.09); **D** formation of feruloylquinic acid ($[M\text{-}H]^-$ *m*/*z* 367.10); **E** formation of sinapoylquinic acid ($[M\text{-}H]^-$ *m*/*z* 397.13); **F** formation of *p*-coumaroyl-3-, -4-, and -5-*O*-shikimic acid ($[M\text{-}H]^-$ *m*/*z* 319.08); the retention times are nearly indistinguishable, but the product peak shows three tips. **G** formation of *p*-coumaroylglycerol ($[M\text{-}H]^-$ *m*/*z* 238.24); **H** formation of *p*-coumaroyl-5-hydroxyanthranilic acid ($[M\text{-}H]^-$ *m*/*z* 299.08); **I** formation of *p*-coumaroyl-3-aminobenzoic acid ($[M\text{-}H]^-$ *m*/*z* 282.06); **J** formation of *p*-coumaroyl-2,3-dihydroxybenzoic acid ($[M\text{-}H]^-$ *m*/*z* 299.06); **K** formation of *p*-coumaroyl-2,5-dihydroxybenzoic ($[M\text{-}H]^-$ *m*/*z* 299.06); **L** formation of *p*-coumaroyl-3,4-dihydroxybenzoic ($[M\text{-}H]^-$ *m*/*z* 299.06); **M** formation of dicaffeoylshikimic acid ($[M\text{-}H]^-$ *m*/*z* 498.12); **N** formation of caffeoylmethanol ($[M\text{-}H]^-$ *m*/*z* 177.05); **O** formation of *p*-coumaroyl-4-hydroxybenzoic acid ($[M\text{-}H]^-$ *m*/*z* 283.06); **P** formation of *p*-coumaroyl-2,4-dihydroxybenzoic acid ($[M\text{-}H]^-$ *m*/*z* 299.06). Three peaks were detected, only the third peak (retention time of 10.0 min) had the expected absorbance maximum of 332 nm; **Q** formation of *p*-coumaroyl-2-*N*-3-hydroxyanthranilic acid ($[M\text{-}H]^-$ *m*/*z* 283.06); **R** formation of caffeoyl-3-, -4- and -5-*O*-shikimic acid ($[M\text{-}H]^-$ *m*/*z* 335.08); the retention times are nearly indistinguishable, but the product peak shows three tips (see Figure S6).

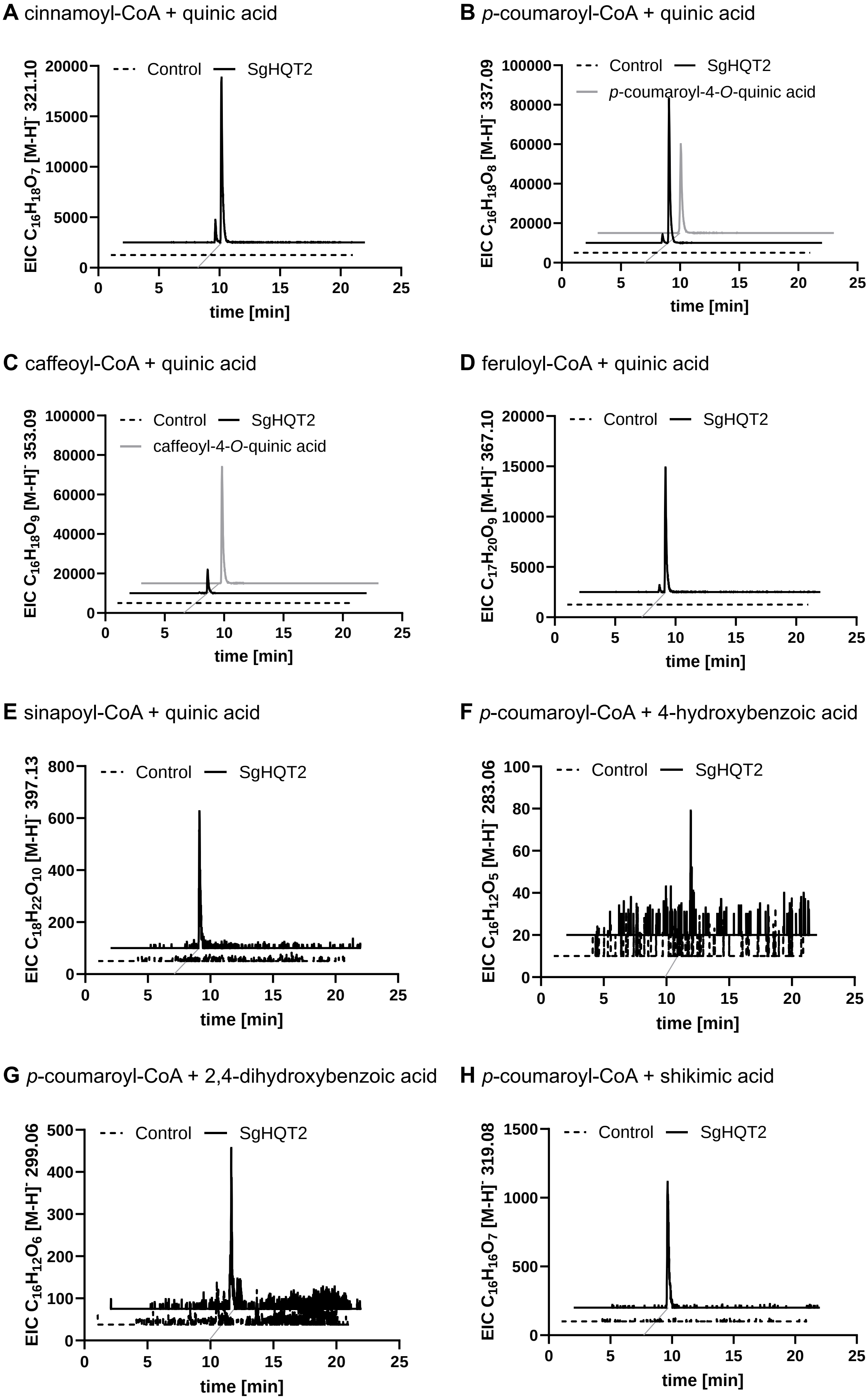
A cinnamoyl-CoA + quinic acid
EIC C16H18O7 [M-H]- 321.10
Control
SgHQT2
time [min]
B p-coumaroyl-CoA + quinic acid
EIC C16H18O8 [M-H]- 337.09
Control
SgHQT2
p-coumaroyl-4-O-quinic acid
time [min]
C caffeoyl-CoA + quinic acid
EIC C16H18O9 [M-H]- 353.09
Control
SgHQT2
caffeoyl-4-O-quinic acid
time [min]
D feruloyl-CoA + quinic acid
EIC C17H20O9 [M-H]- 367.10
Control
SgHQT2
time [min]
E sinapoyl-CoA + quinic acid
EIC C18H22O10 [M-H]- 397.13
Control
SgHQT2
time [min]
F p-coumaroyl-CoA + 4-hydroxybenzoic acid
EIC C16H12O5 [M-H]- 283.06
Control
SgHQT2
time [min]
G p-coumaroyl-CoA + 2,4-dihydroxybenzoic acid
EIC C16H12O6 [M-H]- 299.06
Control
SgHQT2
time [min]
H p-coumaroyl-CoA + shikimic acid
EIC C16H16O7 [M-H]- 319.08
Control
SgHQT2
time [min]

I caffeoyl-CoA + shikimic acid

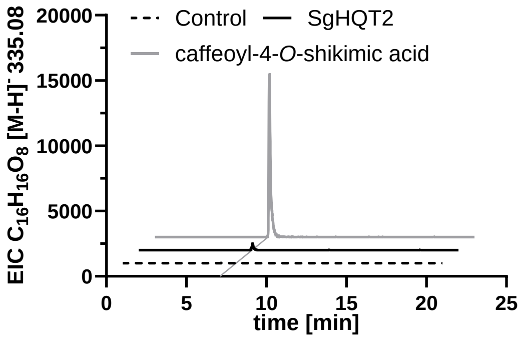

Figure S8. Extracted ion chromatograms (EIC) of enzyme assays with SgHQT2. The molecular formula of the expected product and the expected ion with the respective mass-to-charge ratio (*m*/*z*) is given on the Y-axis. The black line (SgHQT2) represents the EIC of an enzyme assay with SgHQT2, donor and acceptor substrate, the dashed black line (Control) refers to the EIC of an empty vector control assay, which was conducted under the same conditions in parallel. Authentic standards are depicted with a bold grey line, if they were available. The thin grey line depicts the offset included for better visualization. **A** Formation of cinnamoylquinic acid ([M-H]$^-$ *m*/*z* 321.10); **B** formation of *p*-coumaroyl-4-*O*-quinic acid ([M-H]$^-$ *m*/*z* 337.09); **C** formation of caffeoyl-4-*O*-quinic acid ([M-H]$^-$ *m*/*z* 353.09); **D** formation of feruloylquinic acid ([M-H]$^-$ *m*/*z* 367.10); **E** formation of sinapoylquinic acid ([M-H]$^-$ *m*/*z* 397.13); **F** formation of *p*-coumaroyl-4-hydroxybenzoic acid ([M-H]$^-$ *m*/*z* 283.06); **G** formation of *p*-coumaroyl-2,4-dihydroxybenzoic acid ([M-H]$^-$ *m*/*z* 299.06); **H** formation of *p*-coumaroylshikimic acid ([M-H]$^-$ *m*/*z* 319.08); **I** formation of caffeoyl-4-*O*-shikimic acid ([M-H]$^-$ *m*/*z* 335.08).

A cinnamoyl-CoA + (*RS*)-4-hydroxyphenyl-lactic acid

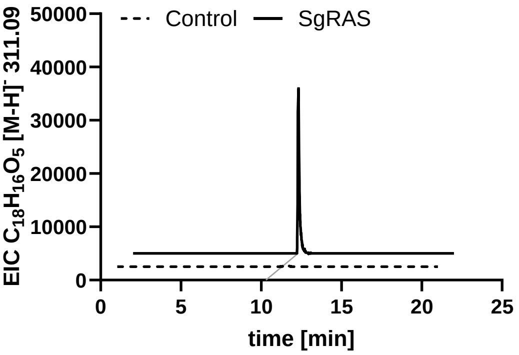

B *p*-coumaroyl-CoA + (*RS*)-4-hydroxyphenyl-lactic acid

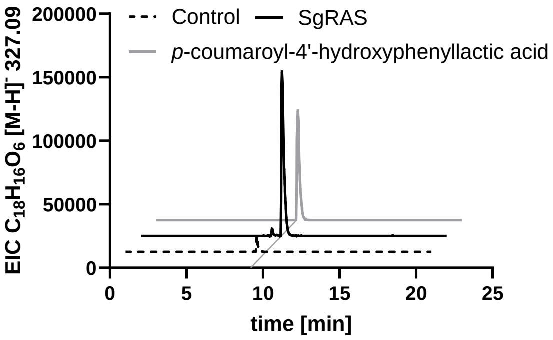

C caffeoyl-CoA + (*RS*)-4-hydroxyphenyl-lactic acid

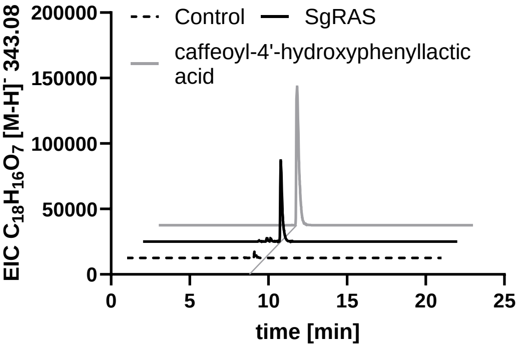

D feruloyl-CoA + (*RS*)-4-hydroxyphenyl-lactic acid

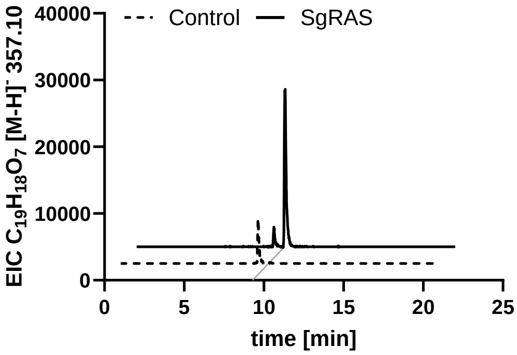

E sinapoyl-CoA + (*RS*)-4-hydroxyphenyl-lactic acid

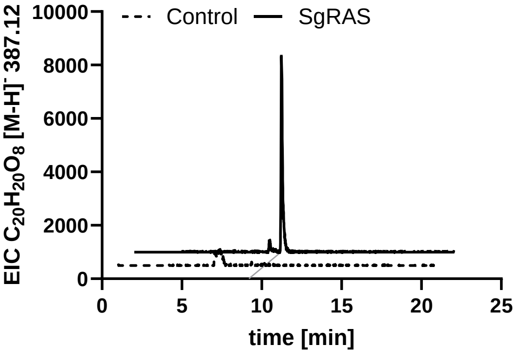

F *p*-coumaroyl-CoA + (*RS*)-phenyllactic acid

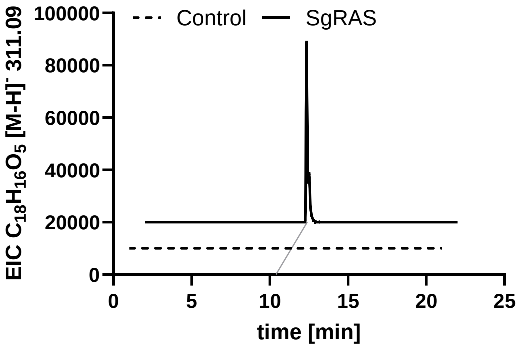

G *p*-coumaroyl-CoA + (*RS*)-3,4-dihydroxyphenyllactic acid

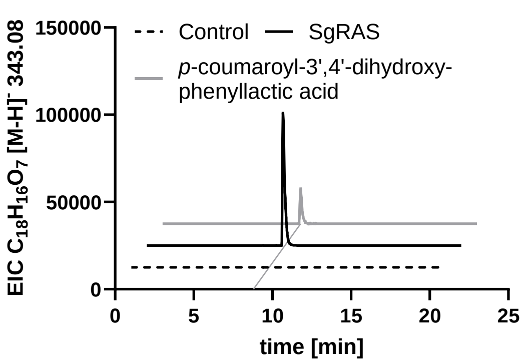

H *p*-coumaroyl-CoA + (*RS*)-4-hydroxy-3-methoxyphenyllactic acid

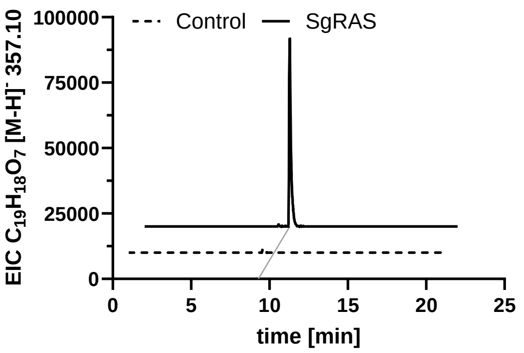

I *p*-coumaroyl-CoA + D-phenylalanine

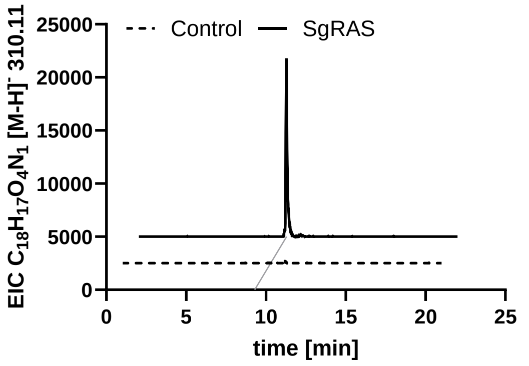

J *p*-coumaroyl-CoA + D-tyrosine

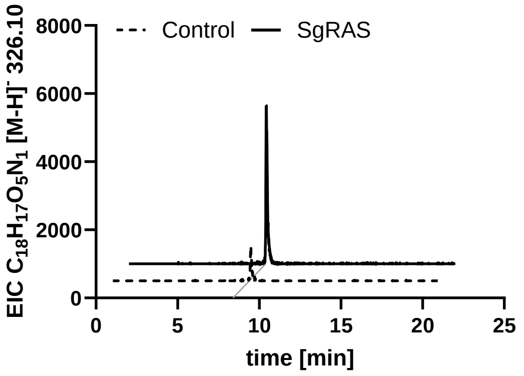

K *p*-coumaroyl-CoA + D/L-3,4-dihydroxyphenylalanine

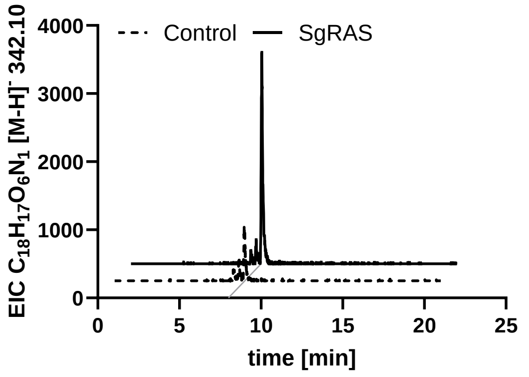

Figure S9. Extracted ion chromatograms (EIC) of enzyme assays with SgRAS. The molecular formula of the expected product and the expected ion with the respective mass-to-charge ratio (*m*/*z*) is given on the Y-axis. The black line (SgRAS) represents the EIC of an enzyme assay with SgRAS, donor and acceptor substrate, the dashed black line (Control) refers to the EIC of an empty vector control assay, which was conducted under the same conditions in parallel. Authentic standards are depicted with a bold grey line, if they were available. The thin grey line depicts the offset included for better visualization. **A** Formation of cinnamoyl-4'-hydroxyphenyllactic acid ($[M-H]^-$ *m*/*z* 311.09); **B** formation of *p*-coumaroyl-4'-

hydroxyphenyllactic acid ($[M-H]^-$ *m*/*z* 327.09); **C** formation of caffeoyl-4’-hydroxyphenyllactic acid ($[M-H]^-$ *m*/*z* 343.08; **D** formation of feruloyl-4’-hydroxyphenyllactic acid ($[M-H]^-$ *m*/*z* 357.10); **E** formation of sinapoyl-4’-hydroxyphenyllactic acid ($[M-H]^-$ *m*/*z* 387.12); **F** formation of *p*-coumaroylphenyllactic acid ($[M-H]^-$ *m*/*z* 311.09); **G** formation of *p*-coumaroyl-3’,4’-dihydroxyphenyllactic acid ($[M-H]^-$ *m*/*z* 343.08); **H** formation of *p*-coumaroyl-4’-hydroxy-3’-methoxyphenyllactic acid ($[M-H]^-$ *m*/*z* 357.10); **I** formation of *p*-coumaroyl-D-phenylalanine ($[M-H]^-$ *m*/*z* 310.11); **J** formation of *p*-coumaroyl-D-tyrosine ($[M-H]^-$ *m*/*z* 326.10); **K** formation of *p*-coumaroyl-D-3’,4’-dihydroxyphenylalanine ($[M-H]^-$ *m*/*z* 342.10).

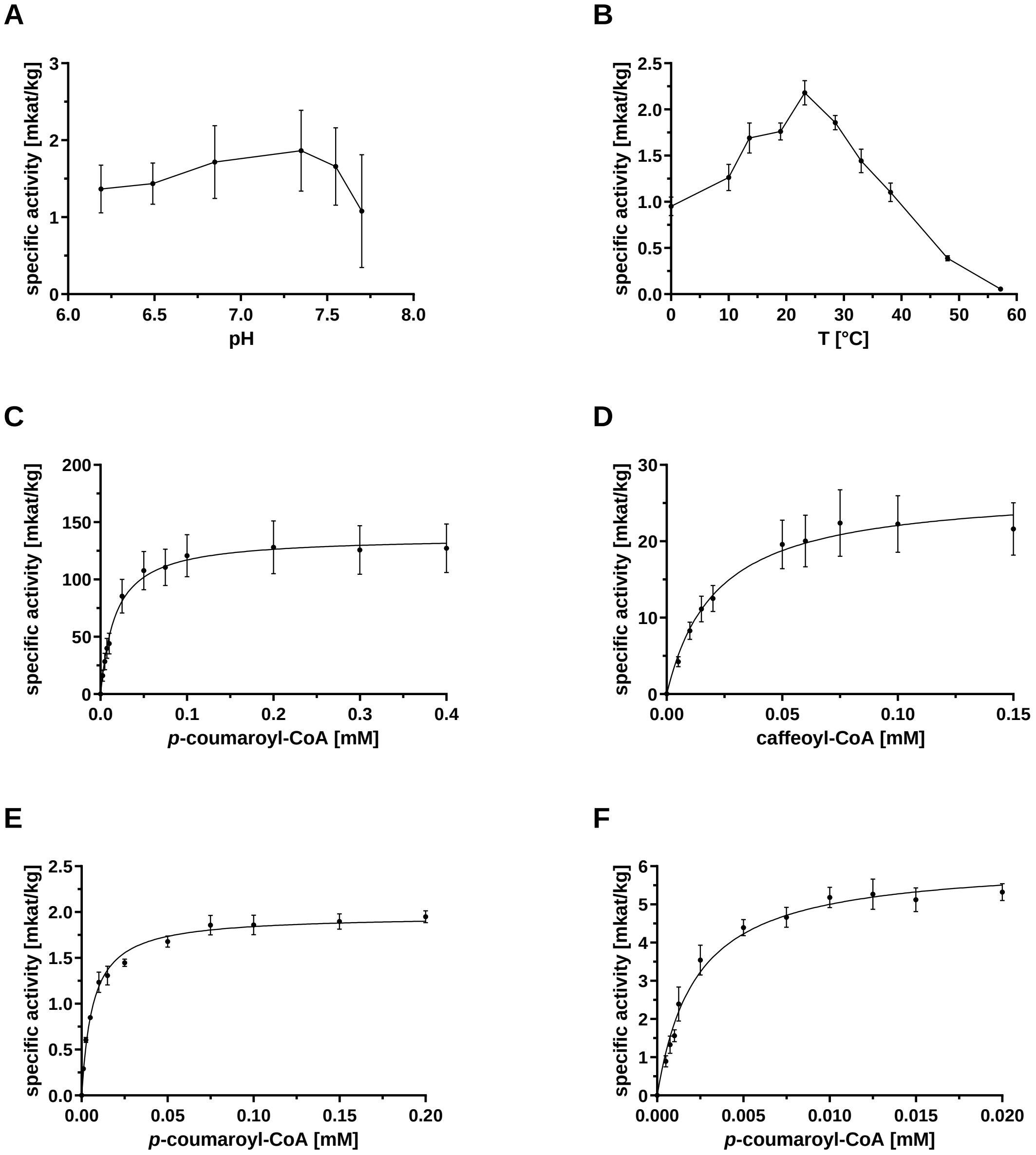

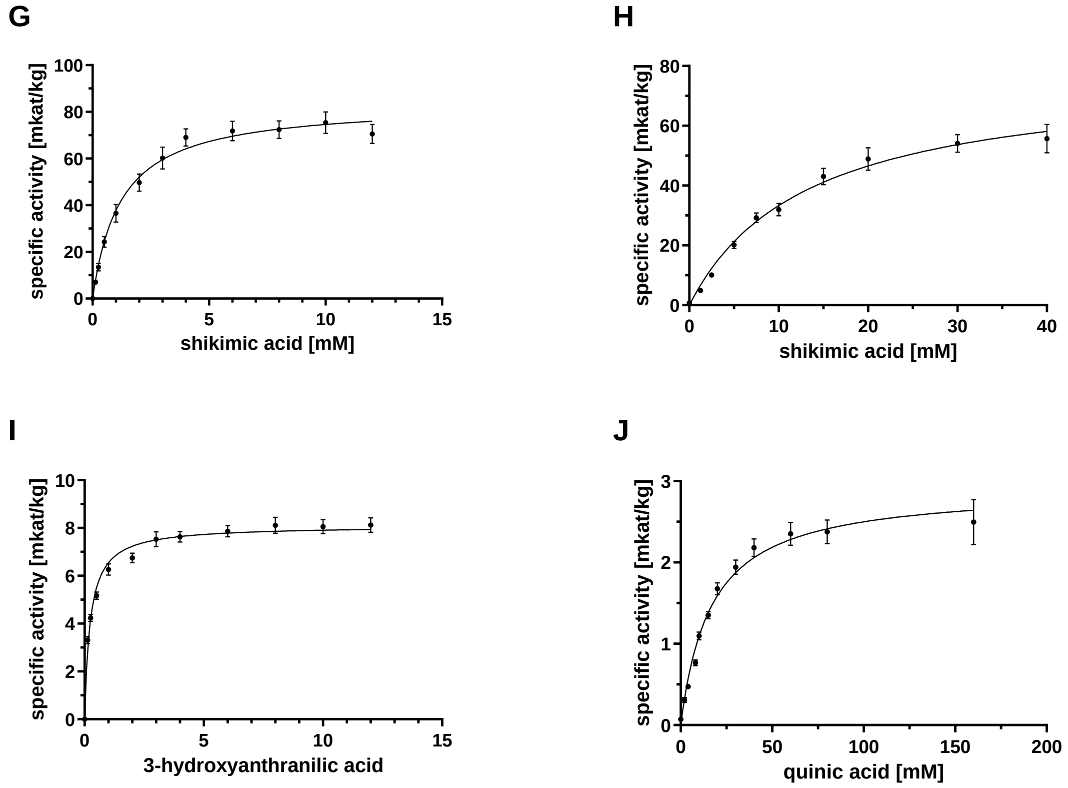

Figure S10. pH-Optimum, temperature optimum and Michaelis-Menten kinetics (K_m and V_{max}) for SgHST. For exact reaction conditions, see Table S12. **A**: pH-optimum (*n* = 3 ± SD), **B**: temperature optimum (*n* = 3 ± SD), **C**: substrate saturation curve for *p*-coumaroyl-CoA with 16 mM shikimic acid (*n* = 9 ± SEM), **D**: substrate saturation curve for caffeoyl-CoA with 30 mM shikimic acid (*n* = 9 ± SEM), **E**: substrate saturation curve for *p*-coumaroyl-CoA with 160 mM quinic acid (*n* = 9 ± SEM), **F**: substrate saturation curve for *p*-coumaroyl-CoA with 6 mM 3-hydroxyanthranilic acid (*n* = 9 ± SEM), **G**: substrate saturation curve for shikimic acid with 200 µM *p*-coumaroyl-CoA (*n* = 9 ± SEM), **H**: substrate saturation curve for shikimic acid with 300 µM caffeoyl-CoA (*n* = 9 ± SEM), **I**: substrate saturation curve for quinic acid with 200 µM *p*-coumaroyl-CoA (*n* = 9 ± SEM), **J**: substrate saturation curve for 3-hydroxyanthranilic acid with 80 µM *p*-coumaroyl-CoA (*n* = 9 ± SEM).

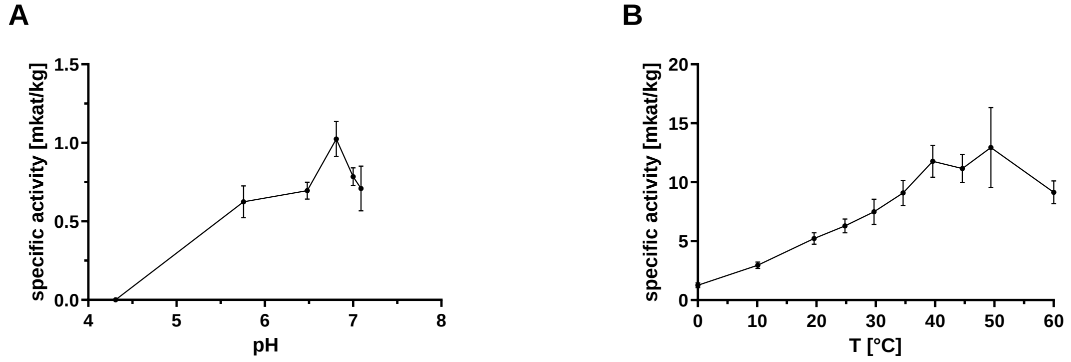

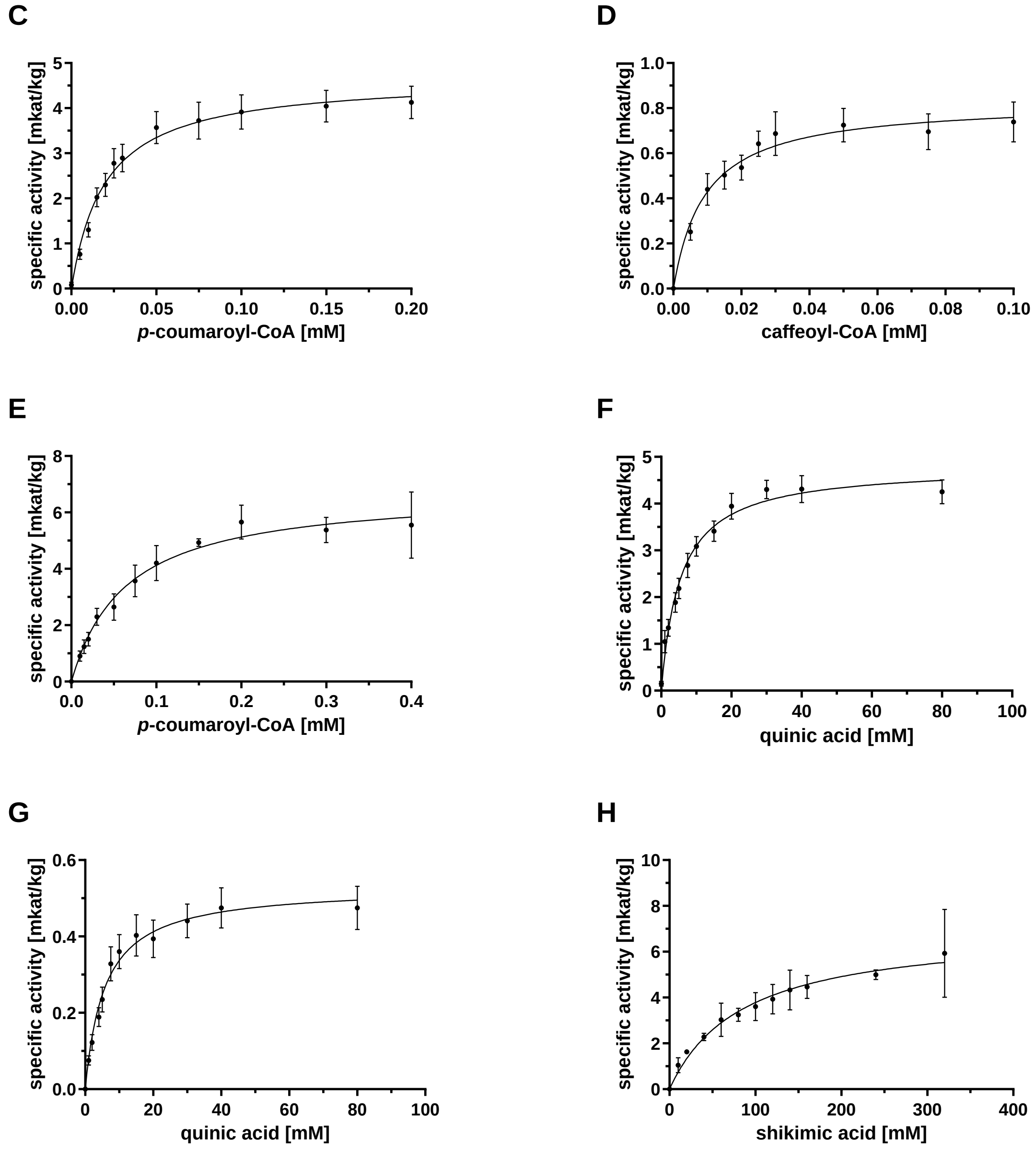

Figure S11. pH-Optimum, temperature optimum and Michaelis-Menten kinetics (K_m and V_{max}) for SgHQT1. For exact reaction conditions, see Table S13. **A**: pH-optimum (*n* = 3 ± SD), **B**: temperature optimum (*n* = 3 ± SD), **C**: substrate saturation curve for *p*-coumaroyl-CoA with 80 mM quinic acid (*n* = 9 ± SEM), **D**: substrate saturation curve for caffeoyl-CoA with 80 mM quinic acid (*n* = 9 ± SEM), **E**: substrate saturation curve for *p*-coumaroyl-CoA with 320 mM shikimic acid (*n* = 9 ± SEM), **F**: substrate saturation curve for quinic acid with 200 µM *p*-coumaroyl-CoA (*n* = 9 ± SEM), **G**: substrate saturation curve for quinic acid with 100 µM caffeoyl-CoA (*n* = 9 ± SEM), **H**: substrate saturation curve for shikimic acid with 400 µM *p*-coumaroyl-CoA (*n* = 9 ± SEM).

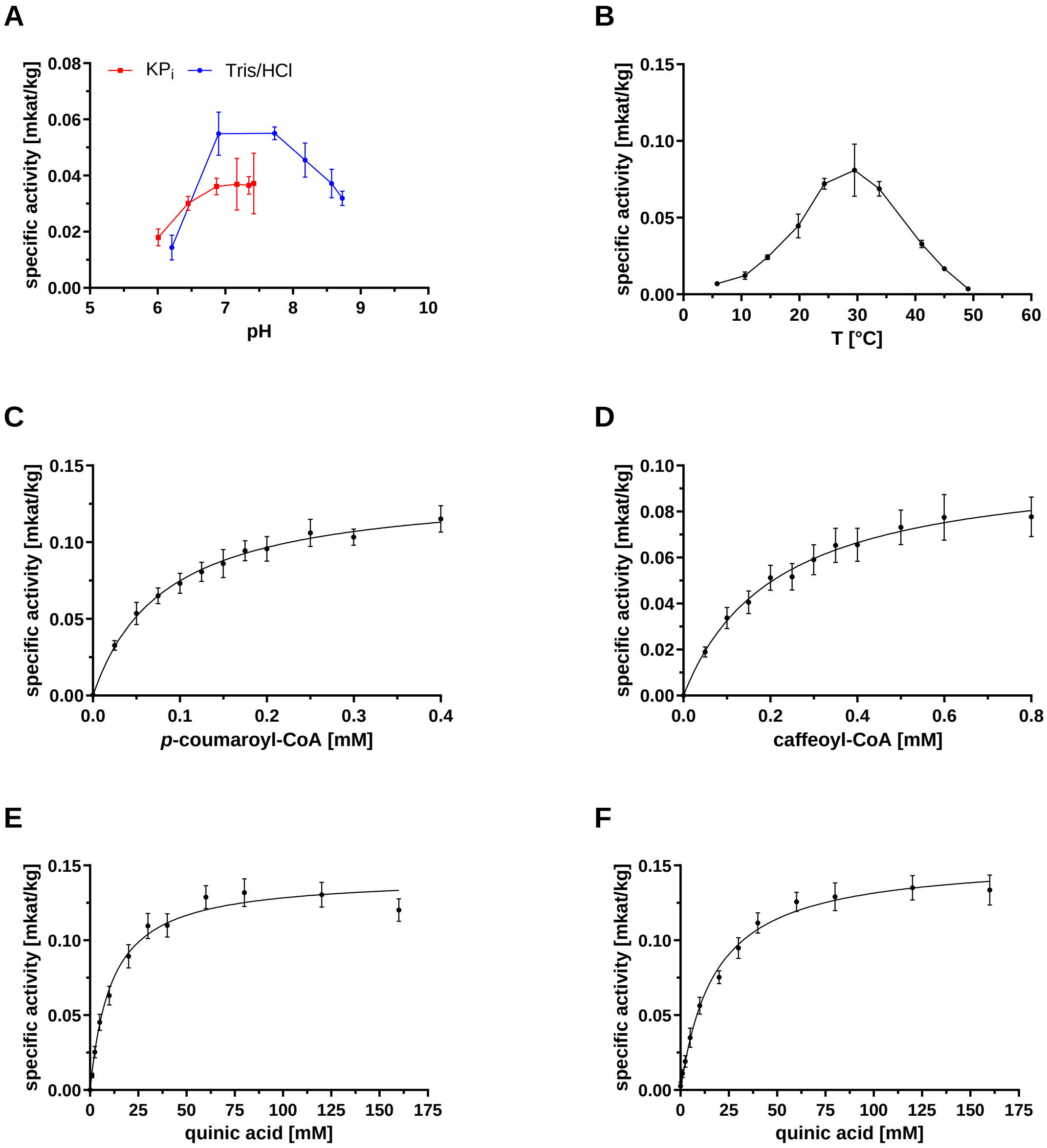

Figure S12. pH-Optimum, temperature optimum and Michaelis-Menten kinetics (K_m and V_{max}) for SgHQT2. For exact reaction conditions, see Table S14. **A**: pH-optimum (red: in 0.1 M K_2HPO_4/KH_2PO_4 (KP_i), blue: in 0.1 M Tris-HCl (Tris/HCl)) ($n = 3 \pm$ SD), **B**: temperature optimum ($n = 3 \pm$ SD), **C**: substrate saturation curve for *p*-coumaroyl-CoA with 160 mM quinic acid ($n = 9 \pm$ SEM), **D**: substrate saturation curve for caffeoyl-CoA with 160 mM quinic acid ($n = 9 \pm$ SEM), **E**: substrate saturation curve for quinic acid with 400 µM *p*-coumaroyl-CoA ($n = 9 \pm$ SEM), **F**: substrate saturation curve for quinic acid with 800 µM caffeoyl-CoA ($n = 9 \pm$ SEM).

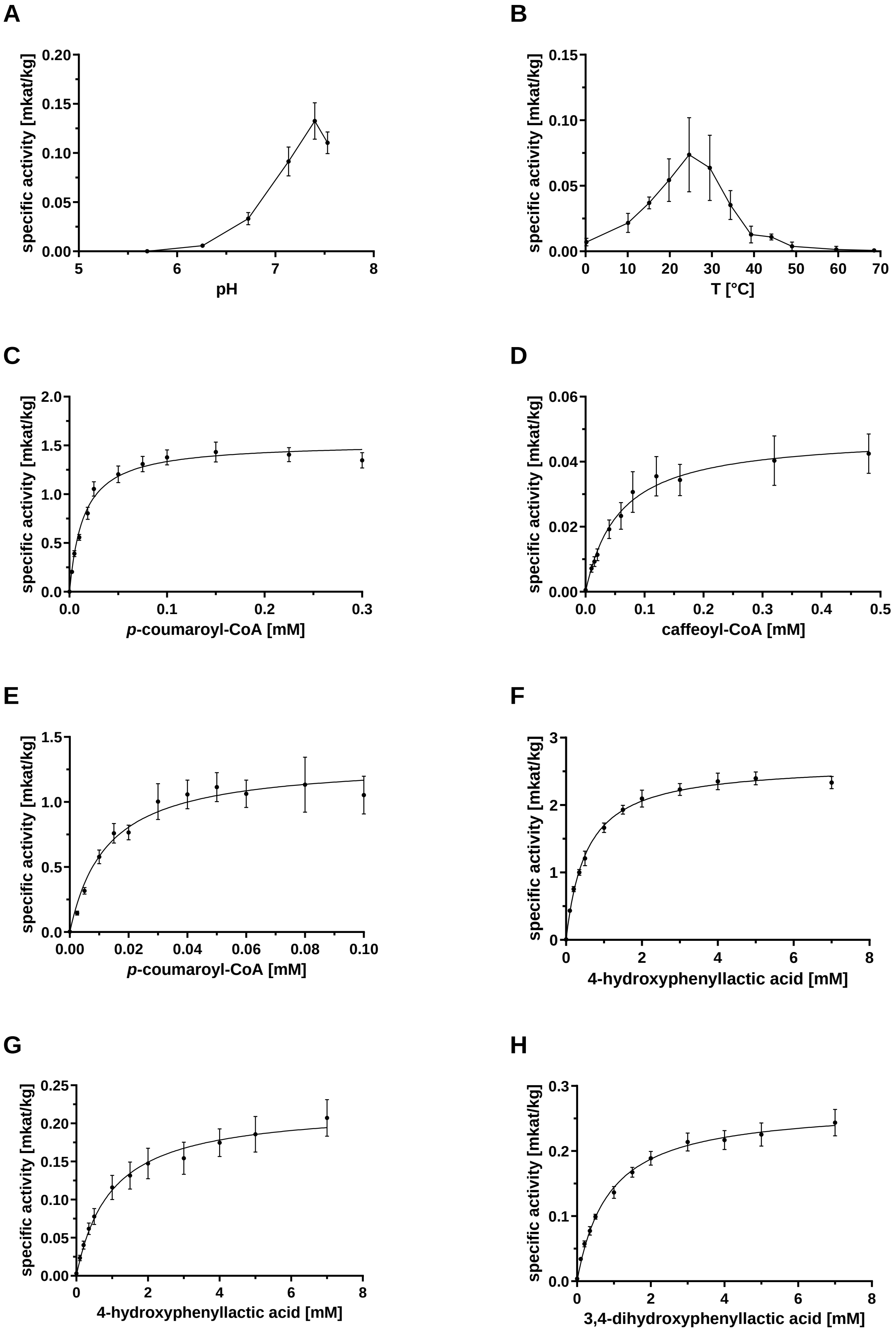

Figure S13. pH-Optimum, temperature optimum and Michaelis-Menten kinetics (K_m and V_{max}) for SgRAS. For exact reaction conditions see Table S15. **A**: pH-optimum (n = 3 ± SD), **B**: temperature optimum (n = 3 ± SD), **C**: substrate saturation curve for *p*-coumaroyl-CoA with 4

mM 4-hydroxyphenyllactic acid (*n* = 9 ± SEM), **D**: substrate saturation curve for caffeoyl-CoA with 4 mM 4-hydroxyphenyllactic acid (*n* = 9 ± SEM), **E**: substrate saturation curve for *p*-coumaroyl-CoA with 4 mM 3,4-dihydroxyphenyllactic acid (*n* = 9 ± SEM), **F**: substrate saturation curve for 4-hydroxyphenyllactic acid with 200 µM *p*-coumaroyl-CoA (*n* = 9 ± SEM), **G**: substrate saturation curve for 4-hydroxyphenyllactic acid with 400 µM caffeoyl-CoA (*n* = 9 ± SEM), **H**: substrate saturation curve for 3,4-dihydroxyphenyllactic acid with 100 µM *p*-coumaroyl-CoA (*n* = 9 ± SEM).

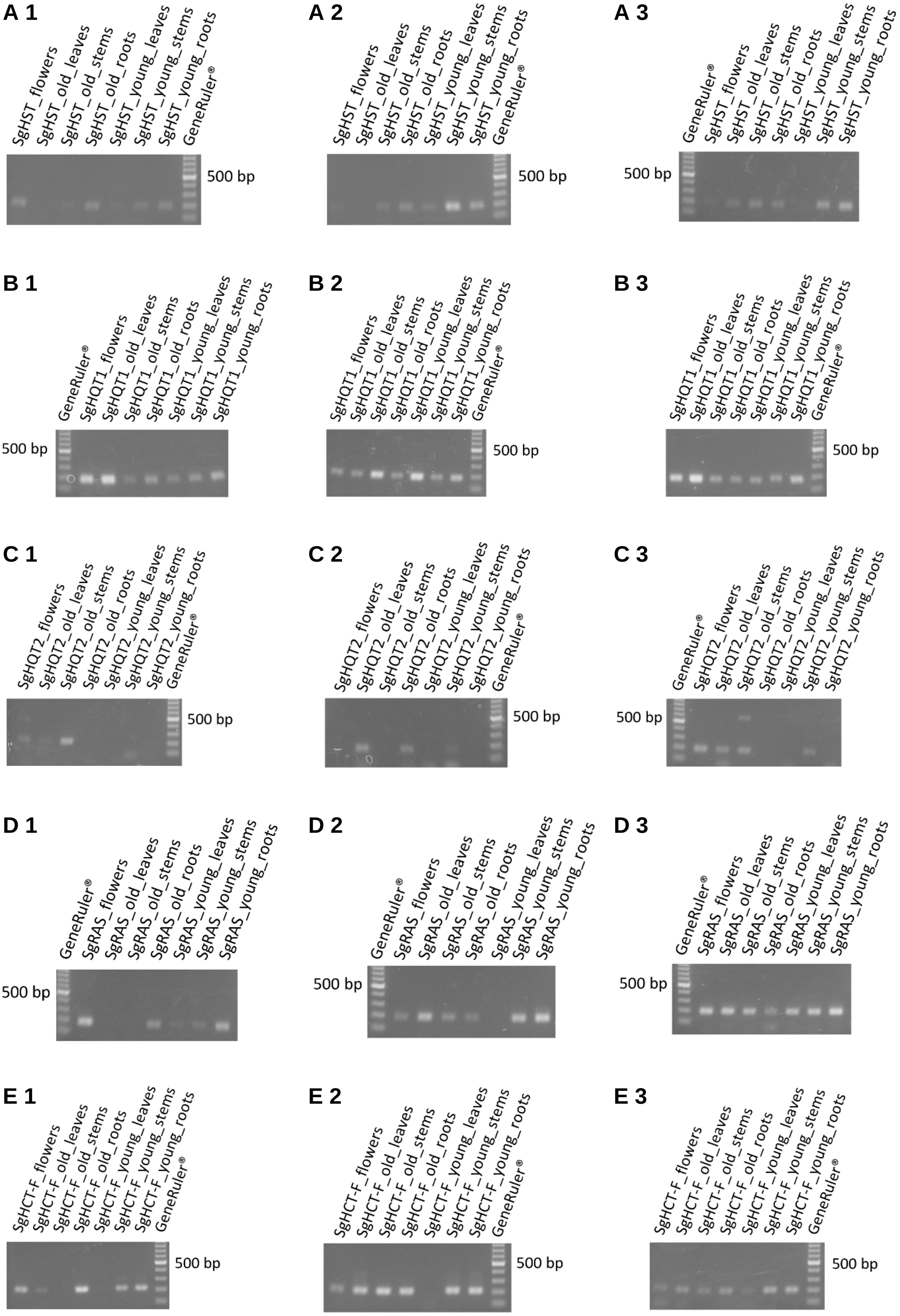
A 1
SgHST_flowers
SgHST_old_leaves
SgHST_old_stems
SgHST_old_roots
SgHST_young_leaves
SgHST_young_stems
SgHST_young_roots
GeneRuler®
500 bp
A 2
SgHST_flowers
SgHST_old_leaves
SgHST_old_stems
SgHST_old_roots
SgHST_young_leaves
SgHST_young_stems
SgHST_young_roots
GeneRuler®
500 bp
A 3
GeneRuler®
SgHST_flowers
SgHST_old_leaves
SgHST_old_stems
SgHST_old_roots
SgHST_young_leaves
SgHST_young_stems
SgHST_young_roots
500 bp
B 1
GeneRuler®
SgHQT1_flowers
SgHQT1_old_leaves
SgHQT1_old_stems
SgHQT1_old_roots
SgHQT1_young_leaves
SgHQT1_young_stems
SgHQT1_young_roots
500 bp
B 2
SgHQT1_flowers
SgHQT1_old_leaves
SgHQT1_old_stems
SgHQT1_old_roots
SgHQT1_young_leaves
SgHQT1_young_stems
SgHQT1_young_roots
GeneRuler®
500 bp
B 3
SgHQT1_flowers
SgHQT1_old_leaves
SgHQT1_old_stems
SgHQT1_old_roots
SgHQT1_young_leaves
SgHQT1_young_stems
SgHQT1_young_roots
GeneRuler®
500 bp
C 1
SgHQT2_flowers
SgHQT2_old_leaves
SgHQT2_old_stems
SgHQT2_old_roots
SgHQT2_young_leaves
SgHQT2_young_stems
SgHQT2_young_roots
GeneRuler®
500 bp
C 2
SgHQT2_flowers
SgHQT2_old_leaves
SgHQT2_old_stems
SgHQT2_old_roots
SgHQT2_young_leaves
SgHQT2_young_stems
SgHQT2_young_roots
GeneRuler®
500 bp
C 3
GeneRuler®
SgHQT2_flowers
SgHQT2_old_leaves
SgHQT2_old_stems
SgHQT2_old_roots
SgHQT2_young_leaves
SgHQT2_young_stems
SgHQT2_young_roots
500 bp
D 1
GeneRuler®
SgRAS_flowers
SgRAS_old_leaves
SgRAS_old_stems
SgRAS_old_roots
SgRAS_young_leaves
SgRAS_young_stems
SgRAS_young_roots
500 bp
D 2
GeneRuler®
SgRAS_flowers
SgRAS_old_leaves
SgRAS_old_stems
SgRAS_old_roots
SgRAS_young_leaves
SgRAS_young_stems
SgRAS_young_roots
500 bp
D 3
GeneRuler®
SgRAS_flowers
SgRAS_old_leaves
SgRAS_old_stems
SgRAS_old_roots
SgRAS_young_leaves
SgRAS_young_stems
SgRAS_young_roots
500 bp
E 1
SgHCT-F_flowers
SgHCT-F_old_leaves
SgHCT-F_old_stems
SgHCT-F_old_roots
SgHCT-F_young_leaves
SgHCT-F_young_stems
SgHCT-F_young_roots
GeneRuler®
500 bp
E 2
SgHCT-F_flowers
SgHCT-F_old_leaves
SgHCT-F_old_stems
SgHCT-F_old_roots
SgHCT-F_young_leaves
SgHCT-F_young_stems
SgHCT-F_young_roots
GeneRuler®
500 bp
E 3
SgHCT-F_flowers
SgHCT-F_old_leaves
SgHCT-F_old_stems
SgHCT-F_old_roots
SgHCT-F_young_leaves
SgHCT-F_young_stems
SgHCT-F_young_roots
GeneRuler®
500 bp

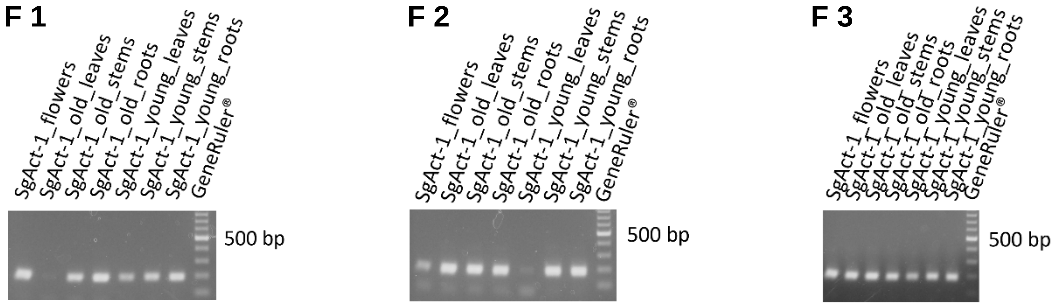

Figure S14. Agarose gels used for analyzing relative expression of SgHCTs in different tissues (flowers, old leaves, old stems, old roots, young leaves, young stems, young roots) of *Sarcandra glabra*. The brightness of the bands was evaluated using ImageJ and the expression, relative to SgAct-1 in the respective tissue (Figure 4 F), is displayed in Figure 4 as the mean of three replicates ($n = 3 \pm$ SD). Primer sequences and PCR conditions are shown in Table S21. The correct size of the amplicons was checked with GeneRuler as standard DNA ladder. **A** to **F** represent replicates 1 to 3 from SgHCTs and SgAct-1. **A 1-3**: SgHST, **B 1-3**: SgHQT1, **C 1-3**: SgHQT2, **D 1-3**: SgRAS, **E 1-3**: SgHCT-F, **F 1-3**: SgAct-1.

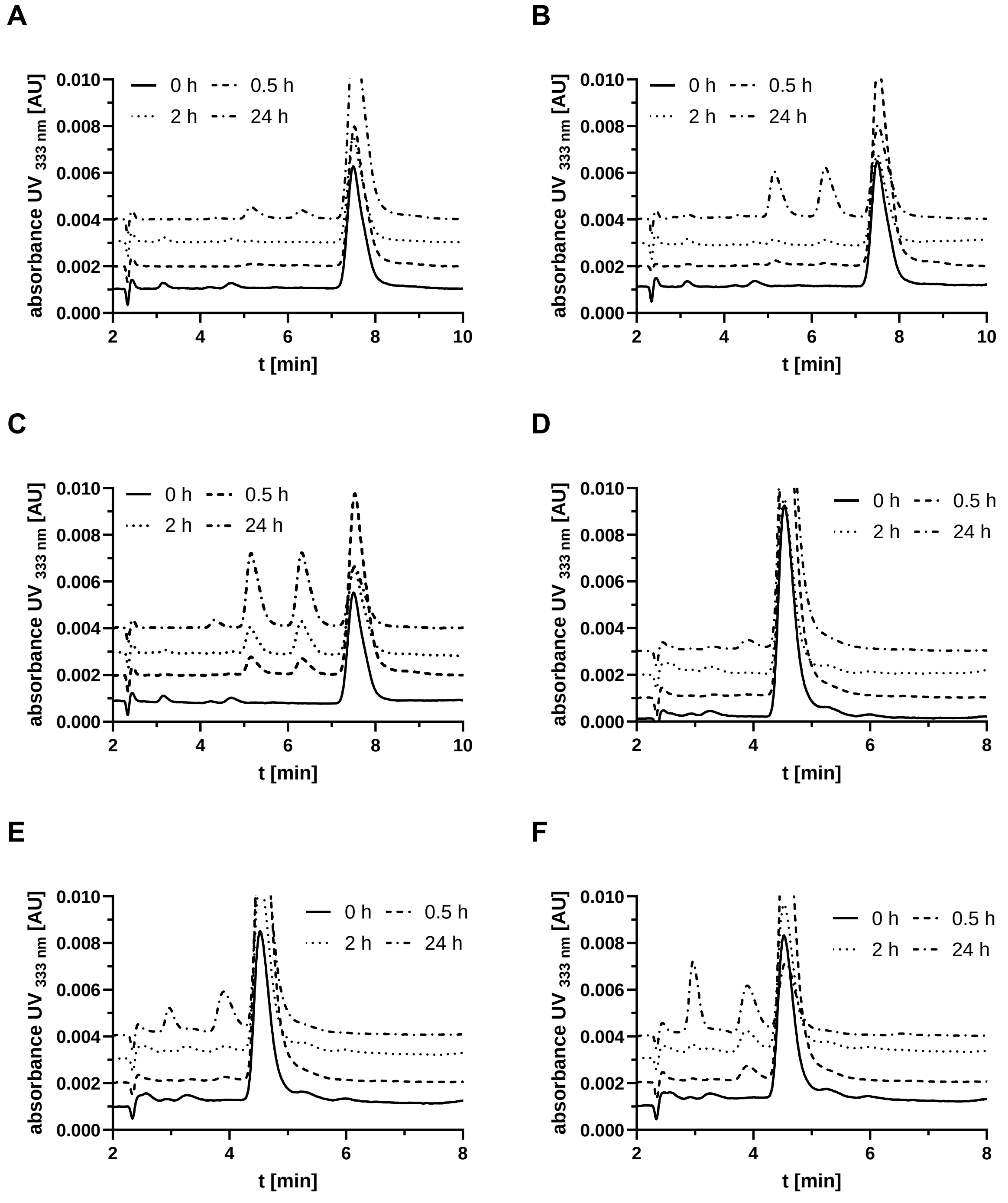

Figure S15. HPLC chromatograms recorded at 333 nm showing the acyl migration for chlorogenic acid and caffeoyl-5-*O*-shikimic acid at three pH values over time. The retention times of the peaks were: caffeoyl-3-*O*-shikimic acid – 6.3 min, caffeoyl-4-*O*-shikimic acid – 5.2 min, caffeoyl-5-*O*-shikimic acid – 7.6 min, neochlorogenic acid – 3.0 min, cryptochlorogenic acid – 3.9 min, chlorogenic acid – 4.5 min. Caffeoyl-5-*O*-shikimic acid was incubated in 0.1 M KH_2PO_4/K_2HPO_4 buffer for 0, 0.5, 2 and 24 h at pH 6.0 (**A**), pH 7.0 (**B**) and pH 8.0 (**C**). Chlorogenic acid was incubated in 0.1 M KH_2PO_4/K_2HPO_4 buffer for 0, 0.5, 2 and 24 h at pH 6.0 (**D**), pH 7.0 (**E**) and pH 8.0 (**F**).

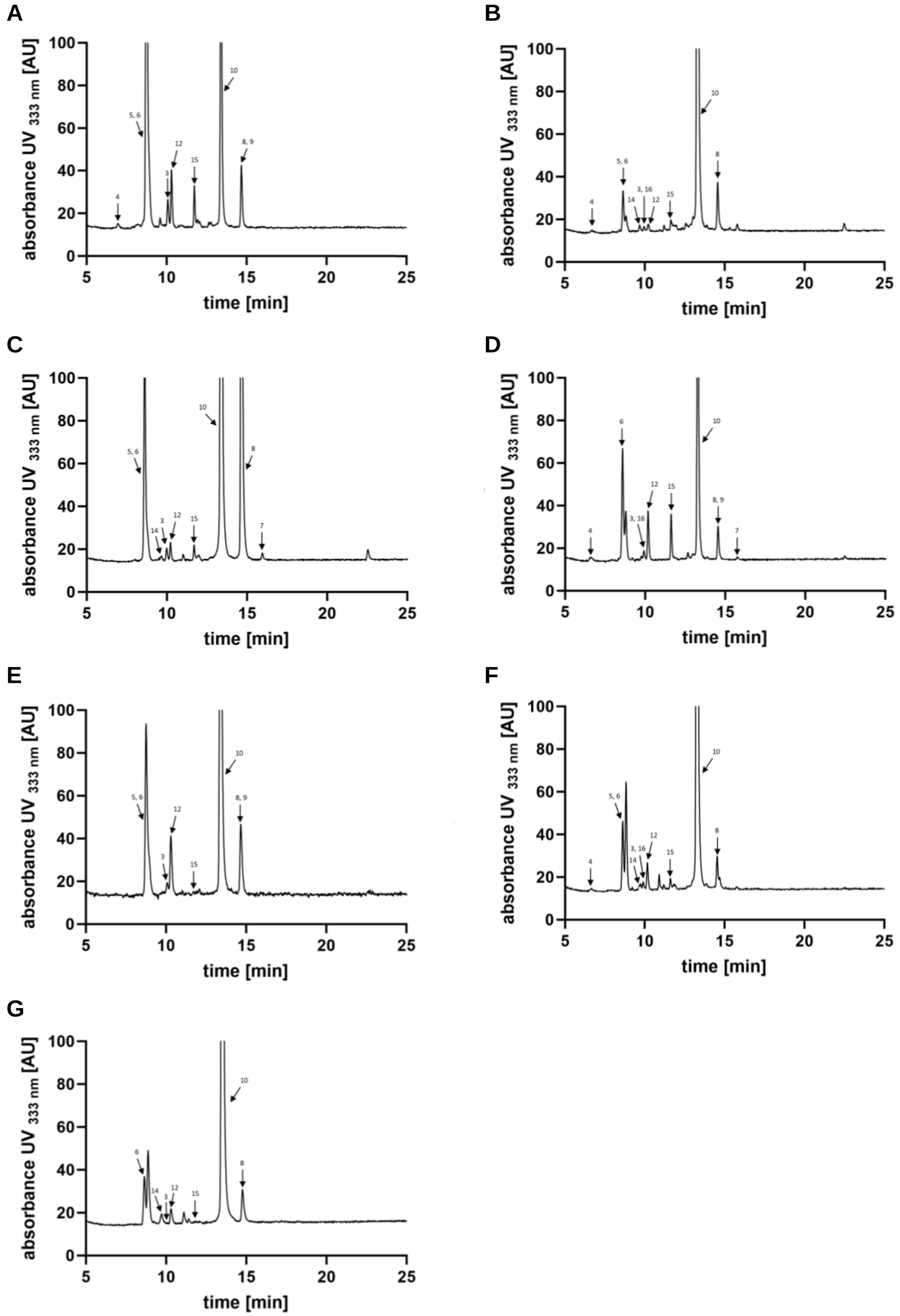

Figure S16. Phytochemical analysis of *Sarcandra glabra*: Exemplary LC-MS chromatograms at 333 nm of replicate 1. Substances were identified with authentical standards and by their

MS spectra (see Table S3). Peaks are labeled according to Table S1. **A**: flowers, **B**: old leaves, **C**: young leaves, **D**: old stems, **E**: young stems, **F**: old roots, **G**: young roots.

Supplementary Tables

Table S1. Hydroxycinnamic acid derivatives (calculated as % of the dry plant mass) in plant parts of *Sarcandra glabra (n* = 3 ± SD); caf = caffeoyl, pC = *p*-coumaroyl, pHPL = 4-hydroxyphenyllactic acid, DHPL = 3,4-dihydroxyphenyllactic acid, * = HPLC peak could not be unambiguously identified by its mass, nd = not detected. For rosmarinic acid glycoside = RA glycoside the used standard is rosmarinic acid 3'-*O*-β-D-glucoside, but the position of the sugar in the compound from *Sarcandra glabra* could not be determined. Note: the peak numbers do not refer to the order of elution. Exemplary LC-MS spectra are displayed in Figure S16.

	**4-coumaric acid**	**caffeic acid**	**pC-5-*O*-shikimic acid**	**caf-5-*O*-shikimic acid**	**pC-3-*O*-quinic acid**	**pC-4-*O*-quinic acid**	**pC-5-*O*-quinic acid**	**caf-3-*O*-quinic acid**	**caf-4-*O*-quinic acid**	**caf-5-*O*-quinic acid**	**pC-pHPL**	**pC-DHPL**	**caf-pHPL**	**RA**	**RA glycoside**	**caf-3-*O*-shikimic acid**	**caf-4-*O*-shikimic acid**
**peak no.**	13	14	11	12	4	5	6	1	2	3	7	9	8	10	15	16	17
**young roots**	nd	0.02 ± 0.001	nd	0.08 ± 0.004	< 0.01	nd	< 0.01	< 0.01	< 0.01	0.26 ± 0.028	nd	0.09 ± 0.021	0.34 ± 0.014	3.08 ± 0.146	< 0.01*	nd	nd
**old roots**	nd	< 0.01	0.02* ± 0.001	0.08 ± 0.003	nd	nd	nd	nd	0.02 ± 0.001	0.22 ± 0.010	nd	0.03* ± 0.002	0.20 ± 0.008	3.60 ± 0.141	0.02* ± 0.003	0.02 ± 0.001	nd
**young stems**	nd	< 0.01	nd	0.31 ± 0.016	nd	nd	0.06 ± 0.008	nd	0.08 ± 0.010	0.87 ± 0.051	0.01 ± 0.003	0.16 ± 0.004	0.80 ± 0.029	2.27 ± 0.164	0.03* ± 0.019	nd	nd
**old stems**	nd	< 0.01	nd	0.31 ± 0.125	nd	nd	0.02 ± 0.005	< 0.01	0.03* ± 0.006	0.38 ± 0.056	< 0.01	0.07 ± 0.018	0.27 ± 0.040	1.10 ± 0.197	0.11* ± 0.027	0.03 ± 0.004	nd
**young leaves**	nd	< 0.01	nd	0.06 ± 0.005	nd	nd	0.05 ± 0.002	nd	0.05 ± 0.002	0.85 ± 0.033	0.07 ± 0.015	0.21* ± 0.007	6.14 ± 0.068	3.53 ± 0.054	0.05* ± 0.008	nd	nd
**old leaves**	nd	0.01 ± 0.001	0.01* ± 0.002	0.03 ± 0.004	nd	nd	< 0.01	< 0.01	0.03 ± 0.001	0.16 ± 0.002	nd	0.09* ± 0.020	0.46 ± 0.005	3.36 ± 0.045	0.04* ± 0.004	< 0.01	nd
**flowers**	< 0.01	< 0.01	nd	0.19 ± 0.001	nd	< 0.01	0.11 ± 0.000	0.02 ± 0.004	0.11 ± 0.007	1.99 ± 0.031	nd	0.04 ± 0.008	0.49 ± 0.019	1.01 ± 0.108	0.16* ± 0.031	nd	nd

Table S2. Compounds in *Sarcandra glabra* identified by HPLC, compared with authentic standards. nd: not determined as the peaks were below the limit of detection.

Peak no.	Retention time compound [min]	Absorbance maximum (λ_{max}) [nm]	Proposed compound	Authentic standard	Retention time standard [min]	Absorbance maximum (λ_{max}) [nm]
1	3.0	324	caffeoyl-3-*O*-quinic acid = neochloro-genic acid	caffeoyl-3-*O*-quinic acid = neochloro-genic acid	2.9	325
2	3.9-4.0	324-327	caffeoyl-4-*O*-quinic acid = cryptochloro-genic acid	caffeoyl-4-*O*-quinic acid = cryptochloro-genic acid	3.9	326
3	4.6-4.7	326-327	caffeoyl-5-*O*-quinic acid = chlorogenic acid	caffeoyl-5-*O*-quinic acid = chlorogenic acid	4.6	326
4	3.4	nd	*p*-coumaroyl-3-*O*-quinic acid	*p*-coumaroyl-3-*O*-quinic acid	3.5	311
5	5.3	nd	*p*-coumaroyl-4-*O*-quinic acid	*p*-coumaroyl-4-*O*-quinic acid	5.2	311
6	6.7-6.9	311	*p*-coumaroyl-5-*O*-quinic acid	*p*-coumaroyl-5-*O*-quinic acid	6.7	311
7	54.4-57.8	nd	*p*-coumaroyl-4’-hydroxy-phenyllactic acid	*p*-coumaroyl-4’-hydroxy-phenyllactic acid	55.5	313
8	32.5-34.5	327	caffeoyl-4’-hydroxy-phenyllactic acid	caffeoyl-4’-hydroxy-phenyllactic acid	32.9	328
9	28.6-30.0	nd	*p*-coumaroyl-3’,4’-dihydro-xyphenyllactic acid	*p*-coumaroyl-3’,4’-dihydro-xyphenyllactic acid	29.1	313
10	17.6-18.4	328	caffeoyl-3’,4’-dihydroxyphe-nyllactic acid = rosmarinic acid	caffeoyl-3’,4’-dihydroxyphe-nyllactic acid = rosmarinic acid	17.8	328
11	13.0	nd	*p*-coumaroyl-5-*O*-shikimic acid	*p*-coumaroyl-5-*O*-shikimic acid	12.9	312

12	7.7-7.8	327	caffeoyl-5-*O*-shikimic acid	caffeoyl-5-*O*-shikimic acid	7.5-7.8	326-327
13	8.7-8.9	nd	4-coumaric acid	4-coumaric acid	8.7	309
14	5.6-5.7	325	caffeic acid	caffeic acid	5.6	323
15	13.5-14.0	326	rosmarinic acid glycoside	rosmarinic acid 3'-*O*-β-D-glucoside	13.6	325-332
16	6.7	nd	caffeoyl-3-*O*-shikimic acid	caffeoyl-3-*O*-shikimic acid	6.7	326
17	5.3	323	caffeoyl-4-*O*-shikimic acid	caffeoyl-4-*O*-shikimic acid	5.2-5.3	326

Table S3. Compounds in *Sarcandra glabra* identified by LC-MS analysis, compared with authentic standards. Peak numbers marked with an asterisk were not distinguishable in LC-MS analysis because of very close retention times or overlap of two peaks and MS/MS data may come from both substances; in this case, MS2 is written in italics. nd: not detected or below limit of detection.

Peak no.	Retention time compound [min]	Absorbance maximum (λ_{max}) [nm]	Measured $[M-H]^-$ (*m/z*)	LC/ESI-MS/MS *m/z* (% base peak, only >1%)	Proposed substance	Molecular formula	Authentic standard	Retention time authentic standard [min]	Absorbance maximum (λ_{max}) [nm]	Measured $[M-H]^-$ (*m/z*) (calculated ± 0.05)	LC/ESI-MS/MS *m/z* (% base peak, only >1%)
1	6.7	nd	353.0973	nd	caffeoyl-3-*O*-quinic acid = neochlorogenic acid	$C_{16}H_{18}O_9$	caffeoyl-3-*O*-quinic acid = neochloro-genic acid	6.5	326	353.0968 (353.0867)	MS2 [353]: 135 (25), 161 (3), 173 (4), 179 (51), 191 (100)
2*	8.6	324-328	353.0958	*MS2 [353]: 132 (2), 161 (2), 179 (2), 191 (100), 192 (9), 193 (2)*	caffeoyl-4-*O*-quinic acid = cryptochloro-genic acid	$C_{16}H_{18}O_9$	caffeoyl-4-*O*-quinic acid = cryptochlo-rogenic acid	8.7	326	353.0984 (353.0867)	MS2 [353]: 133 (5), 134 (4), 135 (52), 155 (2), 161 (8), 173 (72), 174 (6), 179 (60), 180 (5), 191 (100), 192 (8), 193 (2)
3*	8.6	326	353.0958	*MS2 [353]: 132 (2), 161 (2), 179 (2), 191 (100), 192 (9), 193 (2)*	caffeoyl-5-*O*-quinic acid = chlorogenic acid	$C_{16}H_{18}O_9$	caffeoyl-5-*O*-quinic acid = chlorogenic acid	8.6	326	353.0982 (353.0867)	MS2 [353]: 161 (2), 191 (100), 192 (7), 193 (2)
4*	nd	nd	nd	nd	*p*-coumaroyl-3-*O*-quinic acid	$C_{16}H_{18}O_8$	*p*-coumaroyl-3-*O*-quinic acid	8.1	310	337.1024 (337.0918)	MS2 [337]: 119 (64), 155 (8), 163 (100), 173 (4), 191 (20)
5*	10.1	314	337.1057	*MS2 [337]: 111 (9), 119 (9), 135 (5), 163 (9), 173 (13), 179 (2), 191 (100), 192 (7), 193 (2), 261 (2)*	*p*-coumaroyl-4-*O*-quinic acid	$C_{16}H_{18}O_8$	*p*-coumaroyl-4-*O*-quinic acid	10.0	312	337.1020 (337.0918)	MS2 [337]: 111 (4), 119 (11), 137 (5), 155 (4), 163 (22), 164 (2), 173 (100), 174 (9), 175 (2), 191 (6),
6*	10.1	314	337.1057	*MS2 [337]: 111 (9), 119 (9), 135 (5), 163 (9), 173 (13),*	*p*-coumaroyl-5-*O*-quinic acid	$C_{16}H_{18}O_8$	*p*-coumaroyl-5-*O*-quinic acid	10.0	312	337.1022 (337.0918)	MS2 [337]: 119 (4), 163 (17), 173 (5), 191 (100), 192 (8)

Peak no.	Retention time compound [min]	Absorbance maximum (λ_{max}) [nm]	Measured [M-H]$^-$ (*m/z*)	LC/ESI-MS/MS *m/z* (% base peak, only >1%)	Proposed substance	Molecular formula	Authentic standard	Retention time authentic standard [min]	Absorbance maximum (λ_{max}) [nm]	Measured [M-H]$^-$ (*m/z*) (calculated ± 0.05)	LC/ESI-MS/MS *m/z* (% base peak, only >1%)
				179 (2), 191 (100), 192 (7), 193 (2), 261 (2)							
7	16.0	nd	327.0915	nd	*p*-coumaroyl-4'-hydroxyphenyl-lactic acid	$C_{18}H_{16}O_6$	*p*-coumaroyl-4'-hydroxy-phenyllactic acid	15.9	312	327.0959 (327.0863)	MS2 [327]: 117 (17), 119 (16), 135 (27), 145 (89), 163 (63), 181 (100), 216 (2)
8*	14.6	325	343.0906	*MS2 [343]: 116 (2), 117 (2), 119 (6), 121 (2), 122 (3), 132 (6), 133 (20), 134 (9), 135 (15), 145 (2), 161 (100), 162 (15), 163 (47), 164 (2), 177 (2), 179 (8), 181 (72), 182 (7), 192 (3), 197 (3), 229 (2)*	caffeoyl-4'-hydroxyphenyl-lactic acid = isorinic acid	$C_{18}H_{16}O_7$	caffeoyl-4'-hydroxy-phenyllactic acid = isorinic acid	14.6	328	343.0912 (343.0812)	MS2 [343]: 117 (2), 119 (5), 132 (6), 133 (17), 134 (2), 135 (23), 136 (2), 145 (3), 161 (100), 162 (9), 163 (33), 164 (3), 176 (6), 181 (64), 182 (3)
9*	14.6	325	343.0906	*MS2 [343]: 116 (2), 117 (2), 119 (6), 121 (2), 122 (3), 132 (6), 133 (20), 134 (9), 135 (15), 145 (2), 161 (100), 162 (15), 163 (47), 164 (2), 177 (2), 179 (8), 181 (72), 182 (7), 192 (3), 197 (3), 229 (2)*	*p*-coumaroyl-3',4'-dihydroxy-phenyllactic acid	$C_{18}H_{16}O_7$	*p*-coumaroyl-3',4'-dihydro-xyphenyllac-tic acid	14.5	314	343.0919 (343.0812)	MS2 [343]: 117 (30), 123 (16), 135 (76), 145 (100), 179 (80), 197 (52)

Peak no.	Retention time compound [min]	Absorbance maximum (λ_{max}) [nm]	Measured [M-H]$^-$ (*m/z*)	LC/ESI-MS/MS *m/z* (% base peak, only >1%)	Proposed substance	Molecular formula	Authentic standard	Retention time authentic standard [min]	Absorbance maximum (λ_{max}) [nm]	Measured [M-H]$^-$ (*m/z*) (calculated ± 0.05)	LC/ESI-MS/MS *m/z* (% base peak, only >1%)
10	13.2	328	359.0858	MS2 [359]: 123 (7), 132 (5), 133 (10), 134 (3), 135 (17), 161 (100), 162 (8), 179 (37), 197 (26), 198 (2)	caffeoyl-3’,4’-dihydroxyphe-nyllactic acid = rosmarinic acid	$C_{18}H_{16}O_8$	caffeoyl-3’,4’-dihydro-xyphenyllac-tic acid = rosmarinic acid	13.3	328	359.0873 (359.0761)	MS2 [359]: 123 (8), 132 (4), 133 (11), 135 (11), 161 (100), 162 (8), 179 (37), 180 (3), 197 (29), 198 (3)
11	11.6	nd	319.0920	nd	*p*-coumaroyl-5-*O*-shikimic acid	$C_{16}H_{16}O_7$	*p*-coumaroyl-5-*O*-shikimic acid	11.5	312	319.0895 (319.0812)	MS2 [319]: 117 (15), 119 (100), 137 (18), 145 (24), 161 (4), 163 (52), 173 (6), 185 (2), 211 (2), 215 (2), 239 (2), 275 (3)
12	10.0	323	335.0843	MS2 [335]: 125 (3), 132 (6), 133 (16), 135 (100), 136 (12), 137 (5), 138 (3), 145 (2), 155 (5), 160 (2), 161 (51), 179 (99), 180 (8), 227 (5), 228 (4), 245 (3), 251 (3), 259 (2), 278 (3)	caffeoyl-5-*O*-shikimic acid	$C_{16}H_{16}O_8$	caffeoyl-5-*O*-shikimic acid	10.0	328	335.0900 (335.0761)	MS2 [335]: 111 (3), 115 (2), 132 (2), 133 (5), 134 (5), 135 (73), 136 (6), 137 (2), 161 (33), 162 (9), 173 (4), 179 (100), 180 (8)
13	11.5	nd	163.0448	nd	4-coumaric acid	$C_9H_8O_3$	4-coumaric acid	11.4	310	163.0417 (163.0390)	MS2 [163]: 117 (5), 119 (100), 145 (2)
14	9.7	nd	179.0404	nd	caffeic acid	$C_9H_8O_4$	caffeic acid	9.6	322	179.0376 (179.0339)	MS2 [179]: 117 (5), 133 (7), 134 (64), 135 (100), 136 (8)
15	11.7	330	521.1452	MS2 [521]: 123 (7), 132 (3), 133 (12), 134 (4), 135 (8),	rosmarinic acid glycoside	$C_{24}H_{26}O_{13}$	rosmarinic acid 3’-O-β-D-glucoside	12.1	330	521.1520 (521.1290)	MS2 [521]: 123 (3), 135 (7), 161 (3), 179 (4), 197 (46), 198 (5), 359 (100), 360 (13), 361 (2)

Peak no.	Retention time compound [min]	Absorbance maximum (λ_{max}) [nm]	Measured $[M-H]^-$ (*m/z*)	LC/ESI-MS/MS *m/z* (% base peak, only >1%)	Proposed substance	Molecular formula	Authentic standard	Retention time authentic standard [min]	Absorbance maximum (λ_{max}) [nm]	Measured $[M-H]^-$ (*m/z*) (calculated ± 0.05)	LC/ESI-MS/MS *m/z* (% base peak, only >1%)
				161 (82), 162 (4), 179 (17), 197 (100), 198 (8), 341 (2), 359 (65), 360 (9), 361 (3)							
16	9.7	nd	335.0881	nd	caffeoyl-3-*O*-shikimic acid	$C_{16}H_{16}O_8$	caffeoyl-3-*O*-shikimic acid	9.7	328	335.0896 (335.0761)	MS2 [335]: 104 (2), 109 (3), 111 (5), 132 (3), 133 (18), 134 (16), 135 (95), 136 (2), 137 (29), 138 (2), 139 (3), 155 (3), 159 (2), 161 (13), 162 (2), 163 (2), 173 (6), 178 (6), 179 (100), 180 (12), 204 (2), 210 (3)
17	9.5	nd	335.0891	nd	caffeoyl-4-*O*-shikimic acid	$C_{16}H_{16}O_8$	caffeoyl-4-*O*-shikimic acid	9.5	326	335.0892 (335.0761)	MS2 [335]: 132 (6), 133 (18), 134 (4), 135 (12), 137 (2), 161 (100), 162 (10), 179 (10)

Table S4. BLASTP results using the amino acid sequence of rosmarinic acid synthase (RAS) from *Coleus blumei* (A0PDV5, Berger *et al.* (2006)) as bait sequence in the transcriptome of *Sarcandra glabra* (1kP database, taxid:92927, OSHQ). Note: alignment length does not refer to the scaffold length; aa = amino acids. Cutoff value: e-value > $1.0 \cdot 10^{-50}$ **A** List of scaffolds leading to enzymes covered in this report (e-value <$1.0 \cdot 10^{-50}$) **B** List of scaffolds leading to other putative BAHDs (e-value >$1.0 \cdot 10^{-50}$). 'Properties' gives short information about the sequence beginning with a start-methionine (putative full-length) or not (partial), annotations refer to BLAST similarities and the occurrence of the typically conserved motifs HxxxDG and DFGWG.

A

Description	E-value	Identity [%]	Alignment length [aa]	Preliminary name	Enzyme and GenBank accession
OSHQ_scaffold_2009492	$3.29 \cdot 10^{-163}$	52	444	SgHCT-A	SgHST, PP449349
OSHQ_scaffold_2009493	$1.30 \cdot 10^{-157}$	52	436	SgHCT-B	considered incorrect
OSHQ_scaffold_2009494	$5.35 \cdot 10^{-145}$	50	436	SgHCT-C	SgHQT1, PP449350
OSHQ_scaffold_2048693	$5.47 \cdot 10^{-117}$	43	451	SgHCT-D	SgRAS, PP449351
OSHQ_scaffold_2009853	$9.33 \cdot 10^{-87}$	44	304	SgHCT-E	SgHQT2, PP449352
OSHQ_scaffold_2048698	$4.04 \cdot 10^{-53}$	30	437	SgHCT-F	PQ336776

B

Description	E-value	Identity [%]	Alignment length [aa]	Properties
OSHQ_scaffold_2008674	$3.53 \cdot 10^{-32}$	27	451	Full-length, putative BAHD, HxxxDG and DFGWG
OSHQ_scaffold_2048702	$5.03 \cdot 10^{-32}$	30	393	Full-length, putative spermidine HCT, HxxxDG and DFGWG
OSHQ_scaffold_2007962	$4.43 \cdot 10^{-31}$	28	393	Partial, putative malonyl-CoA transferase, HxxxDG and DFGWG
OSHQ_scaffold_2008889	$5.28 \cdot 10^{-28}$	28	454	Full-length, putative malonyl-CoA transferase, HxxxDG and DFGWG
OSHQ_scaffold_2008890	$1.57 \cdot 10^{-27}$	29	399	Full-length, putative malonyl-CoA transferase, HxxxDG and DFGWG
OSHQ_scaffold_2002086	$1.27 \cdot 10^{-25}$	28	384	Partial, putative BAHD, HxxxDG and DFGWG
OSHQ_scaffold_2007961	$8.69 \cdot 10^{-25}$	46	348	Partial, putative malonyl-CoA transferase, HxxxDG and DFGWG
OSHQ_scaffold_2044169	$1.18 \cdot 10^{-23}$	28	113	Partial, putative BAHD, front part of SgHQT2
OSHQ_scaffold_2000049	$1.61 \cdot 10^{-19}$	28	367	Partial, putative malonyl-CoA transferase, DFGWG
OSHQ_scaffold_2002085	$1.20 \cdot 10^{-18}$	28	348	Partial, putative BAHD, HxxxDG and DFGWG

OSHQ_scaffold_2008047	$1.89\cdot10^{-18}$	26	318	Partial, putative HCT or ECERIFERUM 26-like
OSHQ_scaffold_2010877	$3.44\cdot10^{-18}$	27	400	Partial, putative malonyl-CoA transferase, HxxxDG and DFGWG
OSHQ_scaffold_2005020	$4.27\cdot10^{-18}$	25	462	Partial, putative malonyl-CoA transferase, DFGWG
OSHQ_scaffold_2009048	$6.88\cdot10^{-18}$	25	397	Partial, putative malonyl-CoA transferase, HxxxDG and DFGWG
OSHQ_scaffold_2005299	$4.38\cdot10^{-12}$	32	145	Partial, HxxxDG
OSHQ_scaffold_2005298	$4.33\cdot10^{-11}$	32	140	Partial, HxxxDG
OSHQ_scaffold_2000050	$1.06\cdot10^{-10}$	26	312	Partial, DFGWG
OSHQ_scaffold_2044504	$1.06\cdot10^{-08}$	27	119	Partial, HxxxDG
OSHQ_scaffold_2004393	$5.48\cdot10^{-08}$	23	298	Partial, putative BAHD, HxxxDG and DFGWG
OSHQ_scaffold_2009217	$3.42\cdot10^{-07}$	29	145	
OSHQ_scaffold_2043089	$2.12\cdot10^{-05}$	40	56	Partial, DFGWG
OSHQ_scaffold_2005742	$3.67\cdot10^{-05}$	26	136	Partial, HxxxDG
OSHQ_scaffold_2002666	$5.44\cdot10^{-05}$	27	100	
OSHQ_scaffold_2041947	0.11	36	54	Partial, DFGWG
OSHQ_scaffold_2007680	1.0	29	78	
OSHQ_scaffold_2048236	1.2	33	79	
OSHQ_scaffold_2004392	1.7	23	289	Partial, DFGWG
OSHQ_scaffold_2045088	2.4	25	153	Partial, DFGWG
OSHQ_scaffold_2007865	2.8	32	80	
OSHQ_scaffold_2004803	3.0	30	44	
OSHQ_scaffold_2004801	3.0	30	44	
OSHQ_scaffold_2004800	3.0	30	44	
OSHQ_scaffold_2010873	3.5	29	69	
OSHQ_scaffold_2048860	5.0	22	109	
OSHQ_scaffold_2008080	5.1	37	47	
OSHQ_scaffold_2046848	7.2	36	37	
OSHQ_scaffold_2048655	7.3	30	58	
OSHQ_scaffold_2011324	7.4	40	43	
OSHQ_scaffold_2047975	9.0	41	32	

Table S5. Pairwise comparison (EMBOSS Needle) of amino acid sequences of identified hydroxycinnamoyltransferases from *Sarcandra glabra.*

	SgHST	SgHQT1	SgRAS	SgHQT2	SgHCT-F
**SgHST**	100/100	69.6/83.1	43.8/63.1	53.3/69.3	48.0/23.6
**SgHQT1**		100/100	41.9/62.1	53.2/69.6	28.4/45.2
**SgRAS**			100/100	42.0/58.0	27.8/44.4
**SgHQT2**				100/100	28.2/45.8
**SgHCT-F**					100/100

Table S6. Abbreviations for species names used in the phylogenetic tree (Figure S3). Accession numbers were taken from Genbank, Uniprot and Phytozome. If sequences were not available in these data collections, scaffolds from transcriptome sequencing were taken. Proven substrates are abbreviated as A (anthranilic acid, hydroxyanthranilic acid), Ag (agmatine), B (benzyl alcohol), F (fatty acid derivatives), G (glycerol), La (L-amino acids) M (malic acid), P (4-hydroxyphenyllactic acid, 3,4-dihydroxyphenyllactic acid), Pi (piscidic acid), Pt (pseudotropine), Q (quinic acid), S (shikimic acid), Sp (spermidine, spermine), + (and additional substrates). Substrates shown in brackets are not experimentally verified.

Abbreviation	Species and family	Accession_name_substrate
Actrac	*Actaea racemosa* (Ranunculaceae)	QAA12830_ActracHPT1_Pi
Antagr	*Anthoceros agrestis* (Anthocerotaceae)	MW248389_AntagrHCT6_SA
Aratha	*Arabidopsis thaliana* (Brassicaceae)	NP_179497_ArathaSHT_Sp
Atrbel	*Atropa belladonna* (Solanaceae)	WPF47615_AtrbelTS_Pt
Avesat	*Avena sativa* (Poaceae)	AB076980_AvesatHHT1_A
Bammul	*Bambusa multiplex* (Poaceae)	BCY27076_BammulHQT1_Q<br>BDR61289_BammulHCT1_S
Canind	*Canna indica* (Cannaceae)	2022568_CanindHCT_B<br>(own unpublished results)
Cartin	*Carthamus tinctorius* (Asteraceae)	scaffold283946_CartinHCT_SQ
Cicint	*Cichorium intybus* (Asteraceae)	KT222891_CicintHCT1_SQ<br>KT222892_CicintHCT2_SQ<br>KT222893_CicintHQT1_QS<br>KT222894_CicintHQT2_QS<br>KT222895_CicintHQT3_QS<br>MG457243_CicintSHT1_Sp<br>MG457244_CicintSHT2_Sp
Clabre	*Clarkia breweri* (Onagraceae)	AF500200_ClabreBBT_B
Cofcan	*Coffea canephora* (Rubiaceae)	EF137954_CofcanHCT_SQ<br>EF153931_CofcanHQT_Q
Cyncar	*Cynara cardunculus* (Asteraceae)	EU839580_CyncarHQT2_QS
Diacar	*Dianthus caryophyllus* (Caryophyllaceae)	Z84386_DiacarHCBT2_A
Echpur	*Echinacea purpurea* (Asteraceae)	MT936805_EchpurHCT_SQ
Erycoc	*Erythroxylum coca* (Erythroxylaceae)	JQ413187_ErycocHQT_(Q)
Foevul	*Foeniculum vulgare* (Apiaceae)	FoevulHCT1_S<br>FoevulHCT2_S<br>(own unpublished results)
Horvul	*Hordeum vulgare* (Poaceae)	BAF97626_HorvulACT1-1_Ag
Lavang	*Lavandula angustifolia* (Lamiaceae)	DQ886904_LavangAAT1_P
Marema	*Marchantia emarginata* (Marchantiaceae)	Marema_MeHFT_F<br>(Wang *et al.*, 2017)
Marpal	*Marchantia paleacea* (Marchantiaceae)	AXN55971_MarpalHCT_SQ
Marpol	*Marchantia polymorpha* (Marchantiaceae)	OAE34410_MarpolHCT
Meloff	*Melissa officinalis* (Lamiaceae)	FR670523_MeloffRAS_P

Abbreviation	Species and family	Accession_name_substrate
Menlon	*Mentha longifolia* (Lamiaceae)	MenlonAT1_SPQ<br>MenlonAT2_S(P)<br>MenlonAT4_SP<br>MenlonAT6_SPQ<br>(Zhou *et al.*, 2024)
Nictab	*Nicotiana tabacum* (Solanaceae)	AF500202_NictabBBT_B<br>AJ507825_NictabHCT_S<br>AJ582651_NictabHQT_Q<br>MN787045_NictabSHT_(Sp)
Orysat	*Oryza sativa* (Poaceae)	XM_015786263_OrysatHCT4_SG
Panvir	*Panicum virgatum* (Poaceae)	AB723827_PanvirHCT1a_(S)<br>AFY17066_PanvirHCT-like1<br>KC696573_PanvirHCT2a_SQ
Phacam	*Phacelia campanularia* (Boraginaceae)	MH878831_PhacamHST_S<br>MH878832_PhacamHQT_Q<br>MH878833_PhacamRAS_P<br>MH878834_PhacamSHT_Sp
Phavul	*Phaseolus vulgaris* (Fabaceae)	KX443573_PhavulHHHT_H
Plaapp	*Plagiochasma appendiculatum* (Aytoniaceae)	AXN55972_PlaappHCT_SQ
Pinrad	*Pinus radiata* (Pinaceae)	EF121452_PinradHCT_S
Plescu	*Plectranthus scutellarioides* (Lamiaceae)	CAK55166_PlescuRAS_P<br>FN647681_PlescuHCT2_S
Poptri	*Populus trichocarpa* (Salicaceae)	EU603313_PoptriHCT1_(QS)<br>KP228019_PoptriBBT_B<br>XM_006368430_PoptriHCT6_(QS)
Pruper	*Prunus persica* (Rosaceae)	XP_007215395_PruperHCT4<br>XP_007215396_PruperHCT5
Riccom	*Ricinus communis* (Euphorbiaceae)	MN787043_RiccomAHT_(A)
Roscan	*Rosa canina* (Rosaceae)	MN787046_RoscanSHT_(Sp)
Salmil	*Salvia miltiorrhiza* (Lamiaceae)	ADA60182_SalmilRAS_(P)
Sargla	*Sarcandra glabra* (Chloranthaceae)	PP449349_SarglaHST_SAQ+<br>PP449350_SarglaHQT1_QSA+<br>PP449351_SarglaRAS_P<br>PP449352_SarglaHQT2_Q(S)+<br>PQ336776_SarglaHCT_F_(B)
Selmoe	*Selaginella moellendorffii* (Selaginellaceae)	XM_002979015_SelmoeHCT1a_SA+
Sollyc	*Solanum lycopersicum* (Solanaceae)	AJ582652_SollycHQT_Q
Sorbic	*Sorghum bicolor* (Poaceae)	XM_002452390_SorbicHCT_SQ
Tripra	*Trifolium pratense* (Fabaceae)	AXB87812_TripraHDT1_La<br>EU861218_TripraHCT1A_S<br>EU861219_TripraHCT2_M<br>FJ151489_TripraHCT1B_S
Vitvin	*Vitis vinifera* (Vitaceae)	MN787047_VitvinSHT_(Sp)

Table S7. LC-MS analysis of enzyme assays with SgHST. The list shows the acceptor and donor substrate as well as the detected product with its retention time, molecular formula and detected $[M-H]^-$ (*m*/*z*). The position of the attachment of the hydroxycinnamoyl unit is only given, when a respective authentic standard could be used.

Acceptor	Donor	Product	Retention time [min]	Molecular formula	Detected $[M-H]^-$ (*m*/*z*)
shikimic acid	cinnamoyl-CoA	cinnamoylshikimic acid	8.9	$C_{16}H_{16}O_6$	303.09
shikimic acid	*p*-coumaroyl-CoA	*p*-coumaroyl-5-*O*-shikimic acid	7.7	$C_{16}H_{16}O_7$	319.08
shikimic acid	caffeoyl-CoA	caffeoyl-5-*O*-shikimic acid	7.2	$C_{16}H_{16}O_8$	335.08
shikimic acid	feruloyl-CoA	feruloylshikimic acid	7.9	$C_{17}H_{18}O_8$	349.09
shikimic acid	sinapoyl-CoA	sinapoylshikimic acid	7.7	$C_{18}H_{20}O_9$	379.11
3-hydroxyanthra-nilic acid	*p*-coumaroyl-CoA	*p*-coumaroyl-2-*N*-3-hydroxyanthranilic acid	9.9	$C_{16}H_{13}O_5N_1$	299.08
3-hydroxybenzoic acid	*p*-coumaroyl-CoA	*p*-coumaroylhydroxy-benzoic acid	10.0	$C_{16}H_{12}O_5$	283.06
2,3-dihydroxy-benzoic acid	*p*-coumaroyl-CoA	*p*-coumaroyl-2,3-di-hydroxybenzoic acid	10.2	$C_{16}H_{12}O_6$	299.06
2,5-dihydroxy-benzoic acid	*p*-coumaroyl-CoA	*p*-coumaroyl-2,5-di-hydroxybenzoic acid	10.2	$C_{16}H_{12}O_6$	299.06
3,4-dihydroxy-benzoic acid	*p*-coumaroyl-CoA	*p*-coumaroyl-3,4-di-hydroxybenzoic acid	8.9	$C_{16}H_{12}O_6$	299.06
3-aminobenzoic acid	*p*-coumaroyl-CoA	*p*-coumaroylamino-benzoic acid	9.1	$C_{16}H_{13}O_4N_1$	282.06
quinic acid	*p*-coumaroyl-CoA	*p*-coumaroyl-5-*O*-quinic acid	7.1	$C_{16}H_{18}O_8$	337.09
5-hydroxyanthra-nilic acid	*p*-coumaroyl-CoA	*p*-coumaroyl-5-hydro-xyanthranilic acid	9.7	$C_{16}H_{13}O_5N_1$	299.08

Table S8. Tested putative acceptor substrates for all SgHCTs. Assays were performed in 0.1 M K_2HPO_4/KH_2PO_4 pH 7.0 with 200 µM *p*-coumaroyl-CoA, 1 to 5 µg purified enzyme and 0.8 to 8 mM acceptor substrate (depending on solubility). In case of SgHCT-F, 16-hydroxypalmitic acid, benzyl alcohol, 2- and 4-hydroxybenzyl alcohol were also tested with 200 µM benzoyl-CoA. The incubation took place at 30 °C for up to 3 h. A: accepted, N: not accepted, empty cell: not tested.

SgHST	SgHQT1	SgHQT2	SgRAS	SgHCT-F	Substrate
				N	16-hydroxypalmitic acid
N		N	N	N	1-butanol
N			N	N	1-phenylethanol
N	N	N	N	N	1-propanol
N			N	N	2-(4-hydroxyphenyl)-1-ethanol
A	A	N	N	N	2,3-dihydroxybenzoic acid
N	A	A	N	N	2,4-dihydroxybenzoic acid

SgHST	SgHQT1	SgHQT2	SgRAS	SgHCT-F	Substrate
A	A	N	N	N	2,5-dihydroxybenzoic acid (gentisic acid)
N	N	N	N	N	2-hydroxybenzoic acid (salicylic acid)
				N	2-hydroxybenzyl alcohol
N			N	N	2-phenethylamine
N			N	N	2-phenylethanol
N	N	N	N	N	2-propanol
N			N	N	3-(4-hydroxyphenyl)-1-propanol
A	A	N	N	N	3,4-dihydroxybenzoic acid (protocatechuic acid)
N	N	N	N	N	3-amino-2-hydroxybenzoic acid (3-aminosalicylic acid)
A	A	N	N	N	3-aminobenzoic acid
A	A	N	N	N	3-hydroxyanthranilic acid
A	N	N	N	N	3-hydroxybenzoic acid (*meta*-salicylic acid)
N			N	N	3-phenyl-1-propanol
N	A	A	N	N	4-hydroxybenzoic acid
			N	N	4-hydroxybenzyl alcohol
				N	4-isopropylbenzyl alcohol
N			N	N	4-phenyl-1-butanol
A	A	N			5-hydroxyanthranilic acid
N			N	N	agmatine
N	N	N	N	N	anthranilic acid
N	N	N	N	N	benzoic acid
	N	N	N	N	benzyl alcohol
N			N	N	catechin
N	N	N	N	N	chlorogenic acid
			N	N	coniferyl alcohol
N			A	N	D/L-3,4-dihydroxyphenyllactic acid
N			A	N	D/L-4-hydroxy-3-methoxyphenyllactic acid
N	N		A	N	D/L-4-hydroxyphenyllactic acid
N			A	N	D/L-dihydroxyphenylalanine
N			N	N	D/L-glutamic acid
N	N	N	N	N	D/L-malic acid
			A	N	D/L-phenyllactic acid
N			N	N	D-malic acid
				N	dodecanol
N			N	N	dopamine
N	N	N	A	N	D-phenylalanine
N	N		N	N	D-tartaric acid
N			N	N	D-threonic acid
N			N	N	D-tryptophan
N			A	N	D-tyrosine
N	N	N	N	N	ethanol
N			N	N	galactaric acid
N		N	N	N	glucaric acid
	A	N		N	glycerol
N			N	N	L-4-hydroxyphenyllactic acid
N			N	N	L-dihydroxyphenylalanine
N			N	N	L-malic acid

SgHST	SgHQT1	SgHQT2	SgRAS	SgHCT-F	Substrate
N			N	N	L-phenylalanine
			N		L-phenyllactic acid
N	N		N	N	L-tartaric acid
N			N		L-threonic acid
N			N	N	L-tryptophan
N			N	N	L-tyrosine
N	A	N	N	N	methanol
N			N	N	*m*-tartaric acid
			N	N	piscidic acid
N		N	N	N	putrescine
A	A	A	N	N	quinic acid
N			N	N	serotonin
A	A	A	N	N	shikimic acid
N	N	N	N	N	spermidine
N		N	N	N	spermine
N			N	N	tryptamine
N			N	N	tyramine
N	N	N	N	N	vanillic acid

Table S9. LC-MS analysis of enzyme assays with SgHQT1. The list shows the acceptor and donor substrate as well as the detected product with its retention time, molecular formula and detected $[M-H]^-$ (*m*/*z*). The position of the attachment of the hydroxycinnamoyl unit is only given, when a respective authentic standard could be used.

Acceptor	Donor	Product	Reten-tion time [min]	Molecular formula	Detected $[M-H]^-$ (*m*/*z*)
quinic acid	cinnamoyl-CoA	cinnamoylquinic acid	8.4	$C_{16}H_{16}O_7$	321.10
quinic acid	*p*-coumaroyl-CoA	*p*-coumaroyl-5-*O*-quinic acid	7.2	$C_{16}H_{18}O_8$	337.09
quinic acid	caffeoyl-CoA	caffeoyl-5-*O*-quinic acid	6.6	$C_{16}H_{16}O_9$	353.09
quinic acid	feruloyl-CoA	feruloylquinic acid	7.3	$C_{17}H_{20}O_9$	367.10
quinic acid	sinapoyl-CoA	sinapoylquinic acid	7.2	$C_{17}H_{22}O_{10}$	397.13
shikimic acid	*p*-coumaroyl-CoA	*p*-coumaroylshikimic acid	7.7	$C_{16}H_{16}O_7$	319.08
glycerol	*p*-coumaroyl-CoA	*p*-coumaroylglycerol	7.5	$C_{12}H_{14}O_5$	238.24
5-hydroxyanthra-nilic acid	*p*-coumaroyl-CoA	*p*-coumaroyl-5-hydroxyanthranilic acid	9.7	$C_{16}H_{13}O_5N_1$	299.08
3-aminobenzoic acid	*p*-coumaroyl-CoA	*p*-coumaroyl-3-amino-benzoic acid	9.2	$C_{16}H_{13}O_4N_1$	282.08
2,3-dihydroxy-benzoic acid	*p*-coumaroyl-CoA	*p*-coumaroyl-2,3-di-hydroxybenzoic acid	10.2	$C_{16}H_{12}O_6$	299.06
2,5-dihydroxy-benzoic acid	*p*-coumaroyl-CoA	*p*-coumaroyl-2,5-di-hydroxybenzouc acid	10.2	$C_{16}H_{12}O_6$	299.06
3,4-dihydroxy-benzoic acid	*p*-coumaroyl-CoA	*p*-coumaroyl-3,4-di-hydroxybenzoic acid	9.1	$C_{16}H_{12}O_6$	299.06
shikimic acid	caffeoyl-CoA	dicaffeoylshikimic acid	8.8	$C_{25}H_{22}O_{11}$	498.12

methanol	*p*-coumaroyl-CoA	*p*-coumaroylmethanol	9.7	$C_{10}H_{10}O_3$	177.05
4-hydroxybenzoic acid	*p*-coumaroyl-CoA	*p*-coumaroyl-4-hydroxybenzoic acid	10.0	$C_{16}H_{12}O_5$	283.06
2,4-dihydroxy-benzoic acid	*p*-coumaroyl-CoA	*p*-coumaroyl-2,4-di-hydroxybenzoic acid	10.0 (λ_{max} = 332 nm)<br>8.9 (λ_{max} > 400 nm)<br>8.1 (λ_{max} = 230 nm)	$C_{16}H_{12}O_6$	299.06
3-hydroxyanthra-nilic acid	*p*-coumaroyl-CoA	*p*-coumaroyl-2-*N*-3-hydroxyanthranilic acid	10.0	$C_{16}H_{13}O_5N_1$	299.08
shikimic acid	caffeoyl-CoA	caffeoyl-3-*O*-shikimic acid,<br>caffeoyl-4-*O*-shikimic acid,<br>caffeoyl-5-*O*-shikimic acid	7.3<br>7.2<br>7.6	$C_{16}H_{16}O_8$	335.08

Table S10. LC-MS analysis of enzyme assays with SgHQT2. The list shows the acceptor and donor substrate as well as the detected product with its retention time, molecular formula and detected $[M-H]^-$ (*m*/*z*). The position of the attachment of the hydroxycinnamoyl unit is only given, when a respective authentic standard could be used.

**Acceptor**	**Donor**	**Product**	**Reten-tion time [min]**	**Molecular formula**	**Detected $[M-H]^-$ (*m*/*z*)**
quinic acid	cinnamoyl-CoA	cinnamoylquinic acid	8.2	$C_{16}H_{16}O_7$	321.10
quinic acid	*p*-coumaroyl-CoA	*p*-coumaroyl-4-*O*-quinic acid	7.0	$C_{16}H_{16}O_8$	337.09
quinic acid	caffeoyl-CoA	caffeoyl-4-*O*-quinic acid	6.6	$C_{16}H_{16}O_9$	353.09
quinic acid	feruloyl-CoA	feruloylquinic acid	7.2	$C_{17}H_{20}O_9$	367.10
quinic acid	sinapoyl-CoA	sinapoylquinic acid	7.2	$C_{17}H_{22}O_{10}$	397.13
4-hydroxybenzoic acid	*p*-coumaroyl-CoA	*p*-coumaroyl-4-hydroxybenzoic acid	9.9	$C_{16}H_{12}O_5$	283.06
2,4-dihydroxy-benzoic acid	*p*-coumaroyl-CoA	*p*-coumaroyl-2,4-hydroxybenzoic acid	9.7	$C_{16}H_{12}O_6$	299.06
shikimic acid	*p*-coumaroyl-CoA	*p*-coumaroylshikimic acid	7.7	$C_{16}H_{16}O_7$	319.08
shikimic acid	caffeoyl-CoA	caffeoyl-4-*O*-shikimic acid	7.1	$C_{16}H_{16}O_8$	335.08

Table S11. LC-MS analysis of enzyme assays with SgRAS. The list shows the acceptor and donor substrate as well as the detected product with its retention time, molecular formula and detected $[M\text{-}H]^-$ (*m*/*z*). The position of the attachment of the hydroxycinnamoyl unit is only given, when a respective authentic standard could be used.

**Acceptor**	**Donor**	**Product**	**Retention time [min]**	**Molecular formula**	**Detected $[M\text{-}H]^-$ (*m*/*z*)**
4-hydroxyphenyllactic acid	cinnamoyl-CoA	cinnamoyl-4'-hydroxyphenylactic acid	10.3	$C_{18}H_{16}O_5$	311.09
4-hydroxyphenyllactic acid	*p*-coumaroyl-CoA	*p*-coumaroyl-4'-hydroxyphenylactic acid	9.2	$C_{18}H_{16}O_6$	327.09
4-hydroxyphenyllactic acid	caffeoyl-CoA	caffeoyl-4'-hydroxyphenylactic acid	8.8	$C_{18}H_{16}O_7$	343.08
4-hydroxyphenyllactic acid	feruloyl-CoA	feruloyl-4'-hydroxyphenylactic acid	9.3	$C_{19}H_{18}O_7$	357.10
4-hydroxyphenyllactic acid	sinapoyl-CoA	sinapoyl-4'-hydroxyphenylactic acid	9.2	$C_{20}H_{20}O_8$	387.12
3-phenyllactic acid	*p*-coumaroyl-CoA	*p*-coumaroylphenyllactic acid	10.4	$C_{18}H_{16}O_5$	311.09
3,4-dihydroxyphenyllactic acid	*p*-coumaroyl-CoA	*p*-coumaroyl-3',4'-dihydroxyphenyllactic acid	8.8	$C_{18}H_{16}O_7$	343.08
4-hydroxy-3-methoxyphenyllactic acid	*p*-coumaroyl-CoA	*p*-coumaroyl-4'-hydroxy-3'-methoxyphenyllactic acid	9.3	$C_{19}H_{18}O_7$	357.10
D-phenylalanine	*p*-coumaroyl-CoA	*p*-coumaroyl-D-phenylalanine	9.3	$C_{18}H_{17}O_4N_1$	310.11
D-tyrosine	*p*-coumaroyl-CoA	*p*-coumaroyl-D-tyrosine	8.4	$C_{18}H_{17}O_5N_1$	326.10
D/L-dihydroxyphenylalanine	*p*-coumaroyl-CoA	*p*-coumaroyl-D-3',4'-dihydroxyphenylalanine	8.0	$C_{18}H_{17}O_6N_1$	342.10

Table S12. Assay compositions in tests with SgHST. Assays generally were performed in a final volume of 125 µl and contained K_2HPO_4/KH_2PO_4 buffer (KP_i), an acyl donor substrate and an acyl acceptor substrate. The tests were started by adding the indicated amount of enzyme and were stopped by adding 20 µl 6 N HCl before extracting the products twice with 0.5 ml ethyl acetate. The collected organic phases were evaporated, and the residue redissolved for HPLC and LC-MS analysis.

**Parameter**		**Conditions**	**Composition**
pH-optimum		25 °C<br>t = 5 min, negative control: t = 0 min<br>*n* = 3	0.1 M KP_i pH 6.00 to 8.50<br>200 µM caffeoyl-CoA<br>400 µM shikimic acid<br>2.88 µg SgHST
Temperature optimum		0 to 60 °C<br>t = 5 min, negative control: t = 0 min<br>*n* = 3	0.1 M KP_i pH 7.5<br>200 µM caffeoyl-CoA<br>400 µM shikimic acid<br>1.44 µg SgHST
**K_m for**	**with**		
p-coumaroyl-CoA	shikimic acid	25 °C<br>t = 6 min, negative control: t = 0 min<br>*n* = 9	0.1 M KP_i pH 7.5<br>2.5-400 µM *p*-coumaroyl-CoA<br>16 mM shikimic acid<br>0.038 µg/0.011 µg SgHST
p-coumaroyl-CoA	quinic acid	25 °C<br>t = 10 min, negative control: t = 0 min<br>*n* = 9	0.1 M KP_i pH 7.5<br>1-200 µM *p*-coumaroyl-CoA<br>160 mM quinic acid pH 7.0<br>0.2 µg SgHST
p-coumaroyl-CoA	3-hydroxy-anthranilic acid	25 °C<br>t = 10 min, negative control: empty vector<br>*n* = 9	0.1 M KP_i pH 7.5<br>0.5-20 µM *p*-coumaroyl-CoA<br>6 mM 3-hydroxyanthranilic acid (in 1 M HCl + 7.96 µl 1 M NaOH)<br>0.075 µg SgHST
caffeoyl-CoA	shikimic acid	25 °C<br>t = 2.5 min, negative control: t = 0 min<br>*n* = 9	0.1 M KP_i pH 7.5<br>30 mM shikimic acid<br>5-150 µM caffeoyl-CoA<br>0.32 µg SgHST
shikimic acid	*p*-coumaroyl-CoA	25 °C<br>t = 10 min, negative control: t = 0 min<br>*n* = 9	0.1 M KP_i pH 7.5<br>200 µM *p*-coumaroyl-CoA<br>0.125-12 mM shikimic acid<br>0.03 µg SgHST
shikimic acid	caffeoyl-CoA	25 °C<br>t = 2.5 min, negative control: t = 0 min<br>*n* = 9	0,1 M KP_i pH 7.5<br>300 µM caffeoyl-CoA<br>1.25-40 mM shikimic acid<br>0.32 µg SgHST
quinic acid	*p*-coumaroyl-CoA	25 °C<br>t = 10 min, negative control: t = 0 min<br>*n* = 9	0.1 M KP_i pH 7.5<br>200 µM *p*-coumaroyl-CoA<br>2-160 mM quinic acid pH 7.0<br>0.33/1.0 µg SgHST
3-hydroxy-anthranilic acid	*p*-coumaroyl-CoA	25 °C<br>t = 30 min, negative control: empty vector<br>*n* = 9	0.1 M KP_i pH 7.5<br>80 µM *p*-coumaroyl-CoA<br>0.125-12 mM 3-hydroxy-anthranilic acid (in 1 M HCl + 15.93 µl 1 M NaOH)<br>0.075 µg SgHST

Table S13. Assay compositions in tests with SgHQT1. Assays generally were performed in a final volume of 125 µl and contained K_2HPO_4/KH_2PO_4 buffer (KP_i), an acyl donor substrate and an acyl acceptor substrate. The tests were started by adding the indicated amount of enzyme and were stopped by adding 20 µl 6 N HCl before extracting the products twice with 0.5 ml ethyl acetate. The collected organic phases were evaporated, and the residue redissolved for HPLC and LC-MS analysis.

**Parameter**		**Conditions**	**Composition**
pH-optimum		40 °C<br>t = 10 min, negative control: t = 0 min<br>*n* = 3	0.1 M KP_i pH 6.00 to 8.50<br>25 µM *p*-coumaroyl-CoA<br>20 mM quinic acid<br>0.324 µg SgHQT1
Temperature optimum		0 to 60 °C<br>t = 2.5 min, negative control: t = 0 min<br>*n* = 3	0.1 M KP_i pH 7.0<br>80 µM caffeoyl-CoA<br>40 mM quinic acid<br>0.722 µg SgHQT1
**K_m for**	**with**		
p-coumaroyl-CoA	quinic acid	40 °C<br>t = 10 min, negative control: t = 0 min<br>*n* = 9	0.1 M KP_i pH 7.0<br>5-200 µM *p*-coumaroyl-CoA<br>80 mM quinic acid pH 7.0<br>0.25 µg SgHQT1
p-coumaroyl-CoA	shikimic acid	40 °C<br>t = 10 min, negative control: t = 0 min<br>*n* = 3	0.1 M KP_i pH 7.0<br>10-400 µM *p*-coumaroyl-CoA<br>320 mM shikimic acid pH 7.0<br>0.25 µg SgHQT1
caffeoyl-CoA	quinic acid	40 °C<br>t = 5 min, negative control: t = 0 min<br>*n* = 9	0.1 M KP_i pH 7.0<br>80 mM quinic acid, pH 7.0<br>5-100 µM caffeoyl-CoA<br>0.25 µg SgHQT1
quinic acid	*p*-coumaroyl-CoA	40 °C<br>t = 10 min, negative control: t = 0 min<br>*n* = 9	0.1 M KP_i pH 7.0<br>200 µM *p*-coumaroyl-CoA<br>1-80 mM quinic acid pH 7.0<br>0.25 µg SgHQT1
quinic acid	caffeoyl-CoA	40 °C<br>t = 5 min, negative control: t = 0 min<br>*n* = 9	0.1 M KP_i pH 7.0<br>100 µM caffeoyl-CoA<br>1-80 mM quinic acid pH 7.0<br>0.25 µg SgHQT1
shikimic acid	*p*-coumaroyl-CoA	40 °C<br>t = 5 min, negative control: t = 0 min<br>*n* = 3	0.1 M KP_i pH 7.0<br>400 µM *p*-coumaroyl-CoA<br>10-320 mM shikimic acid pH 7.0<br>0.5 µg SgHQT1

Table S14. Assay compositions in tests with SgHQT2. Assays generally were performed in a final volume of 125 µl and contained K_2HPO_4/KH_2PO_4 buffer (KP_i), an acyl donor substrate and an acyl acceptor substrate. The tests were started by adding the indicated amount of enzyme and were stopped by adding 20 µl 6 N HCl before extracting the products twice with 0.5 ml ethyl acetate. The collected organic phases were evaporated, and the residue redissolved for HPLC and LC-MS analysis.

**Parameter**		**Conditions**	**Composition**
pH-optimum (KP_i)		30 °C<br>t = 10 min, negative control: t = 0 min<br>*n* = 3	0.1 M KP_i pH 6.00 to 8.50<br>80 µM *p*-coumaroyl-CoA<br>8 mM quinic acid<br>0.6512 µg SgHQT2
pH-optimum (Tris/HCl)		30 °<br>t = 10 min, negative control: t = 0 min<br>*n* = 3	0.1 M Tris/HCl pH 7.00 to 9.50<br>80 µM *p*-coumaroyl-CoA<br>8 mM quinic acid<br>0.6512 µg SgHQT2
Temperature optimum		5 to 50 °C<br>t = 10 min, negative control: t = 0 min<br>*n* = 3	0.1 M KP_i pH 7.5<br>80 µM *p*-coumaroyl-CoA<br>8 mM quinic acid<br>3.256 µg SgHQT2
**K_m for**	**with**		
p-coumaroyl-CoA	quinic acid	30 °C<br>t = 15 min, negative control: t = 0 min<br>*n* = 9	0.1 M KP_i pH 7.5<br>25-400 µM *p*-coumaroyl-CoA<br>160 mM quinic acid pH 7.0<br>0.6512 µg SgHQT2
Caffeoyl-CoA	quinic acid	30 °C<br>t = 30 min, negative control: t = 0 min<br>*n* = 9	0.1 M KP_i pH 7.5<br>50-800 µM caffeoyl-CoA<br>160 mM quinic acid pH 7.0<br>1.0 µg SgHQT2
quinic acid	*p*-coumaroyl-CoA	30 °C<br>t = 15 min, negative control: t = 0 min<br>*n* = 9	0.1 M KP_i pH 7.5<br>400 µM *p*-coumaroyl-CoA<br>1-160 mM quinic acid pH 7.0<br>0.6512 µg SgHQT2
quinic acid	caffeoyl-CoA	30 °C<br>t = 15 min, negative control: t = 0 min<br>*n* = 9	0.1 M KP_i pH 7.5<br>800 µM caffeoyl-CoA<br>1-160 mM quinic acid pH 7.0<br>1.0 µg SgHQT2

Table S15. Assay compositions in tests with SgRAS. Assays generally were performed in a final volume of 125 µl and contained K_2HPO_4/KH_2PO_4 buffer (KP_i), an acyl donor substrate and an acyl acceptor substrate. The tests were started by adding the indicated amount of enzyme and were stopped by adding 20 µl 6 N HCl before extracting the products twice with 0.5 ml ethyl acetate. The collected organic phases were evaporated, and the residue redissolved for HPLC and LC-MS analysis.

**Parameter**		**Conditions**	**Composition**
pH-optimum		30 °C<br>t = 10 min, negative control: t = 0 min<br>*n* = 3	0.1 M KP_i pH 6.0 to 8.5<br>200 µM caffeoyl-CoA<br>4 mM 4-hydroxyphenyllactic acid<br>7.380 µg SgRAS
Temperature optimum		0 to 70 °C<br>t = 20 min, negative control: t = 0 min<br>*n* = 3	0.1 M KP_i pH 8.0<br>200 µM caffeoyl-CoA<br>4 mM 4-hydroxyphenyllactic acid<br>3.690 µg SgRAS
**K_m for**	**with**		
p-coumaroyl-CoA	4-hydroxy-phenyllactic acid	25 °C<br>t = 15 min, negative control: t = 0 min<br>*n* = 9	0.1 M KP_i pH 8.0<br>2.5-300 µM *p*-coumaroyl-CoA<br>4 mM 4-hydroxyphenyllactic acid<br>0.190/0.382/0.379 µg SgRAS
p-coumaroyl-CoA	3,4-dihydroxy-phenyllactic acid	25 °C<br>t = 5 min, negative control: t = 0 min<br>*n* = 9	0.1 M KP_i pH 8.0<br>2.5-100 µM *p*-coumaroyl-CoA<br>4 mM 3,4-dihydroxyphenyl-lactic acid<br>0.2 µg SgRAS
caffeoyl-CoA	4-hydroxy-phenyllactic acid	25 °C<br>t = 60 min, negative control: t = 0 min<br>*n* = 9	0.1 M KP_i pH 8.0<br>4 mM 4-hydroxyphenyllactic acid<br>10-640 µM caffeoyl-CoA<br>1.843/1.284 µg SgRAS
4-hydroxy-phenyllactic acid	*p*-coumaroyl-CoA	25 °C<br>t = 20 min, negative control: t = 0 min<br>*n* = 9	0.1 M KP_i pH 8.0<br>0.2 mM *p*-coumaroyl-CoA<br>0.1-7 mM 4-hydroxyphenyl-lactic acid<br>1.0 µg SgRAS
4-hydroxy-phenyllactic acid	caffeoyl-CoA	25 °C<br>t = 40 min, negative control: t = 0 min<br>*n* = 9	0.1 M KP_i pH 8.0<br>0.4 mM caffeoyl-CoA<br>0.1-7 mM 4-hydroxyphenyl-lactic acid<br>0.380/0.382/0.379 µg SgRAS
3,4-dihydroxy-phenyllactic acid	*p*-coumaroyl-CoA	25 °C<br>t = 20 min, negative control: t = 0 min<br>*n* = 9	0.1 M KP_i pH 8.0<br>0.1 mM *p*-coumaroyl-CoA<br>0.1-7 mM 3,4-dihydroxy-phenyllactic acid<br>1.0 µg SgRAS

Table S16. List of authentic standards used for identification and quantification, and their commercial suppliers or the origin of their isolation

Authentic standard	Source information
caffeic acid	Carl Roth
4-coumaric acid	Sigma-Aldrich
caffeoyl-3',4'-dihydroxyphenyllactic acid = rosmarinic acid	AG Petersen (isolated from *Melissa officinalis*)
caffeoyl-3-*O*-quinic acid = neochlorogenic acid	Phyproof
caffeoyl-3-*O*-shikimic acid	Akos
caffeoyl-4'-hydroxyphenyllactic acid = isorinic acid	AG Petersen (isolated from *Melissa officinalis* cell cultures, see Ernst *et al.* (2022))
caffeoyl-4-*O*-quinic acid = cryptochlorogenic acid	Sigma Aldrich
caffeoyl-4-*O*-shikimic acid	Akos
caffeoyl-5-*O*-quinic acid = chlorogenic acid	Carl Roth
caffeoyl-5-*O*-shikimic acid	BenchChem
p-coumaroyl-2-*N*-3-hydroxyanthranilic acid	AG Petersen (synthesized according to Alber *et al.* (2019), see Ernst *et al.* (2022))
p-coumaroyl-3',4'-dihydroxyphenyllactic acid	AG Petersen (isolated from *Melissa officinalis* cell cultures, see Ernst *et al.* (2022))
p-coumaroyl-3-*O*-quinic acid	BenchChem
p-coumaroyl-4'-hydroxyphenyllactic acid	AG Petersen (isolated from *Melissa officinalis* cell cultures, see Ernst *et al.* (2022))
p-coumaroyl-4-*O*-quinic acid	BenchChem
p-coumaroyl-5-*O*-quinic acid	TargetMol
p-coumaroyl-5-*O*-shikimic acid	AG Petersen (synthesized enzymatically)
rosmarinic acid 3'-*O*-β-D-glucoside	AG Petersen (isolated from *Anthoceros agrestis* cell cultures, see Vogelsang *et al.* (2006))

Table S17. LC-MS analysis of authentic standards, including retention times, molecular formulas, detected $[M-H]^-$ (*m/z*) as well as LC/ESI-MS/MS *m/z* (% base peak, only >1%). All standards were measured using the short method (see Experimental procedures) in the negative ionization mode for the verification of product formation in enzyme essays.

**Authentic standard**	**Retention time [min]**	**Molecular formula**	**Detected $[M-H]^-$ (*m/z*)**
caffeoyl-3',4'-dihydroxyphenyllactic acid = rosmarinic acid	8.3	$C_{18}H_{16}O_8$	359.08
caffeoyl-3-*O*-quinic acid = neochlorogenic acid	6.1	$C_{16}H_{18}O_9$	353.09
caffeoyl-3-*O*-shikimic acid	7.2	$C_{16}H_{16}O_8$	335.08
caffeoyl-4'-hydroxyphenyllactic acid = isorinic acid	8.8	$C_{18}H_{16}O_7$	343.08
caffeoyl-4-*O*-quinic acid = cryptochlorogenic acid	6.6	$C_{16}H_{18}O_9$	353.09
caffeoyl-4-*O*-shikimic acid	7.2	$C_{16}H_{16}O_8$	335.08
caffeoyl-5-*O*-quinic acid = chlorogenic acid	6.6	$C_{16}H_{18}O_9$	353.09
caffeoyl-5-*O*-shikimic acid	7.2	$C_{16}H_{16}O_8$	335.08
p-coumaroyl-2-*N*-3-hydroxyanthranilic acid	9.9	$C_{16}H_{13}O_5N_1$	299.08
p-coumaroyl-3',4'-dihydroxyphenyllactic acid	8.8	$C_{18}H_{16}O_7$	343.08
p-coumaroyl-3-*O*-quinic acid	6.5	$C_{16}H_{18}O_8$	337.09
p-coumaroyl-4'-hydroxyphenyllactic acid	9.2	$C_{18}H_{16}O_6$	327.09
p-coumaroyl-4-*O*-quinic acid	7.1	$C_{16}H_{18}O_8$	337.09
p-coumaroyl-5-*O*-quinic acid	7.1	$C_{16}H_{18}O_8$	337.09
p-coumaroyl-5-*O*-shikimic acid	7.7	$C_{16}H_{16}O_7$	319.08

Table S18. Primer sequences and PCR conditions for the amplification of hydroxycinnamoyltransferase sequences in *Sarcandra glabra*. The scaffolds were retrieved in the 1kP database by searching with the rosmarinic acid synthase sequence of *Coleus blumei* (UniProt A0PDV5). Restriction sites for ligation into the expression plasmid pET-15b are written in italics.

**Enzyme**	**Primers**	**PCR**	**Composition**
OSHQ_ scaffold 2009492<br><br>**SgHCT-A**<br>**SgHST**	f: TAA*CATATG*AAGATGTTAATCAACG TGAGGG<br>r: TA*GGATCC*TTAAATATCGTAGAAAA ACTTCTGGAATCG	94 °C 90 s,<br>[94 °C 30 s, 57 °C 30 s, 68 °C 90 s] x40,<br>68 °C 510 s	1.0 µl cDNA, 2.5 µl buffer I, 0.5 µl of each primer (100 µM), 0.1 µl AccuPrime polymerase, 16.2 µl H_2O
OSHQ_ scaffold 2009493<br><br>**SgHCT-B**	f: TAA*CATATG*ATCGTCAAGCTCAAAG AGTCTA<br>r: TA*GGATCC*TTAAATATCGTAGAAAA ACTTCTGGAATCG	94 °C 90 s,<br>[94 °C 30 s, 57 °C 30 s, 68 °C 90 s] x40,<br>68 °C 510 s	1.0 µl cDNA, 2.5 µl buffer I, 0.5 µl of each primer (100 µM), 0.1 µl AccuPrime polymerase, 16.2 µl H_2O

OSHQ_ scaffold 2009494<br><br>**SgHCT-C**<br>**SgHQT1**	f: TAA*CATATG*ATCGTCAAGCTCAAAGAGTCTA<br>r: TA*GGATCC*TCAAAGATCATAGAAAATCTTCTTAAATGACG	94 °C 90 s,<br>[94 °C 30 s, 57 °C 30 s, 68 °C 90 s] x40,<br>68 °C 510 s	1.0 µl cDNA, 2.5 µl buffer I, 0.5 µl of each primer (100 µM), 0.1 µl AccuPrime polymerase, 16.2 µl H_2O
OSHQ_ scaffold 2048693<br><br>**SgHCT-D**<br>**SgRAS**	f: TAA*CATATG*GCGACCATCAAGACTTCC<br>r: TA*GGATCC*CTAGAGACCTTGGTAAAAAAATTGCTTG	94 °C 90 s,<br>[94 °C 30 s, 57 °C 30 s, 68 °C 90 s] x40,<br>68 °C 510 s	1.0 µl cDNA, 2.5 µl buffer I, 0.5 µl of each primer (100 µM), 0.1 µl AccuPrime polymerase, 16.2 µl H_2O
OSHQ_ scaffold 2009853<br><br>**SgHCT-E**<br>**SgHQT2**	5´-RACE:<br>f: UPM (SMARTer RACE kit) TAATACGACTCACTATAGGGCAAGCAGTGGTATCAACGCAGAGT<br>TCATAACTCGTAAAAGAGCTTACTAAATTG<br>r: TAATACGACTCACTATAGGGCAAGCAGTGGTATCAACGCAGAGT<br><br>Full sequence:<br>f: ATT*CTCGAG*ATGGGGAATAGTTTTCATAGAAGAAGC<br>r: TAT*CTCGAG*CTATAACTCGTAAAAGAGCTTACTAAATTGCC	5´-RACE:<br>[94 °C 30 s, 68 °C 30 s, 72 °C 90 s] x5,<br>[94 °C 30 s, 66 °C 30 s, 72 °C 90 s] x5,<br>[94 °C 30 s, 64/62/60 °C 30 s, 72 °C 90 s] x30<br><br>Full sequence:<br>94 °C 120 s,<br>[94 °C 30 s, 60/58 °C 30 s, 68 °C 90 s] x40,<br>68 °C 30 s	5´-RACE:<br>4.0 µl 5´RACE-ready cDNA, 12.5 µl buffer I, 0.5 µl primer (100 µM), 2.5 µl 10 x UPM, 0.5 µl SeqAmp polymerase, 5.0 µl H_2O<br><br>Full sequence:<br>4.0 µl cDNA, 2.5 µl buffer I, 0.5 µl of each primer (100 µM), 0.1 µl AccuPrime Polymerase, 17.4 µl H_2O
OSHQ_ scaffold 2048698<br><br>**SgHCT-F**	f: TAA*CATATG*GCCTCTTCTTTAGTATTCTCA<br>r: TAA*CATATG*CTAAAGCGCAGATATGATGAA	94 °C 90,<br>[94 °C 30 s, 58 °C 30 s, 68 °C 90 s] x40,<br>68 °C 510 s	1.0 µl cDNA, 2.5 µl buffer I, 0.5 µl of each primer (100 µM), 0.1 µl AccuPrime Polymerase, 16.2 µl H_2O

Table S19. Primer sequences for gDNA analysis of SgHCTs and PCR conditions. Sequences containing an intron are marked with *.

Sequence	Length of amplicon [bp]	Primers	Composition of assay and cycling program
SgHST	1275 (partial)	f: GCGGCGCAGACGCCTAACCAT<br>r: TTAAATATCGTAGAAAAACTTCTGGAATCGTAGCATGTG	1.0 µl gDNA<br>5.0 µl GoTaq buffer<br>3.0 µl $MgCl_2$ (25mM)<br>0.5 µl dNTPs (10 mM)<br>0.5 µl of each primer (10 µM)<br>0.1 µl GoTaq G2 polymerase<br>14.4 µl H_2O<br><br>94 °C 180 s, [94 °C 30 s, 63 °C 60 s, 72 °C 120 s] 40x, 70 °C 180 s

SgHQT1	1286	f: TAACATATGATCGTCAAGCTCAAAGAGTCTA<br>r: TAGGATCCTCAAAGATCATAGAAAATCTTCTTAAATGACG	1.0 µl gDNA<br>5.0 µl GoTaq buffer<br>3.0 µl $MgCl_2$ (25mM)<br>0.5 µl dNTPs (10 mM)<br>0.5 µl of each primer (10 µM)<br>0.1 µl GoTaq G2 polymerase<br>14.4 µl H_2O<br><br>94 °C 180 s, [94 °C 30 s, 57 °C 60 s, 72 °C 120 s] 40x, 70 °C 180 s
SgRAS	1350	f: TAACATATGGCGACCATCAAGACTTCC<br>r: TAGGATCCCTAGAGACCTTGGTAAAAAAATTGCTTG	1.0 µl gDNA<br>5.0 µl GoTaq buffer<br>3.0 µl $MgCl_2$ (25mM)<br>0.5 µl dNTPs (10 mM)<br>0.5 µl of each primer (100 µM)<br>0.1 µl GoTaq G2 polymerase<br>14.4 µl H_2O<br><br>[94 °C 30 s, 68 °C 60 s, 72 °C 180 s] 40x, [94 °C 30 s, 59 °C 60 s, 70 °C 180 s] 5x
SgHCT-F	1468* (91 bp Q-intron)	f: TAACATATGGCCTCTTCTTTAGTATTCTCA<br>r: TAACATATGCTAAAGCGCAGATATGATGAA	1.0 µl gDNA<br>5.0 µl GoTaq buffer<br>3.0 µl $MgCl_2$ (25mM)<br>0.5 µl dNTPs (10 mM)<br>0.5 µl of each primer (100 µM)<br>0.1 µl GoTaq G2 polymerase<br>14.4 µl H_2O<br><br>[94 °C 30 s, 68 °C 60 s, 72 °C 180 s] 40x, [94 °C 30 s, 59 °C 60 s, 70 °C 180 s] 5x

Table S20. Primer sequences and PCR conditions for expression analysis. Primers were designed for regions of SgHCTs with highest disparity, using the PCR Primer Design Tool by Eurofins Genomics (https://eurofinsgenomics.eu/en/ecom/tools/pcr-primer-design/).

Sequence	Amplified region [bp]	Primers	Composition of assay and cycling program
SgHST	57-256	f: GCCTAACCATAGCCTGTGGAAC<br>r: TCTCTATCCGTCCATCTTCGTCTC	1.0 µl cDNA diluted 1:10<br>5.0 µl GoTaq buffer I<br>3.0 µl $MgCl_2$ (25 mM)<br>0.5 µl dNTPs (10 mM)<br>0.5 µl of each primer (10 µM)<br>0.1 µl GoTaq G2 polymerase<br>14.4 µl H_2O<br><br>94 °C 120 s
SgHQT1	651-838	f: ATGCCAACCAGATCCCGCAG<br>r: GGCCGTCAGTGGCGATAAATAG	
SgRAS	392-590	f: CGTTGCTTTTGGTGCAGTACAC<br>r: TCTCTTCCCTTCAGAACCGTCC	
SgHCT-F	295-502	f: GAGGGCGTGATGTTCATTGAGG<br>r: TTATGGTGTGGTTGAGGCGGAG	
SgAct-1	288-477	f: CCGAGTAGCTCCAGAAGAACATCC	

		r: GTCACCAGAATCCAGCACAATACC	[94 °C 30 s, 59°C 30 s, 72 °C 20 s] x35
SgHQT2	411-608	f: GCCATGCCCGTTGCTATTAATTC<br>r: GGAGGTTTACGAGCACGAAGG	1.0 µl cDNA<br>5.0 µl GoTaq buffer I<br>3.0 µl $MgCl_2$ (25 mM)<br>0.5 µl dNTPs (10 mM)<br>0.5 µl of each primer (10 µM)<br>0.1 µl GoTaq G2 polymerase<br>14.4 µl H_2O<br><br>94 °C 120 s<br>[94 °C 30 s, 59°C 30 s, 72 °C 20 s] x40

References

Alber, A.V., Renault, H., Basilio-Lopes, A., Bassard, J.-E., Liu, Z., Ullmann, P., Lesot, A., Bihel, F., Schmitt, M., Werck-Reichhart, D. and Ehlting, J. (2019) Evolution of coumaroyl conjugate 3-hydroxylases in land plants: lignin biosynthesis and defense. *Plant J.*, 99, 924–936. https://doi.org/10.1111/tpj.14373.

Berger, A., Meinhard, J. and Petersen, M. (2006) Rosmarinic acid synthase is a new member of the superfamily of BAHD acyltransferases. *Planta*, 224, 1503–1510. doi.org/10.1007/s00425-006-0393-y.

Ernst, L., Wohl, J., Bauerbach, E. and Petersen, M. (2022) Hydroxycinnamoyltransferase and CYP98 in phenolic metabolism in the rosmarinic acid-producing hornwort *Anthoceros agrestis*. *Planta*, 255, 75. https://doi.org/10.1007/s00425-022-03856-9

Kruse, L.H., Weigle, A.T., Irfan, M., Martínez-Gómez, J., Chobirko, J.D., Schaffer, J.E., Bennett, A.A., Specht, C.D., Jez, J.M., Shukla, D. and Moghe, G.D. (2022) Orthology-based analysis helps map evolutionary diversification and predict substrate class use of BAHD acyltransferases. *Plant J.*, 111, 1453–1468. https://doi.org/10.1111/tpj.15902.

Moghe, G., Kruse, L.H., Petersen, M., Scossa, F., Fernie, A.R., Gaquerel, E. and D'Auria, J.C. (2023) BAHD Company: The Ever-Expanding Roles of the BAHD Acyltransferase Gene Family in Plants. *Annu. Rev. Plant Biol.*, 74, 165–194. https://doi.org/10.1146/annurev-arplant-062922-050122.

Tamura, K., Stecher, G. and Kumar, S. (2021) MEGA11: Molecular Evolutionary Genetics Analysis Version 11. *Mol. Biol. Evol.*, 38, 3022–3027. https://doi.org/10.1093/molbev/msab120.

Vogelsang, K., Schneider, B. and Petersen, M. (2006) Production of rosmarinic acid and a new rosmarinic acid 3'-*O*-β-D-glucoside in suspension cultures of the hornwort *Anthoceros agrestis* Paton. *Planta*, 223, 369–373. doi.org/10.1007/s00425-005-0089-8.

Wang, P.P., Liu, H., Gao, S. and Cheng, A.X. (2017) Functional Characterization of a Hydroxyacid/Alcohol Hydroxycinnamoyl Transferase Produced by the Liverwort *Marchantia emarginata*. *Molecules*, 22, 1854. https://doi.org/10.3390/molecules22111854.

Zhou, J., Zou, X., Deng, Z. and Duan, L. (2024) Analysing a Group of Homologous BAHD Enzymes Provides Insights into the Evolutionary Transition of Rosmarinic Acid Synthases from Hydroxycinnamoyl-CoA:Shikimate/Quinate Hydroxycinnamoyl Transferases. *Plants*, 13, 512. https://doi.org/10.3390/plants13040512.
